# Comparative evaluation of gene selection approaches in transcriptomics: bias correction and visualization with TransPro

**DOI:** 10.1093/gigascience/giag057

**Published:** 2026-05-18

**Authors:** Dongyue Yu, Chen Li, Shuo Yan, Lujiale Guo, Jingyu Liang, Shengquan Chen, Wenjun Bu

**Affiliations:** Institute of Entomology, College of Life Sciences, Nankai University, Weijin Road, Nankai District, Tianjin 300071, China; Tianjin Medical University Cancer Institute and Hospital, Huanhu West Road, Hexi District, Tianjin 300060, China; AI Thrust, The Hong Kong University of Science and Technology (Guangzhou), Duxue Road, Nansha District, Guangzhou 510000, China; Zhongshan Hospital of Fudan University, Fenglin Road, Xuhui District, Shanghai 200032, China; Institute of Entomology, College of Life Sciences, Nankai University, Weijin Road, Nankai District, Tianjin 300071, China; School of Mathematical Sciences, LPMC, and AAIS, Nankai University, Weijin Road, Nankai District, Tianjin 300071, China; Institute of Entomology, College of Life Sciences, Nankai University, Weijin Road, Nankai District, Tianjin 300071, China

**Keywords:** differential gene selection, transcriptomics, correlation bias, multivariate feature selection, standardized workflow, multidimensional visualization, gene set enrichment analysis

## Abstract

**Background:**

Differential gene selection is fundamental to transcriptomics; however, mainstream methods typically fit each gene independently via marginal analyses. Although multifactor designs can be accommodated, gene–gene interactions are not explicitly modeled, rendering results susceptible to systematic bias driven by coexpression patterns. Additionally, heterogeneous tools, inconsistent metrics, and fragmented workflows compromise the reproducibility and interpretability of downstream enrichment and visualization analyses.

**Findings:**

We developed TransPro, an open-source integrated framework comprising 2 complementary packages, TransProPy and TransProR, for systematic benchmarking, bias correction, and reproducible visualization in differential gene selection. TransProPy (Python) combines multivariate AUC-based complementarity quantification with ensemble learning for interaction-aware gene selection, whereas TransProR (R) provides standardized differential analysis, pathway enrichment, and 7 visualization workflows (circular dendrograms, chord diagrams, spiral plots, and interaction networks). Cross-dataset generalizability was assessed using 12 independent datasets spanning multiple cancer types and normal tissues with broad heterogeneity in data origin, platform, batch structure, and sample size. Under stringent thresholds, positively and negatively correlated genes maintained a near-equal proportion at the gene level, and activated and suppressed pathway proportions exhibited good concordance with gene-level correlation patterns; critically, TransProPy produced meaningful enrichment results under conditions where conventional methods failed. Quantitative comparisons of core-enriched gene proportions further supported these differences (Kruskal–Wallis and pairwise Wilcoxon tests, *P* < 0.001).

**Conclusions:**

TransPro establishes a unified, reproducible framework that corrects method-specific biases while bridging computational discovery and biological interpretation. All code, workflows, documentation, and example data are openly accessible to support reproducibility and community reuse.

## Graphical Abstract

**Graphical Abstract:**
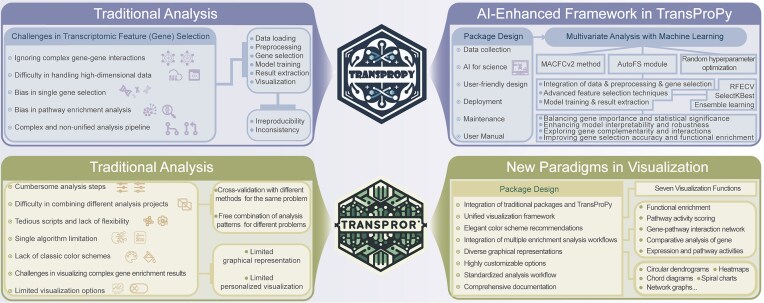

## Introduction

The identification of functional genes associated with specific biological processes or diseases is a cornerstone of modern genomic research, providing the foundation for understanding complex biological mechanisms and developing targeted therapies. Feature selection plays a particularly crucial role in this process, referring to the extraction of the most informative genes (features) from large-scale, high-dimensional biological datasets, such as bulk RNA-Seq [1], single-cell RNA-Seq [2–4], and microarray data [5]. These features typically represent gene expression levels and are selected based on their relevance and statistical significance in distinguishing between different phenotypes, such as healthy and diseased states. Traditionally, feature selection methods have focused on identifying genes that are independently associated with particular phenotypes. While this approach can be effective in certain contexts, it often falls short when it comes to capturing the intricate interactions among genes that contribute to the full expression of biological traits. Genes do not operate in isolation; they interact within complex networks, where the collective effect of multiple genes provides deeper insights into biological processes than any single gene alone.

In practical transcriptomic workflows, differential expression (DE) analysis is also frequently used as an initial screening step for feature selection. Although DE analysis and supervised feature selection do not serve the same primary objective, both can provide candidate gene sets for downstream transcriptomic analyses. DE methods are primarily designed to answer “which genes change significantly on their own,” whereas phenotype discrimination often depends on multigene combinations whose joint effect may be weak or invisible in marginal, gene-by-gene tests. Therefore, while DE-based rankings remain indispensable baselines, advancing transcriptomic feature selection requires methods that explicitly consider multivariate complementarity and interaction effects.

In the field of gene selection and DE analysis, DESeq2 [6], edgeR [7], limma [8, 9], and the Wilcoxon rank-sum test (WRST) [10] are widely used classical methods. These approaches are based on statistical models that assess changes in the expression levels of individual genes. Although these methods are effective in standard DE settings, they exhibit limitations when applied to complex biological systems and high-dimensional datasets. DESeq2 and edgeR are built on the negative binomial distribution, making them especially suitable for modeling count-based RNA-Seq data. By incorporating overdispersion parameters, they account for biological and technical variability, enhancing model flexibility. However, in cases where data noise is high or sample distributions deviate from the negative binomial assumption, these methods may not fully capture the data’s complexity. Although DESeq2 incorporates Cook’s distance-based outlier detection, extreme distributional violations can still compromise model fitting. Additionally, they tend to prioritize highly expressed genes, while lowly expressed or rare genes often remain undetected, contributing to an increased false negative rate. By comparison, limma, which is based on a linear model, is adept at handling various types of high-throughput data such as RNA-Seq and microarrays. Although it excels in estimating gene expression changes, the model’s simplifications can introduce bias when dealing with complex gene interactions, as each gene is tested marginally without explicit modeling of inter-gene dependencies. The WRST, a non-parametric method, is appropriate for data that do not meet the normality assumption, but as a univariate test, it cannot capture synergistic effects between genes, and its discovery rate is further reduced by large-scale multiple-testing correction.

To address these limitations, advanced methods have made important progress along different directions. The MACFC algorithm [11] introduced the multi-variable Area Under the Curve (mvAUC) metric for identifying complementary gene features and showed improved performance over FAST [12], ARCO [13], AVC [14], ReliefF [15], mRMR [16], MRI [17], and PAM [18] in benchmark evaluations across UCI, TCGA [19], and microarray platforms. In parallel, Automated Machine Learning frameworks, including AutoGluon-Tabular [20], Auto-WEKA [21, 22], and auto-sklearn [23], have improved analytical efficiency through automated model selection and optimization. Yet transcriptomic research still faces systemic rather than isolated challenges. Existing tools often perform well in individual tasks, such as feature selection, modeling, or visualization, but rarely provide a unified workflow for balanced gene selection, interpretable pathway analysis, and integrated cross-method visualization. TransPro was developed to address this gap by combining complementarity-aware feature selection with standardized downstream analysis and multi-dimensional visualization.

**Figure 1:**
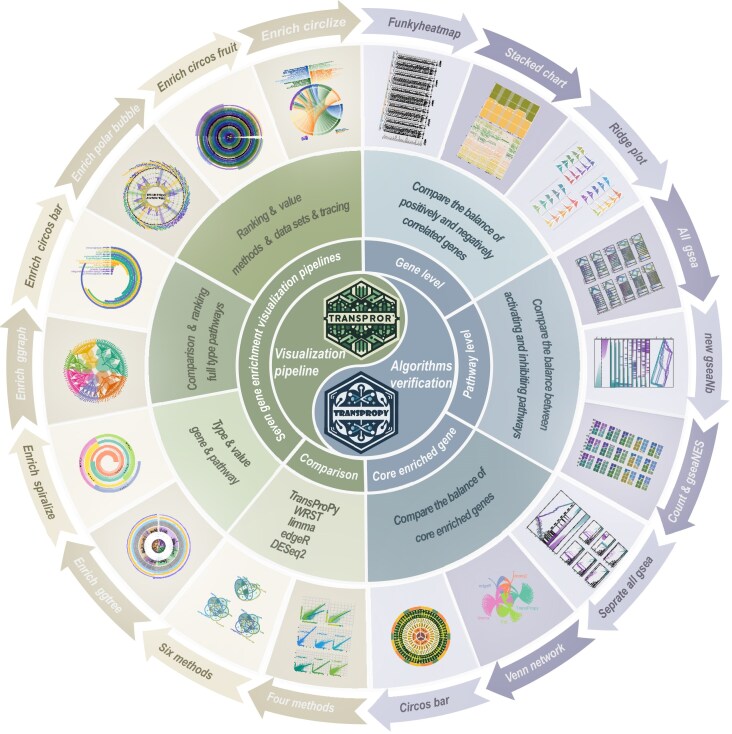
Schematic layout of the TransPro project. The circular diagram provides an overview of the TransProR and TransProPy workflows for gene enrichment and pathway analysis. The center highlights the 2 core modules for multilevel visualization and algorithm validation. The middle ring shows 3 analytical domains: gene level, core-enriched gene level, and pathway level. These domains support evaluation of dataset performance, comparison of core-enriched gene balance, and assessment of activation and inhibition pathway balance. The outer ring summarizes validation across TransProPy, DESeq2, edgeR, limma, and the WRST, and illustrates 7 visualization pipelines: Enrich ggtree, Enrich spiralize, Enrich ggraph, Enrich circos bar, Enrich polar bubble, Enrich circos fruit, and Enrich circlize.

Building on these insights, the TransPro project has been developed to extend these advanced methodologies into the realm of transcriptomics data analysis. While not an AutoML system, TransProPy (v1.0.0)—the Python-based toolkit within the TransPro project—adopts a similar philosophy by integrating advanced machine learning techniques and multivariate interaction analyses. TransProPy is specifically designed to evaluate the complementarity and interactions among gene features, thereby addressing the limitations of traditional univariate approaches. This focus on complex gene interactions within transcriptomics data ensures that TransProPy meets the specific demands of transcriptomics analysis. Further advancing these capabilities, TransProR (v1.0.7)—the R-based counterpart—offers a well-integrated suite of visualization tools that seamlessly combine custom workflows and scripts with established R packages. This framework is designed to accommodate diverse data types and scalable analytical requirements, effectively addressing the intrinsic limitations of traditional approaches while enabling researchers to intuitively explore and reconstruct the intricate relationships within gene enrichment data. By combining TransProPy’s robust analytical power with TransProR’s advanced visualization capabilities, TransPro establishes a synergistic and nuanced approach to transcriptomic data analysis. This toolset bridges the gap between machine learning and genomics, promising to play a pivotal role in the future of biological and medical research while offering profound insights into gene function and its impact on health and disease (Fig. 1).

## Findings

### TransProPy achieves balanced positive–negative distribution in gene correlation analysis

**Figure 2:**
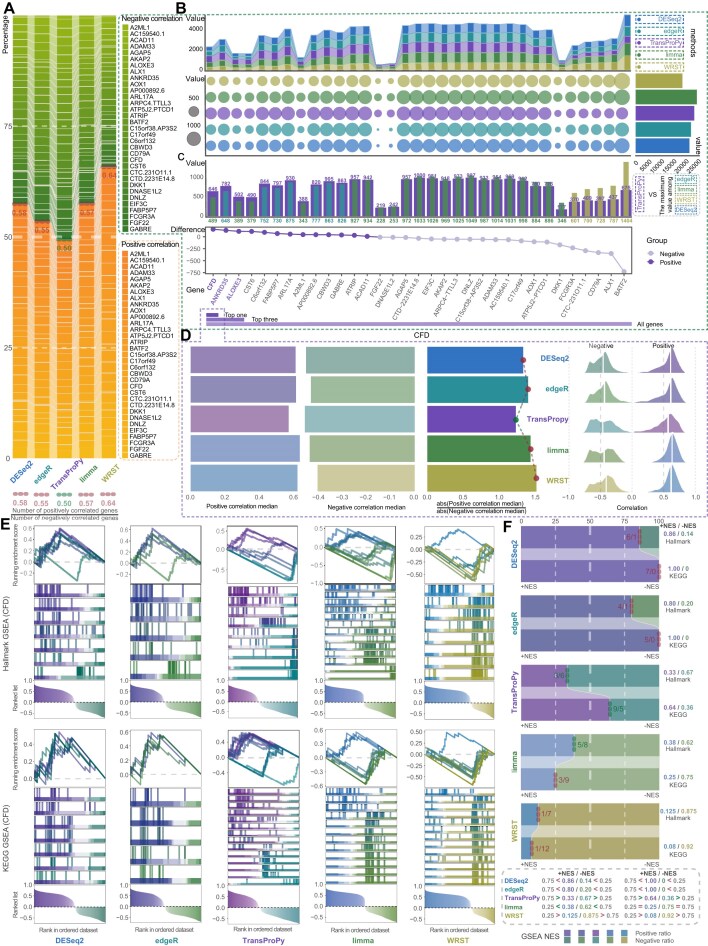
Comprehensive visualization of gene correlation trends and pathway enrichment across multiple gene selection methods. (A) Stacked bar plot illustrating the overall distribution of selected genes from 33 candidates across the 5 methods: DESeq2, edgeR, limma, WRST, and TransProPy. Each bar represents the total number of genes identified, with distinct color sections indicating the proportion of positively and negatively correlated genes. (B) Stacked area plot (top) and bubble chart (bottom) showing the numbers of strongly correlated genes across the 33 genes, illustrated here for negatively correlated genes. The bar chart on the right summarizes the total number of genes selected by each method. (C) For negatively correlated genes, the bar chart (top) compares the number of negatively correlated genes in the TransProPy method with the maximum negatively correlated gene counts from the other 4 methods: edgeR, limma, WRST, and DESeq2, with the differences sorted in descending order (bottom). (D) The ridge density plot illustrates the correlation distribution of the CFD gene across different methods, with the white semi-transparent lines indicating the median position (right). The bar chart shows the magnitude of the median for positive and negative correlations and their ratio (left). (E) Gene set enrichment analysis (GSEA) is performed on the CFD gene using Hallmark and KEGG gene sets, with visualizations that delineate the activation and suppression of pathways. (F) Comparison of the ratio of activated and suppressed pathways across different methods, highlighting the balanced feature selection achieved by TransProPy at the gene pathway level. The +/− NES indicates whether the normalized enrichment score is positive (+NES) or negative (−NES).

All 4 classical methods consistently exhibited an elevated proportion of positively correlated genes in gene correlation analysis, revealing a pronounced imbalance in correlation distribution (Figs 2A and 6G). In contrast, TransProPy yielded the most balanced proportion between positively and negatively correlated genes, highlighting its superior capacity for mitigating correlation bias (Figs 2A and 6G). To further validate this advantage under extreme conditions, we selected the top 3 genes exhibiting the largest inter-method discrepancies—CFD, ANKRD35, and ALOXE3—for in-depth analysis; this ranking was derived from the bottom panel of Fig. 2C, where genes are ordered by the per-gene difference between TransProPy and the strongest of the 4 classical methods in the number of strongly negatively correlated genes.

Among the 3 genes, CFD showed the greatest disparity in negatively correlated gene counts compared with the other methods, with the largest margin relative to the second-ranked method (Fig. 2B, C). Correlation distribution analysis of these 3 genes revealed that all 5 methods displayed bimodal characteristics, with weakly correlated genes being sparse while strongly positive and negative correlations increased sharply; however, conventional methods demonstrated a markedly higher positive correlation peak relative to the negative correlation peak, whereas TransProPy exhibited a correlation distribution symmetrically centered around zero, with the ratio of the absolute positive-correlation median to the absolute negative-correlation median closest to 1 (Fig. 2D; Supplementary Figs 1–3). Even under these maximum contrast intensities, TransProPy maintained a balanced positive–negative distribution while identifying strongly correlated genes, further substantiating its practical applicability. We next examined whether this gene-level balance was maintained at the pathway level.

### TransProPy demonstrates unbiased bidirectional detection across gene and pathway levels

**Figure 3:**
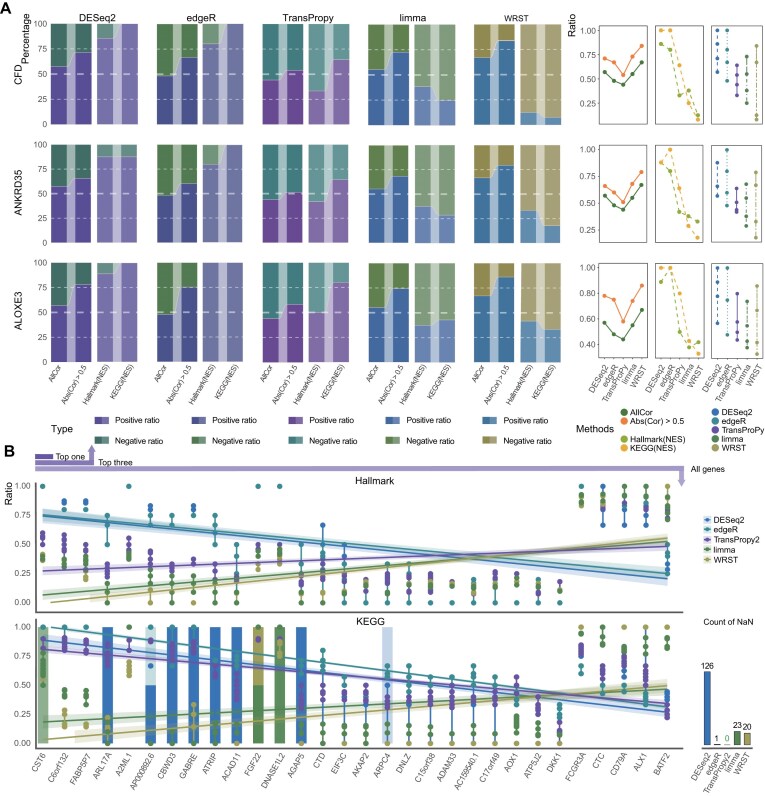
Comprehensive analysis of pathway enrichment and pathway type distribution across different methods. (A) The stacked bar graphs display the top 3 genes from Fig. 2C, categorized by pathway type (Hallmark and KEGG), illustrating the variations in pathway activation and suppression across different methods (DESeq2, edgeR, TransProPy, limma, WRST). Adjacent line charts highlight the dynamic changes in the ratios of positively and negatively correlated genes or pathways, emphasizing how these ratios evolve with increasing gene correlation strength and varying pathway types. (B) This integrated visualization combines scatter and line plot elements to depict the enrichment analysis of 33 genes across KEGG and Hallmark pathways, iterated 20 times for each method. Scatter points represent the proportion of positively enriched pathways among all positively and negatively enriched pathways for each gene, method, pathway database, and repeated GSEA run, and lines connect repeated values from the same method. Colored bar-like elements indicate missing ratio values (NaN), with colors denoting analytical methods; in the KEGG panel, these segments are stacked to distinguish methods. Apparent transparency or intensity differences mainly reflect cumulative overplotting of semi-transparent missing-value segments rather than an independently encoded variable.

In DESeq2 and edgeR, although the overall proportion of positively and negatively correlated genes approached 0.5, the proportion of positively correlated genes significantly exceeded that of negatively correlated genes when |ρ| > 0.5, indicating that selection bias intensified with increasing correlation strength (Fig. 3A). This trend became more pronounced in GSEA, where the number of activated pathways far exceeded suppressed pathways, and in some cases, no suppressed pathways were detected at all—a pattern sharply contrasting with the proportional trends observed at the gene correlation level. Conversely, although in limma and WRST the proportion of positively correlated genes consistently exceeded that of negatively correlated genes, with the selection bias further amplified when |ρ| > 0.5 (Fig. 3A), the GSEA trend was reversed: activated pathways were fewer than suppressed pathways, and WRST exhibited a marked reduction in activated pathways, clearly contradicting the gene-level trends.

Further analysis revealed that many suppressed pathways enriched by limma and WRST were nearly identical in gene ranking and composition, suggesting these pathways were synonymous or highly similar (i.e., different designations or sub-pathways of the same pathway category) rather than entirely independent pathways (Fig. 2E; Supplementary Figs 4–5). Therefore, the apparent advantage of limma and WRST in enriching more suppressed pathways (Fig. 2F) was of limited significance, as excessive redundancy increased noise in the results. When pathway redundancy is substantial, a higher number of enriched pathways may largely reflect repeated detection of overlapping signals rather than additional biological insight. In contrast, TransProPy exhibited the most balanced performance among the 5 methods: even for genes with |ρ| > 0.5, the proportion of positively and negatively correlated genes remained close to 0.5 with minimal fluctuation, and the ratio of activated to suppressed pathways showed the strongest consistency with gene correlation proportions, both approaching 0.5 (Figs 2E, F and 3A). These findings demonstrate that TransProPy effectively balances feature identification at both gene and pathway levels—encompassing positive and negative gene correlations as well as pathway activation and suppression—thereby preventing result bias arising from data imbalance or algorithmic assumptions while comprehensively accounting for gene behavior and their interactions.

### TransProPy consistently enriches pathways across all test conditions where other methods fail

Comprehensive GSEA was performed on all 33 genes, with each gene analyzed 20 times per method to eliminate random effects. Results revealed that in both KEGG and Hallmark enrichment, the proportions of DESeq2 and edgeR were diametrically opposite to those of limma and WRST, whereas TransProPy exhibited the most balanced performance, occupying an intermediate position (Fig. 3B). In KEGG enrichment, the other 4 methods failed to enrich pathways in certain cases—with DESeq2 being the most severely affected—whereas TransProPy was the only method among those evaluated here that successfully enriched pathways under all tested conditions (Fig. 3B, Supplementary Figs 6–10). The ability of TransProPy to achieve balance in both gene correlation and pathway enrichment analyses highlights its comprehensive and precise methodological advantages, effectively avoiding the bias and redundancy present in other methods, and consolidating its value in revealing the deeper complexities of gene interactions.

### TransProPy identifies both shared core genes and unique signatures to enhance enrichment analysis

**Figure 4:**
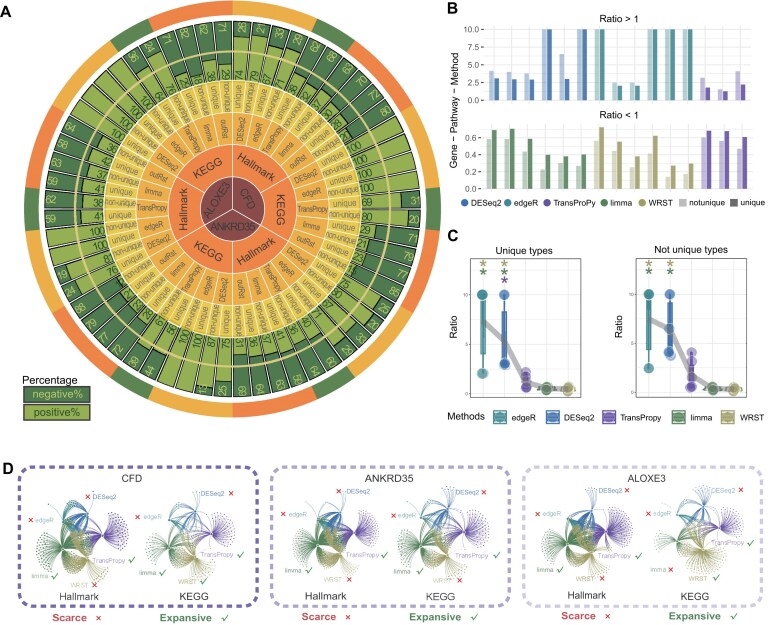
Comparative assessment of gene selection methods in pathway enrichment balance. (A) Each segment of the circos plot depicts the distribution of core-enriched genes by pathway type, comparing deduplicated, and non-deduplicated groups. This visualization highlights super core-enriched genes appearing in multiple pathways across various genomes and methods. (B) Histograms show the distribution of pathway enrichment ratios (positive/negative) for each method, classified by cases where the ratio is greater than or less than 1. (C) Scatter plots comparing the proportions of unique versus non-unique core-enriched genes (Kruskal–Wallis test, *P* < 0.001 for both panels; pairwise comparisons by Wilcoxon test), revealing each method’s gene selection strategy and its influence on the balance between pathway activation and suppression (Supplementary Note 2). (D) Network diagrams show the overlap and connectivity of core-enriched genes across methods for *CFD, ANKRD35*, and *ALOXE3* in Hallmark and KEGG pathways. Check marks (✓) denote methods with sufficient method-specific selected genes, whereas cross marks (×) denote absent or limited method-specific genes.

Detailed examination of gene rankings and enrichment scores within each individual pathway confirmed that no aberrant genes exerted undue influence on pathway-level results. Venn network visualization of core-enriched genes across all significant pathways revealed that, despite algorithmic differences, the 5 methods collectively identified a set of the most central enriched genes. Furthermore, TransProPy, limma, and WRST each possessed unique signature genes that effectively supplemented the analytical results, whereas DESeq2 and edgeR contributed relatively few or no unique genes (Fig. 4D). The lack of additional signature genes to balance the results may be an important reason for the pathway enrichment bias observed in previous analyses—where pathways were predominantly activated with few or no suppressed pathways (Fig. 3A). Therefore, compared with other algorithms, TransProPy not only excels in accurately identifying core-enriched genes but also contributes additional signature genes that effectively address the limitations of other methods. Its balance and comprehensiveness establish it as a critical tool for capturing the complexity of biological regulatory mechanisms, significantly enhancing the robustness and precision of enrichment analysis.

### Quantification of core-enriched genes reveals amplification effects and TransProPy’s balanced detection

Core-enriched genes in activated and suppressed pathways were quantified using both deduplicated and non-deduplicated versions. We summarize directionality using ratios (positive/negative), not only raw counts. Intuitively, pathway overlap acts like a shared “background magnifier”: when overlap affects both directions similarly, ratio-based summaries reduce this global inflation and better reflect baseline directionality. We then compare unique (deduplicated) versus non-unique (non-deduplicated) results to separate stable directional signals from recurrence-driven amplification. Results showed that the imbalanced ratio of negatively to positively regulated genes in the deduplicated group (30 groups) was further amplified in the non-deduplicated group (30 groups), attributable to “super core enriched genes”—genes that recurrently appear across multiple pathways—exerting regulatory roles across a broader range of pathways, thereby appearing repeatedly in the statistics of core-enriched genes across all pathways (Fig. 4A). If the deduplicated group consisted entirely of ordinary core genes or super core genes with identical repetition rates, the gene proportions would remain unchanged in the non-deduplicated group.

However, if the number or repetition rate of super core genes in one category (activated or suppressed) of pathways exceeded that of the other category, the gene proportions would further increase in the non-deduplicated group. An ideal algorithm should identify more super core genes or those with higher repetition rates, indicating greater importance. This effect was evident across all 5 algorithms. Notably, TransProPy was the only method for which ratios both greater than 1 and less than 1 were observed across cases, with each category accounting for half of the observations; by contrast, DESeq2 and edgeR yielded only ratios greater than 1, whereas limma and WRST yielded only ratios less than 1 (Fig. 4B). In both deduplicated and non-deduplicated groups, DESeq2 and edgeR showed proportions of positively correlated core-enriched genes exceeding negatively correlated ones (with some ratios approaching 1), indicating potential bias; limma and WRST showed positively correlated proportions lower than negatively correlated ones (with some positive-to-negative ratios approaching 0); TransProPy’s positive-to-negative ratios fell between those of DESeq2 and edgeR and limma and WRST, demonstrating a smooth transitional trend and yielding the most balanced results (Fig. 4A, C; Kruskal–Wallis *P* < 0.001, Wilcoxon pairwise comparisons).

### Cross-tissue and cross-database validation demonstrates the universal superiority of TransProPy

**Figure 5:**
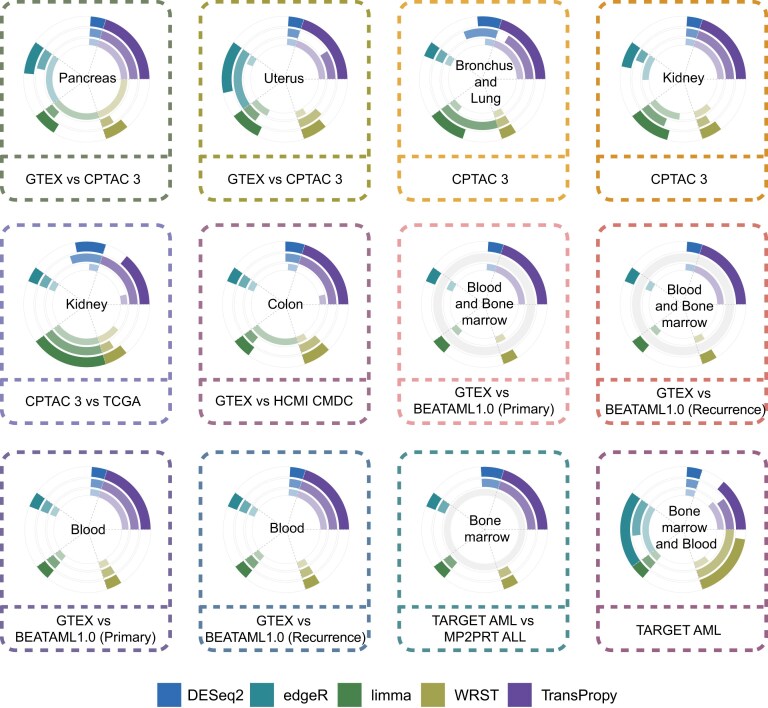
Performance evaluation of gene selection methods across tissues and pathway databases. The panel comprises 12 independent circular plots, each representing a specific tissue-database combination. Each plot contains three concentric rings: the inner ring shows method scores on Hallmark gene sets, the middle ring shows scores on KEGG pathway sets, and the outer ring presents combined scores across both databases. Segments within each ring represent individual analytical methods, as indicated in the figure legend: DESeq2, edgeR, limma, WRST, and TransProPy. Arc lengths are proportional to the corresponding scores, with the outer ring emphasizing total performance. Rings with zero scores are shown with reduced shading (Supplementary Note 1).

To validate the generalizability of our conclusions across broader and more complex biological contexts, we performed comprehensive analyses on multiple datasets from different sources, batches, sample sizes, and biological backgrounds, encompassing pancreatic cancer, uterine cancer, lung cancer, kidney cancer, colon cancer, hematological malignancies, and their corresponding normal tissues. Data were obtained from authoritative projects such as GTEx, CPTAC, TCGA, and TARGET, among others, ensuring the comprehensiveness and generalizability of the evaluation. The circular plots in Fig. 5 comprehensively illustrate the performance of the 5 methods (DESeq2, edgeR, limma, WRST, and TransProPy) across 12 different datasets and tissue types. Each plot adopts a hierarchical ring structure, with concentric rings representing scores for Hallmark, KEGG, and the combined total score from the innermost to the outermost ring, respectively. Arc length is proportional to the score, colors represent different methods, and transparency increases from inner to outer rings to emphasize total score performance; rings with all-zero values are displayed as complete light gray circles. The results demonstrated that in the vast majority of analytical scenarios, the segments corresponding to TransProPy occupied the largest arc length in the outer ring (total score), indicating that the overall performance of TransProPy surpassed that of the other 4 conventional methods across various biological contexts. This extensive validation not only confirmed the superiority of TransProPy on specific datasets but, more importantly, established its reliability as a general-purpose tool across different biological contexts, providing a solid evidence base for its broad application in complex biomedical research.

## Methods

At the methodological level, TransPro is organized into 2 components with distinct functions. TransProPy performs upstream gene selection, complementarity analysis, and method-level bias assessment, whereas TransProR supports downstream differential analysis, enrichment analysis, and visualization. This structure separates computational feature selection from biological interpretation while keeping the workflow integrated and reproducible.

### The MACFC algorithm for feature selection based on mvAUC

TransProPy adopts an mvAUC-based MACFC framework for gene feature selection [11]. Its core principle is global complementarity quantification with redundancy suppression: mvAUC measures the net gain in classification performance when a feature is considered jointly with others, thereby prioritizing synergistic feature combinations rather than relying solely on single>-gene effects.

To accommodate different levels of gene–trait association, 2 complementary implementations are provided: MACFCmain, for weakly associated data, emphasizes broad capture of trait-relevant features; MACFCv2, for strongly associated data, first retains genes with individual AUC values greater than 0.95 as high-confidence discriminatory features. The remaining genes are then evaluated by mvAUC-based complementary selection to identify features that provide additional joint discriminatory information. The final selected feature set combines these high-AUC genes with the mvAUC-selected complementary genes, thereby preserving strong individual signals while allowing lower-AUC but complementary genes to be retained. Using AUC and mvAUC as unified evaluation criteria, the method iteratively constructs optimal feature subsets and outputs ordered features with corresponding weights for downstream modeling and visualization.

### Feature selection strategy based on ensemble models and optimization search

We propose AutoFS, an ensemble- and optimization-based feature selection strategy that builds an end-to-end pipeline unifying data loading and preprocessing, feature selection, model training, and result export. In the selection stage, a FeatureUnion combines recursive feature elimination with cross-validation (RFECV) and SelectKBest: RFECV uses an ensemble estimator of SVM, decision tree, and gradient boosting to iteratively remove redundancy based on cross-validation until the optimal subset is obtained, while SelectKBest complements this with univariate statistical significance to balance model- and statistic-driven signals. RandomizedSearchCV then performs randomized hyperparameter search over a predefined space, and a stacking ensemble (StackingClassifier) fuses predictions from SVM, decision tree, and gradient boosting at the first level, with logistic regression serving as the meta-learner for second-level integration. AutoFS outputs a ranked core feature subset with importances and weights and provides standardized results ready for downstream modeling and visualization, ensuring robustness, interpretability, and generalization across varying data scales and distributions.

### Data acquisition and gene set definition

**Figure 6:**
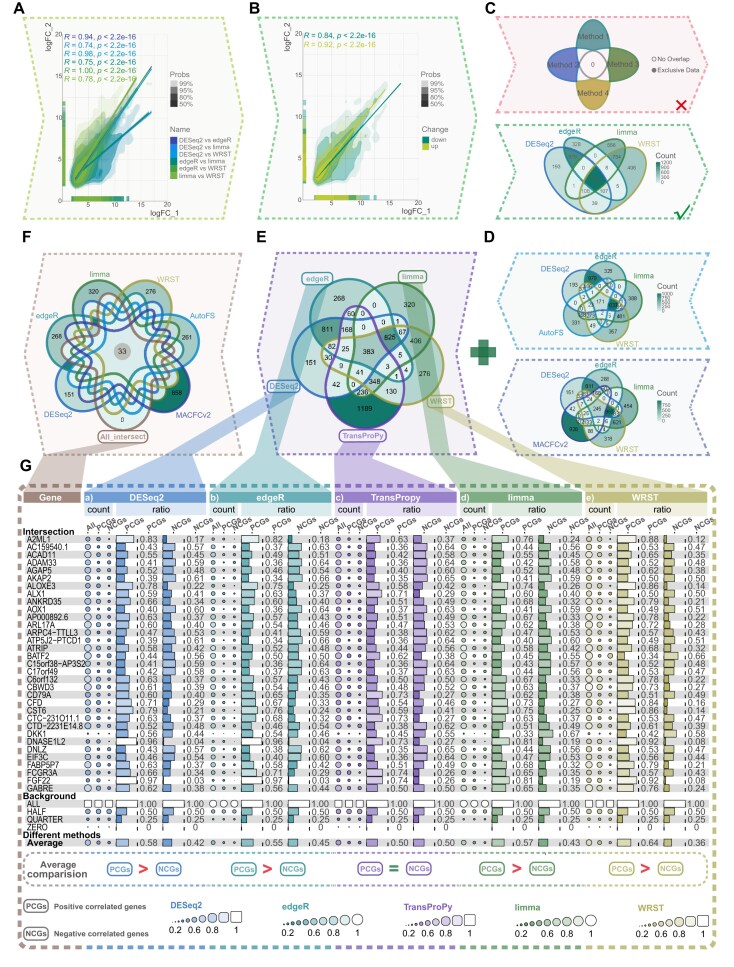
Comparative evaluation of five gene selection methods. (A) Density plot showing pairwise comparisons of log fold-change (logFC) values among four methods: DESeq2, edgeR, limma, and WRST. The density scale represents the degree of overlap among values, with higher-density regions indicating stronger overlap. (B) Density plot comparing logFC values for DESeq2, edgeR, limma, and WRST, grouped into upregulated and downregulated gene categories. The density scale represents the overlap of values within each category, with higher-density areas indicating greater gene density. (C) Venn diagrams showing two representative cases: one with no overlap between methods and another with substantial overlap among DESeq2, edgeR, limma, and WRST in identifying differentially expressed genes (DEGs). (D) Venn diagram showing overlap among DESeq2, edgeR, limma, WRST, and two TransProPy methods, AutoFS and MACFCv2, highlighting shared and method-specific gene selections. (E) Venn diagram showing intersections among DESeq2, edgeR, limma, WRST, and TransProPy, highlighting shared and method-specific DEGs. (F) Venn diagram highlighting the 33 genes consistently selected by all six methods. These genes form a core set of target genes for further feature correlation analysis. (G) Heatmap showing the response of each method to feature correlation. For each gene, the numbers of positively correlated genes (PCGs) and negatively correlated genes (NCGs) were calculated using Spearman’s correlation coefficient (|ρ| > 0.5).

To systematically evaluate the bias of traditional feature selection tools and their impact on downstream analyses, this study utilized TCGA-SKCM [19] (tumor *n* = 470) and GTEx [24] skin tissue (normal *n* = 811) data as examples. Four classical differential analysis methods—DESeq2 [6], edgeR [7], limma [8, 9], and WRST [10]—were evaluated based on pairwise correlations of logFC values (Fig. 6A) and consistency of overall expression trends among differentially expressed genes (Fig. 6B). These methods were selected because they represent widely used DE-based baseline screening strategies in transcriptomic workflows and provide directly comparable gene-level outputs for evaluating selection bias, downstream enrichment, and visualization consistency. They were compared with TransProPy, which integrates AutoFS and MACFCv2. Within TransProPy, MACFCv2 is used for complementarity-aware selection in relatively strongly associated data, whereas AutoFS provides a more general ensemble-based screening strategy for heterogeneous datasets. Through the intersection of all 6 methods, 33 commonly selected genes were identified as the target gene set (Fig. 6C–F), establishing a unified evaluation framework (Fig. 6E). This intersection-based design provides a unified and controlled benchmark, ensuring fair and interpretable comparison across methods by performing all downstream analyses on the same shared gene set.

### Assessment of gene correlation balance and validation under extreme conditions

For each of the 33 target genes, Spearman correlation coefficients were calculated against all other selected genes, with genes classified as positively correlated (ρ > 0.5) or negatively correlated (ρ < −0.5). The “correlation balance index” was defined as the average proportion of positively and negatively correlated genes relative to the total strongly correlated genes (|ρ| > 0.5) across all 33 targets. Distribution symmetry was evaluated by the ratio of median absolute values between positive and negative correlations, while selection scale consistency across the 5 methods was assessed using the number of negatively (or positively) correlated genes, including quantity, stacking trends, and overall magnitude. To rigorously test robustness, we selected the top 3 genes exhibiting the largest inter-method discrepancies for in-depth analysis; if TransProPy maintains both overall distribution consistency and its balanced correlation profile under these extreme conditions, it would provide strong evidence for superior generalizability and practical applicability.

### Directional consistency validation and redundancy control in pathway enrichment analysis

Gene set enrichment analysis (GSEA) [25, 26] was performed using KEGG [27–30] and Hallmark [31, 32] gene sets to examine whether the ratio of activated to suppressed pathways at the pathway level maintains directional consistency with the ratio of positively to negatively correlated genes at the gene level. For each target gene under each method, the analysis was repeated 20 times to eliminate stochastic effects, and the proportions of positively and negatively enriched pathways were quantified. Pathways with highly similar gene rankings and overlapping gene compositions (including synonymous, homologous, and sub-pathways) were identified, and both deduplicated and non-deduplicated versions of core-enriched gene statistics were constructed to quantify the impact of pathway redundancy on the robustness of conclusions.

### Quantification of leading-edge genes, identification of super core genes, and intersection-union analysis

Three genes exhibiting the most pronounced correlation differences—CFD, ANKRD35, and ALOXE3—were selected for further analysis. For each gene, the number of leading-edge genes was quantified across both activated and suppressed pathways, with comparisons made between deduplicated and non-deduplicated results. This approach enabled the identification of “super core enriched genes” that recurrently appeared across multiple pathways, along with their amplifying effects on the observed imbalance. Overall comparisons were conducted using the Kruskal–Wallis test (*P* < 0.001), followed by pairwise Wilcoxon tests. Additionally, a Venn network was constructed to visualize the intersection and union of leading-edge genes identified by each method across all significantly enriched pathways, thereby elucidating the sources and contributions of shared core genes versus method-specific complementary findings.

### Cross-dataset generalization evaluation and statistical methods

To validate cross-dataset generalizability, we constructed 12 independent tissue–database combinations [33] spanning diverse cancer types, platforms, and batch sources from multiple public repositories (e.g., GTEx, CPTAC, TCGA, and TARGET). Method scores were computed separately for Hallmark and KEGG gene sets and subsequently aggregated into a composite total score. Results were visualized using 3-layer concentric rings (inner ring: Hallmark; middle ring: KEGG; outer ring: total score), with arc length proportional to the score; zero-score rings were displayed in light gray. The same statistical framework was applied to ratio-based metrics (activation/suppression and positive/negative correlation ratios). For each target gene, 20 iterations per method were performed to assess robustness.

### R package architecture and visualization workflow

TransProR is developed upon R’s core data science and statistical ecosystem, providing a highly modular and extensible analytical framework that consolidates complex multi-step workflows into intuitive, unified function calls. The architecture comprises 4 principal components: (i) a data preprocessing module that automates raw data retrieval, gene identifier conversion, expression matrix normalization, sample classification, and batch-effect correction for reliable multi-source integration; (ii) a DE module offering a unified interface that adaptively selects optimal preprocessing and analytical strategies based on input data characteristics; (iii) a network and pathway analysis module enabling streamlined enrichment analyses through seamless integration with established annotation tools; and (iv) a visualization and utility module supporting adaptive graphical customization, legend rendering, and publication-ready figure generation. The modular design permits both independent and combined use of functional components, with extensive parameter configurations enabling end-to-end customization from data processing to final visual output.

## Discussion

In this framework, classical DE methods were used as baseline approaches for candidate gene screening, whereas TransProPy provided a complementary multivariate framework for refining candidate gene sets. Accordingly, our comparison focuses on how the resulting gene sets differ in downstream correlation patterns, pathway enrichment robustness, and biological interpretation. TransPro establishes an integrated analytical ecosystem for transcriptomic research through 2 complementary packages: TransProPy for robust feature selection and TransProR for comprehensive visualization. Classical DE methods—DESeq2, edgeR, limma, and WRST—are prone to directional selection bias during gene screening; here, this term does not refer to the classical statistical notion of unbiased estimation, but rather to an imbalance that skews the selection of positively and negatively correlated genes. This imbalance may further propagate downstream along the analytical pipeline, manifesting at the pathway level as systematic distortion in the proportions of activated and suppressed pathways driven by method-specific bias. By integrating mvAUC-based complementarity quantification with ensemble-optimized feature selection, TransProPy maintains near-equal proportions of positively and negatively correlated genes even under stringent correlation thresholds (|ρ| > 0.5), and the pathway-level activation–suppression enrichment profile exhibits good concordance with the gene-level correlation distribution; notably, under certain test conditions where other methods failed to yield valid pathway enrichment results, TransProPy consistently completed the analysis. Furthermore, TransProPy identifies both shared core-enriched genes across methods and method-specific signature genes that effectively supplement the analytical results; quantification of super core-enriched genes further reveals that TransProPy alone exhibits bidirectional amplification effects (ratios both >1 and <1), whereas conventional methods tend toward unidirectional bias. Cross-tissue and cross-database validation across 12 independent datasets from GTEx, CPTAC, TCGA, TARGET, and other repositories—encompassing multiple cancer types and normal tissues—demonstrates that TransProPy consistently achieves the highest composite scores in both Hallmark and KEGG enrichment analyses, confirming its broad applicability and robustness.

**Figure 7:**
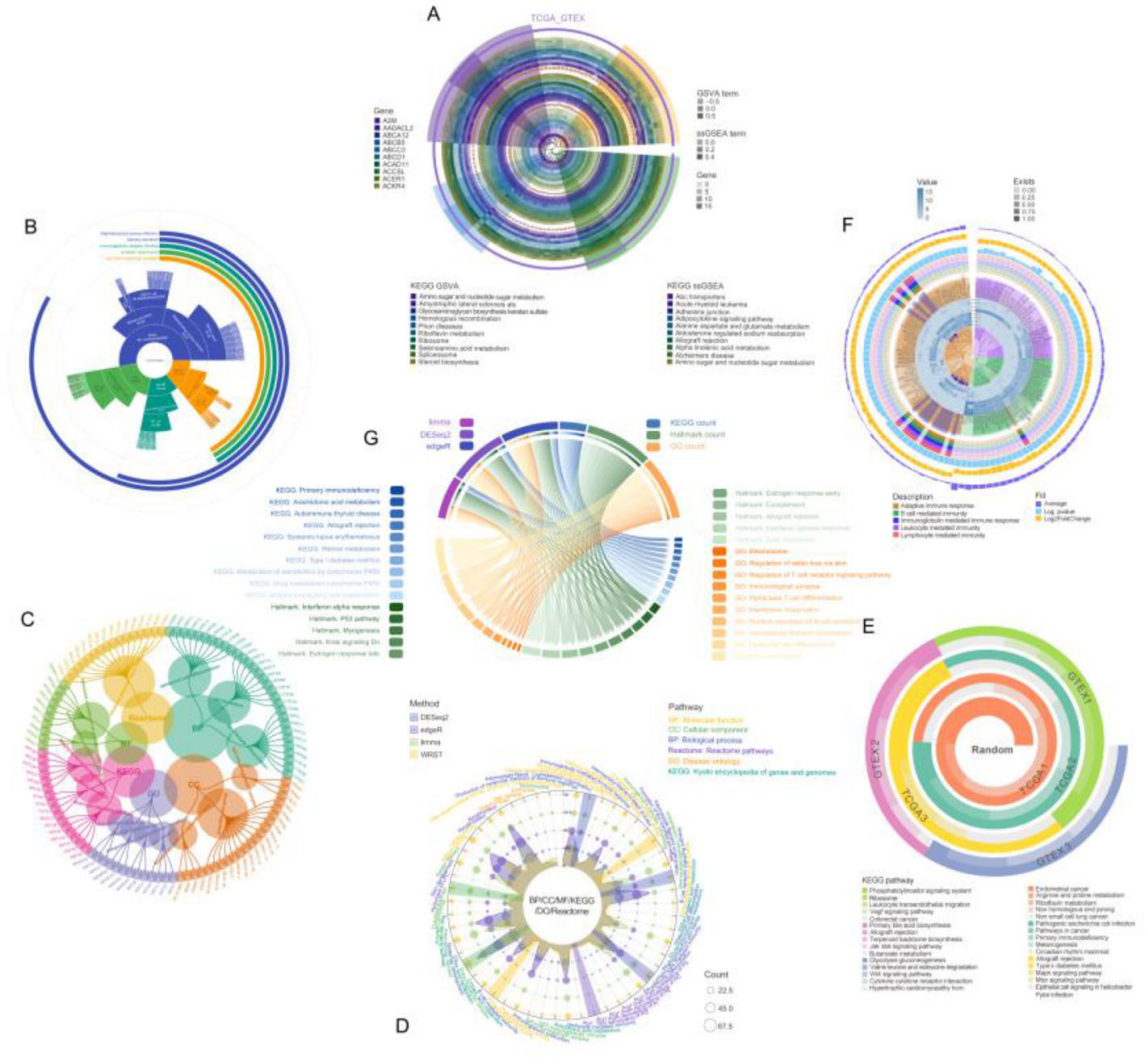
TransProR visualization outputs integrating gene expression patterns, pathway enrichment results, and cross-method comparisons within a single coordinated view. (A) Multi-layer circular plot integrating differential gene expression with pathway enrichment analysis. Concentric rings display gene expression levels (TCGA vs GTEX) and pathway activities (ssGSEA/GSVA), with color gradients indicating expression magnitude and pathway enrichment scores. (B) Circular enrichment plot displaying pathway categories (KEGG, Disease Ontology, GO terms) with radial bar lengths proportional to gene counts. (C) Gene-pathway network diagram showing relationships between differentially expressed genes and biological pathways. Radial organization displays pathway categories (center) extending to associated genes (periphery). (D) Polar bubble plot comparing enrichment results across 4 statistical methods (DESeq2, edgeR, limma, WRST) and 6 pathway databases. Bubble size represents gene count; colored sectors distinguish pathway categories. (E) Spiral heatmap showing pathway activities across TCGA and GTEX sample groups. Color intensity indicates KEGG pathway activity levels. (F) Hierarchical clustering tree with concentric heatmaps displaying gene relationships and multi-dimensional statistical data. Color-coded layers represent fold changes, *P*-values, and expression averages across datasets. (G) Circos plot linking DE methods (limma, DESeq2, edgeR) to pathway resources (KEGG pathways, Hallmark gene sets, and GO terms). Arrow thickness indicates gene contribution; colors distinguish pathway categories.

TransProR complements these analytical capabilities by transforming heterogeneous transcriptomic data into standardized expression matrices through automated preprocessing, batch-effect correction, and multi-method DE analysis. Seven multidimensional visualizations constitute its core outputs: (i) multi-layered radial plots displaying gene expression levels alongside pathway activity scores across datasets (Fig. 7A) [34–36]; (ii) circular bar charts consolidating GO [37] and KEGG [28, 38] enrichment categories within a single compact display (Fig. 7B); (iii) hierarchical network diagrams revealing gene–pathway associations and inter-pathway connectivity (Fig. 7C); (iv) polar bubble plots comparing enrichment consistency across statistical methods, with bubble size proportional to gene counts (Fig. 7D); (v) spiral heatmaps tracking pathway activity dynamics across experimental conditions (Fig. 7E) [39–41]; (vi) integrated circular displays combining clustering dendrograms, expression heatmaps, and enrichment bar charts (Fig. 7F); and (vii) chord diagrams quantifying method-specific contributions to shared pathway enrichment (Fig. 7G) [42].

From a practical standpoint, these combinatorial displays are best suited for cross-method comparison, integration of gene- and pathway-level results, and identification of shared or method-specific biological patterns. By presenting multiple layers of transcriptomic information in coordinated and reproducible visual layouts, they facilitate downstream biological interpretation. Together, TransProPy and TransProR integrate gene selection, enrichment analysis, and result visualization into a unified workflow, thereby improving the standardization, interpretability, and reproducibility of transcriptomic analysis.

## Availability of source code and requirements

Project name: TransProR (v1.0.7) and TransProPy (v1.0.0).

Project home page: TransProR: https://github.com/SSSYDYSSS/TransProR; TransProPy: https://github.com/SSSYDYSSS/TransProPy.

Operating system(s): tested on Linux, macOS, and Windows.

Programming language: R and Python.

Other requirements: R (≥ 4.3.0), Python (≥ 3.9).

License: TransProR: MIT; TransProPy: BSD 3-Clause.

## Supplementary Material

giag057_Supplemental_Files

giag057_Authors_Response_To_Reviewer_Comments_original_submission

giag057_GIGA-D-26-00052_original_submission

giag057_GIGA-D-26-00052_revision_1

giag057_Reviewer_1_Report_original_submission Reviewer 1 -- 3/26/2026

giag057_Reviewer_1_Report_revision_1 Reviewer 1 -- 5/10/2026

giag057_Reviewer_2_Report_original_submission Reviewer 2 -- 3/27/2026

giag057_Reviewer_2_Report_revision_1 Reviewer 2 -- 5/6/2026

giag057_Reviewer_3_Report_original_submission Reviewer 3 -- 3/28/2026

giag057_Reviewer_3_Report_revision_1 Reviewer 3 -- 5/8/2026

## Data Availability

The source code for TransProR (R) and TransProPy (Python) is publicly available. Detailed user documentation is provided in TransProRBook [44] for TransProR and TransProPyBook [45] for TransProPy. The raw data, analysis pipeline, intermediate results, and documentation source files (Quarto) have been deposited in Zenodo [46]. The DOME-ML annotation for this study is available in the DOME-ML Registry [47]. These resources support reproducibility and facilitate the application of the methodologies presented in this study.

## Additional files

**Supplementary Fig. 1:** Ridge plot of correlation distributions for the *CFD* gene. Density distributions of Spearman correlation coefficients (*x*-axis: −1.0 to 1.0) are shown for 5 methods, with separate ridges displayed for negative correlations (left) and positive correlations (right). Vertical dashed lines indicate thresholds at −0.5, 0.5, and 0.0. Methods are labeled on the *y*-axis, with color-coded distributions as indicated in the legend.

**Supplementary Figure 2:** Ridge plot of correlation distributions for the *ANKRD35* gene. Density distributions of Spearman correlation coefficients (*x*-axis: −1.0 to 1.0) are shown for 5 methods, with separate ridges displayed for negative correlations (left) and positive correlations (right). Vertical dashed lines indicate thresholds at −0.5, 0.5, and 0.0. Methods are labeled on the *y*-axis, with color-coded distributions as indicated in the legend.

**Supplementary Figure 3:** Ridge plot of correlation distributions for the *ALOXE3* gene. Density distributions of Spearman correlation coefficients (*x*-axis: −1.0 to 1.0) are shown for 5 methods, with separate ridges displayed for negative correlations (left) and positive correlations (right). Vertical dashed lines indicate thresholds at −0.5, 0.5, and 0.0. Methods are labeled on the *y*-axis, with color-coded distributions as indicated in the legend.

**Supplementary Figure 4:** GSEA enrichment analysis results for the *ANKRD35* gene. GSEA results are shown for 5 methods (DESeq2, edgeR, TransProPy, limma, WRST) using Hallmark and KEGG gene sets. Each method is represented by 3 vertically stacked subplots: the top panel displays the Running Enrichment Score, showing the cumulative enrichment score across the ranked gene list; the middle panel shows the Gene Hit Distribution, indicating the positions of genes within enriched gene sets along the ranked list; and the bottom panel presents the Ranked List Metric, depicting the distribution of the metric used for gene ranking. The *x*-axis uniformly represents “Rank in Ordered Dataset.” The horizontal dashed line (*y* = 0) indicates the no-enrichment baseline. Method names are labeled at the bottom, aligned with their corresponding columns.

**Supplementary Figure 5:** GSEA enrichment analysis results for the *ALOXE3* gene. GSEA results are shown for 5 methods (DESeq2, edgeR, TransProPy, limma, WRST) using Hallmark and KEGG gene sets. Each method is represented by 3 vertically stacked subplots: the top panel displays the Running Enrichment Score, showing the cumulative enrichment score across the ranked gene list; the middle panel shows the Gene Hit Distribution, indicating the positions of genes within enriched gene sets along the ranked list; and the bottom panel presents the Ranked List Metric, depicting the distribution of the metric used for gene ranking. The *x*-axis uniformly represents “Rank in Ordered Dataset.” The horizontal dashed line (*y* = 0) indicates the no-enrichment baseline. Method names are labeled at the bottom, aligned with their corresponding columns.

**Supplementary Figure 6:** Pathway proportion distribution across 20 repeated GSEA analyses for 33 target genes using the DESeq2 method. A total of 33 subplots are displayed, each corresponding to one target gene. The *x*-axis represents the repetition number (Index, 1–20), and the *y*-axis indicates the proportion of activated pathways among activated and suppressed pathways (Value, 0.00–1.00). Red lines (with circular markers) represent results from the Hallmark gene sets, while blue lines (with circular markers) represent results from the KEGG gene set. Blue and red vertical lines indicate cases where no pathways were enriched in KEGG and Hallmarks, respectively (NaN values). Each subplot is titled with the corresponding target gene name.

**Supplementary Figure 7:** Pathway proportion distribution across 20 repeated GSEA analyses for 33 target genes using the edgeR method. A total of 33 subplots are displayed, each corresponding to one target gene. The *x*-axis represents the repetition number (Index, 1–20), and the *y*-axis indicates the proportion of activated pathways among activated and suppressed pathways (Value, 0.00–1.00). Red lines (with circular markers) represent results from the Hallmark gene sets, while blue lines (with circular markers) represent results from the KEGG gene set. Blue and red vertical lines indicate cases where no pathways were enriched in KEGG and Hallmarks, respectively (NaN values). Each subplot is titled with the corresponding target gene name.

**Supplementary Figure 8:** Pathway proportion distribution across 20 repeated GSEA analyses for 33 target genes using the limma method. A total of 33 subplots are displayed, each corresponding to one target gene. The *x*-axis represents the repetition number (Index, 1–20), and the *y*-axis indicates the proportion of activated pathways among activated and suppressed pathways (Value, 0.00–1.00). Red lines (with circular markers) represent results from the Hallmark gene sets, while blue lines (with circular markers) represent results from the KEGG gene set. Blue and red vertical lines indicate cases where no pathways were enriched in KEGG and Hallmarks, respectively (NaN values). Each subplot is titled with the corresponding target gene name.

**Supplementary Figure 9:** Pathway proportion distribution across 20 repeated GSEA analyses for 33 target genes using the WRST method. A total of 33 subplots are displayed, each corresponding to one target gene. The *x*-axis represents the repetition number (Index, 1–20), and the *y*-axis indicates the proportion of activated pathways among activated and suppressed pathways (Value, 0.00–1.00). Red lines (with circular markers) represent results from the Hallmark gene sets, while blue lines (with circular markers) represent results from the KEGG gene set. Blue and red vertical lines indicate cases where no pathways were enriched in KEGG and Hallmarks, respectively (NaN values). Each subplot is titled with the corresponding target gene name.

**Supplementary Figure 10:** Pathway proportion distribution across 20 repeated GSEA analyses for 33 target genes using the TransproPy method. A total of 33 subplots are displayed, each corresponding to one target gene. The *x*-axis represents the repetition number (Index, 1–20), and the *y*-axis indicates the proportion of activated pathways among activated and suppressed pathways (Value, 0.00–1.00). Red lines (with circular markers) represent results from the Hallmark gene sets, while blue lines (with circular markers) represent results from the KEGG gene set. Blue and red vertical lines indicate cases where no pathways were enriched in KEGG and Hallmarks, respectively (NaN values). Each subplot is titled with the corresponding target gene name.

## References

[1] Wang Z, Gerstein M, Snyder M. RNA-seq: a revolutionary tool for transcriptomics. Nat Rev Genet. 2009;10:57–63. 10.1038/nrg2484. 19015660 PMC2949280

[2] Argelaguet R, Cuomo ASE, Stegle O, et al. Computational principles and challenges in single-cell data integration. Nat Biotechnol. 2021;39:1202–15. 10.1038/s41587-021-00895-7. 33941931

[3] Cao J, O’Day DR, Pliner HA et al. A human cell atlas of fetal gene expression. Science. 2020;370:eaba7721. 10.1126/science.aba7721. 33184181 PMC7780123

[4] Argelaguet R, Clark SJ, Mohammed H, et al. Multi-omics profiling of mouse gastrulation at single-cell resolution. Nature. 2019;576:487–91. 10.1038/s41586-019-1825-8. 31827285 PMC6924995

[5] Allison DB, Cui X, Page GP, et al. Microarray data analysis: from disarray to consolidation and consensus. Nat Rev Genet. 2006;7:55–65. 10.1038/nrg1749. 16369572

[6] Love MI, Huber W, Anders S. Moderated estimation of fold change and dispersion for RNA-seq data with DESeq2. Genome Biol. 2014;15:550. 10.1186/s13059-014-0550-8. 25516281 PMC4302049

[7] Robinson MD, McCarthy DJ, Smyth GK. edgeR: a bioconductor package for differential expression analysis of digital gene expression data. Bioinformatics. 2010;26:139–40. 10.1093/bioinformatics/btp616. 19910308 PMC2796818

[8] Ritchie ME, Phipson B, Wu D, et al. limma powers differential expression analyses for RNA-sequencing and microarray studies. Nucleic Acids Res. 2015;43:e47. 10.1093/nar/gkv007. 25605792 PMC4402510

[9] Smyth GK . limma: Linear Models for Microarray Data. New York, NY: Springer; 2005: 397–420.

[10] Li Y, Ge X, Peng F, et al. Exaggerated false positives by popular differential expression methods when analyzing human population samples. Genome Biol. 2022;23:79. 10.1186/s13059-022-02648-4. 35292087 PMC8922736

[11] Su Y, Du K, Wang J, et al. Multi-variable AUC for sifting complementary features and its biomedical application. Brief Bioinform. 2022;23:bbac029. 10.1093/bib/bbac029. 35212712

[12] Chen X-W, Wasikowski M. FAST: A Roc-Based Feature Selection Metric for Small Samples and Imbalanced Data Classification Problems. New York, NY: ACM; 2008: 124–32.

[13] Wang R, Tang K. Feature Selection for Maximizing the Area under the ROC Curve. Piscataway, NJ: IEEE; 2009. 10.1109/ICDMW.2009.25.

[14] Sun L, Wang J, Wei J. AVC: selecting discriminative features on basis of AUC by maximizing variable complementarity. BMC Bioinf. 2017;18:50. 10.1186/s12859-017-1468-4. PMC5374660 28361689

[15] Robnik-Šikonja M, Kononenko I. Theoretical and empirical analysis of ReliefF and RReliefF. Machine Learning. 2003;53:23–69. 10.1023/a:1025667309714.

[16] Hanchuan P, Fuhui L, Ding C. Feature selection based on mutual information criteria of max-dependency, max-relevance, and min-redundancy. IEEE Trans Pattern Anal Mach Intell. 2005;27:1226–38. 10.1109/tpami.2005.159. 16119262

[17] Wang J, Wei J-M, Yang Z, et al. Feature selection by maximizing independent classification information. IEEE Trans Knowl Data Eng. 2017;29:828–41. 10.1109/tkde.2017.2650906.

[18] Tibshirani R, Hastie T, Narasimhan B, et al. Diagnosis of multiple cancer types by shrunken centroids of gene expression. Proc Natl Acad Sci USA. 2002;99:6567–72. 10.1073/pnas.082099299. 12011421 PMC124443

[19] Weinstein JN, Collisson EA, Mills GB, et al. The cancer genome atlas pan-cancer analysis project. Nat Genet. 2013;45:1113–20. 10.1038/ng.2764. 24071849 PMC3919969

[20] Erickson N, Mueller J, Shirkov A, et al. AutoGluon-tabular: robust and accurate AutoML for structured data. 7th ICML Workshop on Automated Machine Learning; 2020; 10.48550/arxiv.2003.06505.

[21] Kotthoff L, Thornton C, Hoos HH, et al. Auto-WEKA: Automatic Model Selection and Hyperparameter Optimization in WEKA. Cham: Springer International Publishing; 2019: 81–95.

[22] Thornton C, Hutter F, Hoos HH, et al. Auto-WEKA: Combined Selection and Hyperparameter Optimization of Classification Algorithms. New York, NY: ACM; 2013. 10.1145/2487575.2487629.

[23] Feurer M, Springenberg JT, Hutter F. Using meta-learning to initialize bayesian optimization of hyperparameters. In: Mlas'14. Aachen: CEUR-WS.org; 2014: 3–10.

[24] Lonsdale J, Thomas J, Salvatore M, et al. The Genotype-Tissue Expression (GTEx) project. Nat Genet. 2013;45:580–85. 10.1038/ng.2653. 23715323 PMC4010069

[25] Subramanian A, Tamayo P, Mootha VK, et al. Gene set enrichment analysis: a knowledge-based approach for interpreting genome-wide expression profiles. Proc Natl Acad Sci USA. 2005;102:15545–50. 10.1073/pnas.0506580102. 16199517 PMC1239896

[26] Reimand J, Isserlin R, Voisin V, et al. Pathway enrichment analysis and visualization of omics data using g:profiler, GSEA, Cytoscape and EnrichmentMap. Nat Protoc. 2019;14:482–517. 10.1038/s41596-018-0103-9. 30664679 PMC6607905

[27] Kanehisa M . Toward understanding the origin and evolution of cellular organisms. Protein Sci. 2019;28:1947–51. 10.1002/pro.3715. 31441146 PMC6798127

[28] Kanehisa M, Furumichi M, Tanabe M, et al. KEGG: new perspectives on genomes, pathways, diseases and drugs. Nucleic Acids Res. 2017;45:D353–61. 10.1093/nar/gkw1092. 27899662 PMC5210567

[29] Kanehisa M, Goto S, Sato Y, et al. KEGG for integration and interpretation of large-scale molecular data sets. Nucleic Acids Res. 2012;40:D109–14. 10.1093/nar/gkr988. 22080510 PMC3245020

[30] Kanehisa M . KEGG: Kyoto Encyclopedia of Genes and Genomes. Nucleic Acids Res. 2000;28:27–30. 10.1093/nar/28.1.27. 10592173 PMC102409

[31] Liberzon A, Subramanian A, Pinchback R, et al. Molecular signatures database (MSigDB) 3.0. Bioinformatics. 2011;27:1739–40. 10.1093/bioinformatics/btr260. 21546393 PMC3106198

[32] Liberzon A, Birger C, Thorvaldsdóttir H, et al. The Molecular Signatures Database hallmark gene set collection. Cell Syst. 2015;1:417–25. 10.1016/j.cels.2015.12.004. 26771021 PMC4707969

[33] National Cancer Institute . Genomic data commons data portal. https://portal.gdc.cancer.gov. Accessed 21 May 2026.

[34] Yu G, Lam TT-Y, Zhu H, et al. Two methods for mapping and visualizing associated data on phylogeny using Ggtree. Mol Biol Evol. 2018;35:3041–43. 10.1093/molbev/msy194. 30351396 PMC6278858

[35] Xu S, Dai Z, Guo P, et al. ggtreeExtra: compact visualization of richly annotated phylogenetic data. Mol Biol Evol. 2021;38:4039–42. 10.1093/molbev/msab166. 34097064 PMC8382893

[36] Xu S, Li L, Luo X, et al. Ggtree: a serialized data object for visualization of a phylogenetic tree and annotation data. iMeta. 2022;1:e56. 10.1002/imt2.56. 38867905 PMC10989815

[37] Consortium TGO . Expansion of the gene ontology knowledgebase and resources. Nucleic Acids Res. 2017;45:D331–8. 10.1093/nar/gkw1108. 27899567 PMC5210579

[38] Kanehisa M, Furumichi M, Sato Y, et al. KEGG for taxonomy-based analysis of pathways and genomes. Nucleic Acids Res. 2023;51:D587–92. 10.1093/nar/gkac963. 36300620 PMC9825424

[39] Barbie DA, Tamayo P, Boehm JS, et al. Systematic RNA interference reveals that oncogenic KRAS-driven cancers require TBK1. Nature. 2009;462:108–12. 10.1038/nature08460. 19847166 PMC2783335

[40] Hänzelmann S, Castelo R, Guinney J. GSVA: gene set variation analysis for microarray and RNA-seq data. BMC Bioinf. 2013;14:7. 10.1186/1471-2105-14-7. PMC3618321 23323831

[41] Gu Z, Hübschmann D. spiralize: an R package for visualizing data on spirals. Bioinformatics. 2022;38:1434–36. 10.1093/bioinformatics/btab778. 34849585 PMC8826351

[42] Gu Z, Gu L, Eils R, et al. Circlize implements and enhances circular visualization in R. Bioinformatics. 2014;30:2811–12. 10.1093/bioinformatics/btu393. 24930139

[43] OpenAI . https://chat.openai.com. Accessed 25 January 2026.

[44] TransProRBook . https://sssydysss.github.io/TransProRBook/. Accessed 30 January 2026.

[45] TransProPyBook . https://sssydysss.github.io/TransProPyBook/. Accessed 30 January 2026.

[46] Complete Initial Data for TransProPyBook and TransProRBook: Full Reproduction Using Scripts from Both Manuals. https://zenodo.org/records/14561230. Accessed 30 January 2026.

[47] Yu D, Li C, Yan S, et al. Comparative evaluation of gene selection approaches in transcriptomics: bias correction and visualization with TransPro. [DOME-ML Annotation]. https://registry.dome-ml.org/review/zszyrfeu0t, 2026. Accessed 13 May 2026. 10.1093/gigascience/giag057 PMC13215095 42148814

