# Supplementary material for: Comparative evaluation of gene selection approaches in transcriptomics: bias correction and visualization with TransPro
Source: Gigascience. 2026 May 18;15:giag057. doi: 10.1093/gigascience/giag057 (PMC13215095; doi:10.1093/gigascience/giag057)
Supplement: giag057_Supplemental_Files [file giag057_supplemental_files.docx]

**Supplementary Information for**

**Comparative Evaluation of Gene Selection Approaches in Transcriptomics: Bias Correction and Visualization with TransPro**

Dongyue Yu^1^, Chen Li^2^, Shuo Yan^3^, Lujiale Guo^4^, Jingyu Liang^1^, Shengquan Chen^5,^* and Wenjun Bu^1,^*

^1^Institute of Entomology, College of Life Sciences, Nankai University, Tianjin 300071, China

^2^Tianjin Medical University Cancer Institute and Hospital, Tianjin 300071, China

^3^AI Thrust, The Hong Kong University of Science and Technology (Guangzhou), Guangzhou 510000, China

^4^ Zhongshan Hospital of Fudan University, Shanghai 200032, China

^5^School of Mathematical Sciences and LPMC, Nankai University, Tianjin 300071, China

*Corresponding authors.


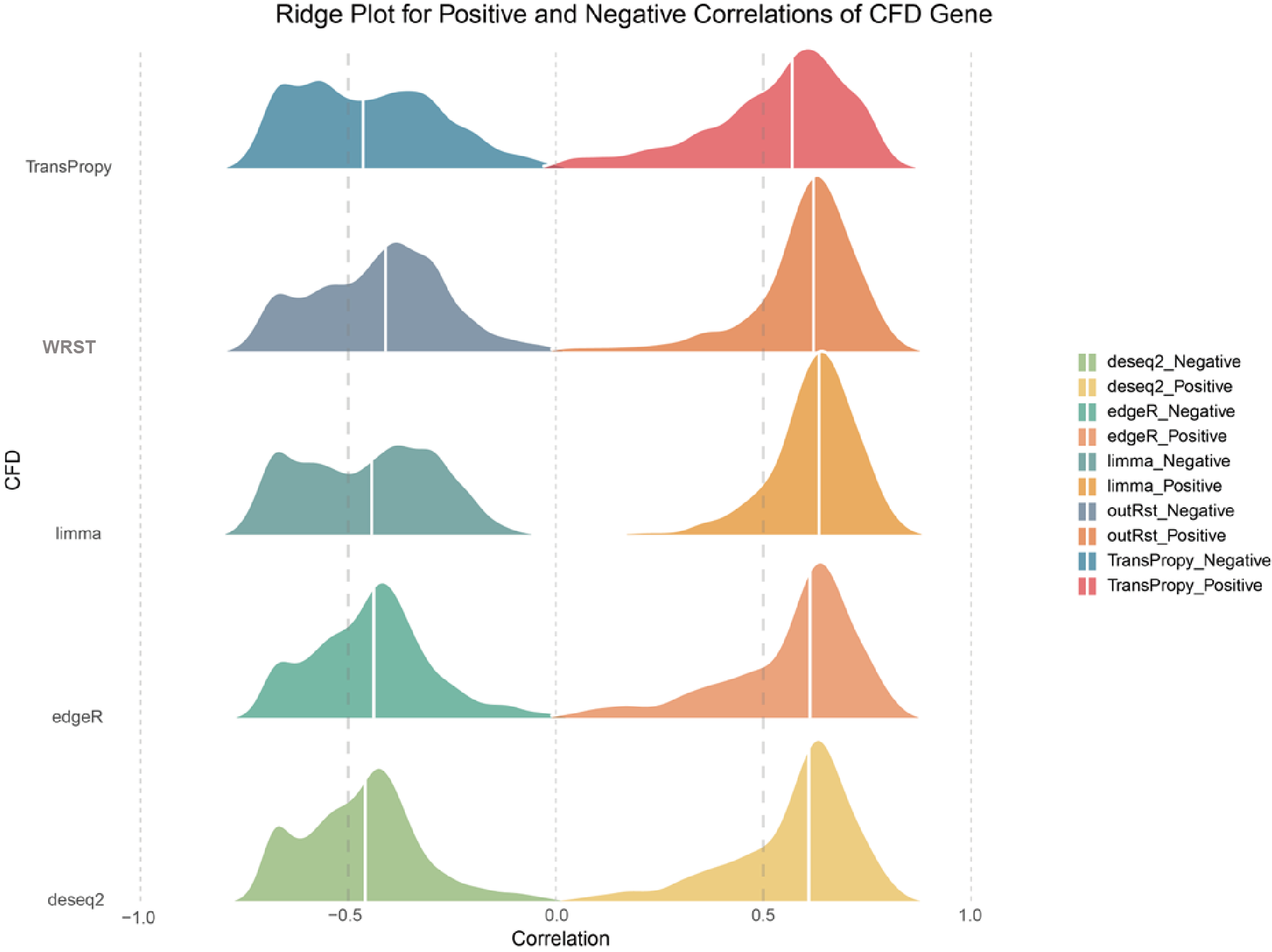


**Supplementary Figure 1. Ridge plot of correlation distributions for the *CFD* gene.** Density distributions of Spearman correlation coefficients (x-axis: −1.0 to 1.0) are shown for five methods, with separate ridges displayed for negative correlations (left) and positive correlations (right). Vertical dashed lines indicate thresholds at −0.5, 0.5, and 0.0. Methods are labeled on the y-axis, with color-coded distributions as indicated in the legend.


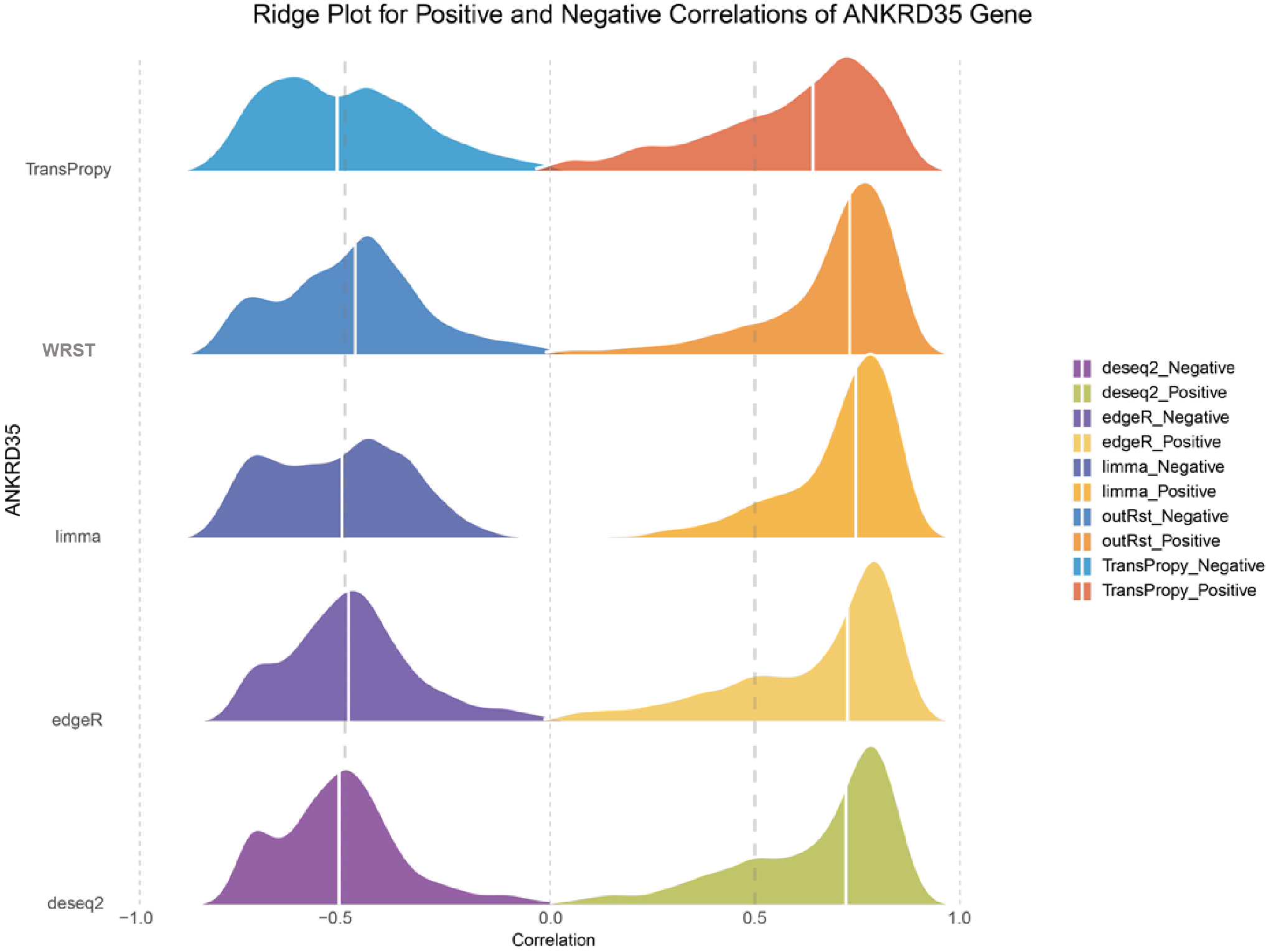


**Supplementary Figure 2. Ridge plot of correlation distributions for the *ANKRD35* gene.** Density distributions of Spearman correlation coefficients (x-axis: −1.0 to 1.0) are shown for five methods, with separate ridges displayed for negative correlations (left) and positive correlations (right). Vertical dashed lines indicate thresholds at −0.5, 0.5, and 0.0. Methods are labeled on the y-axis, with color-coded distributions as indicated in the legend.


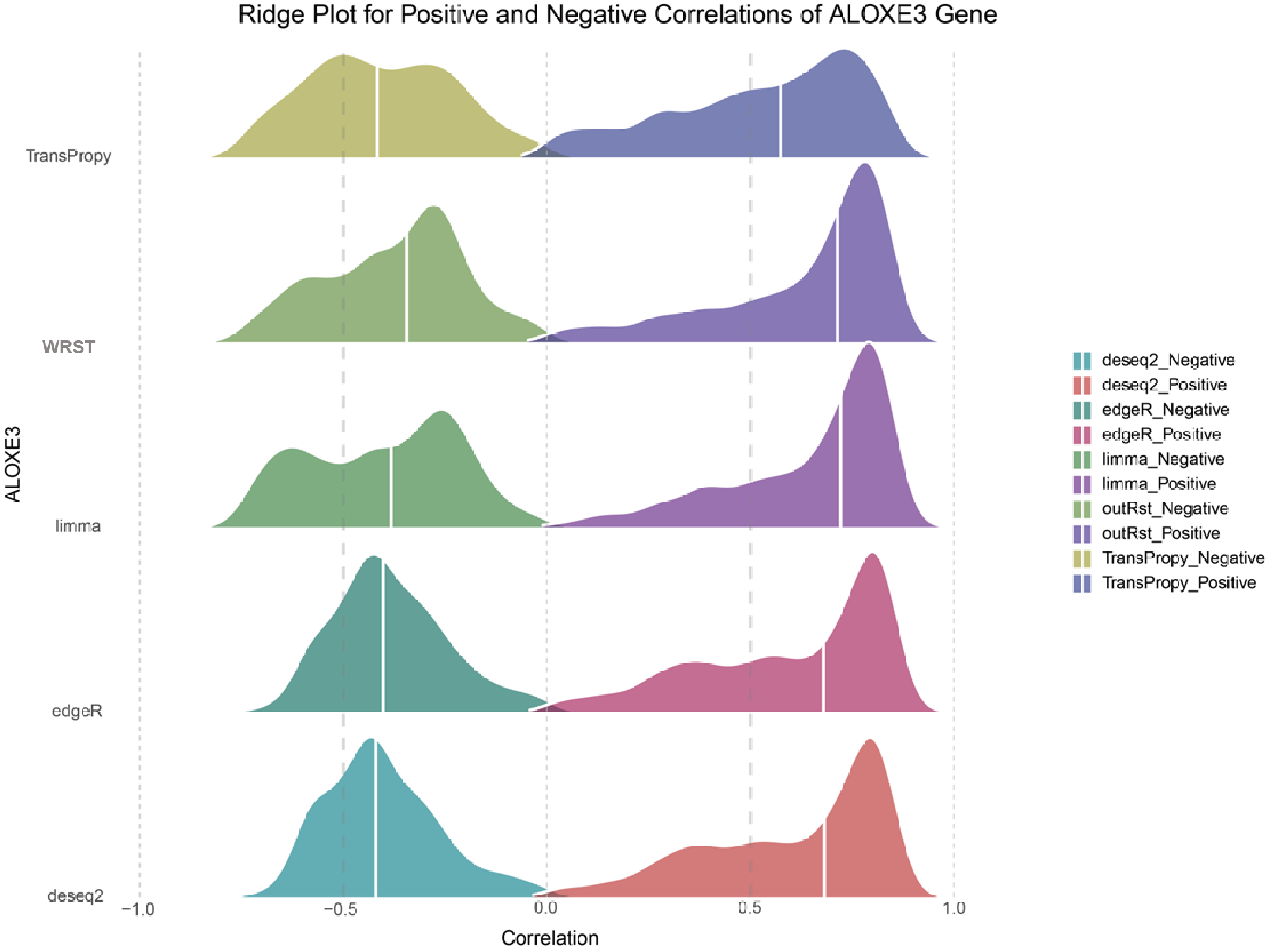


**Supplementary Figure 3. Ridge plot of correlation distributions for the *ALOXE3* gene.** Density distributions of Spearman correlation coefficients (x-axis: −1.0 to 1.0) are shown for five methods, with separate ridges displayed for negative correlations (left) and positive correlations (right). Vertical dashed lines indicate thresholds at −0.5, 0.5, and 0.0. Methods are labeled on the y-axis, with color-coded distributions as indicated in the legend.


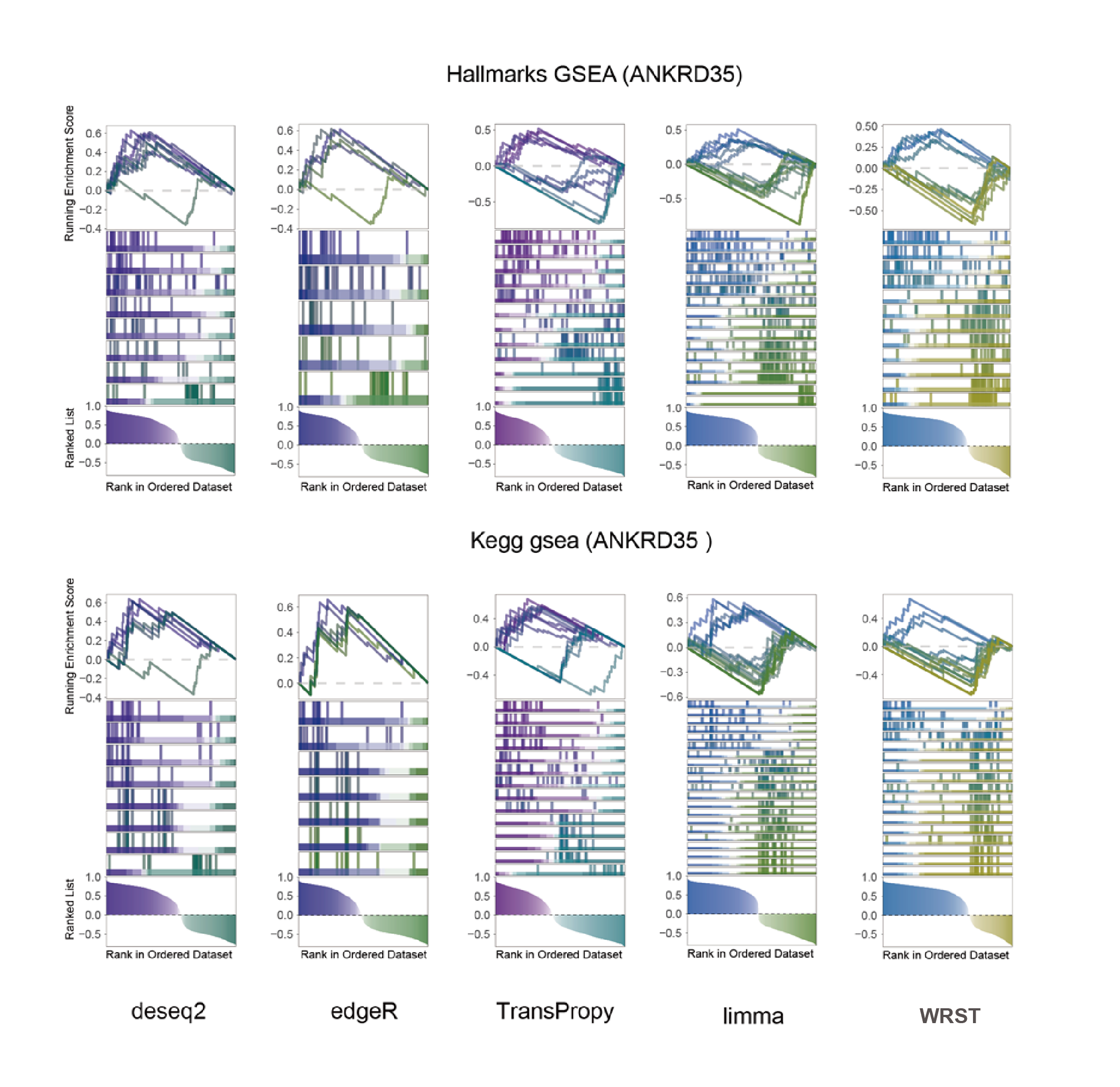


**Supplementary Figure 4. GSEA enrichment analysis results for the *ANKRD35* gene.** GSEA results are shown for five methods (DESeq2, edgeR, TransProPy, limma, WRST) using Hallmark and KEGG gene sets. Each method is represented by three vertically stacked subplots: the top panel displays the Running Enrichment Score, showing the cumulative enrichment score across the ranked gene list; the middle panel shows the Gene Hit Distribution, indicating the positions of genes within enriched gene sets along the ranked list; and the bottom panel presents the Ranked List Metric, depicting the distribution of the metric used for gene ranking. The x-axis uniformly represents "Rank in Ordered Dataset." The horizontal dashed line (y = 0) indicates the no-enrichment baseline. Method names are labeled at the bottom, aligned with their corresponding columns.


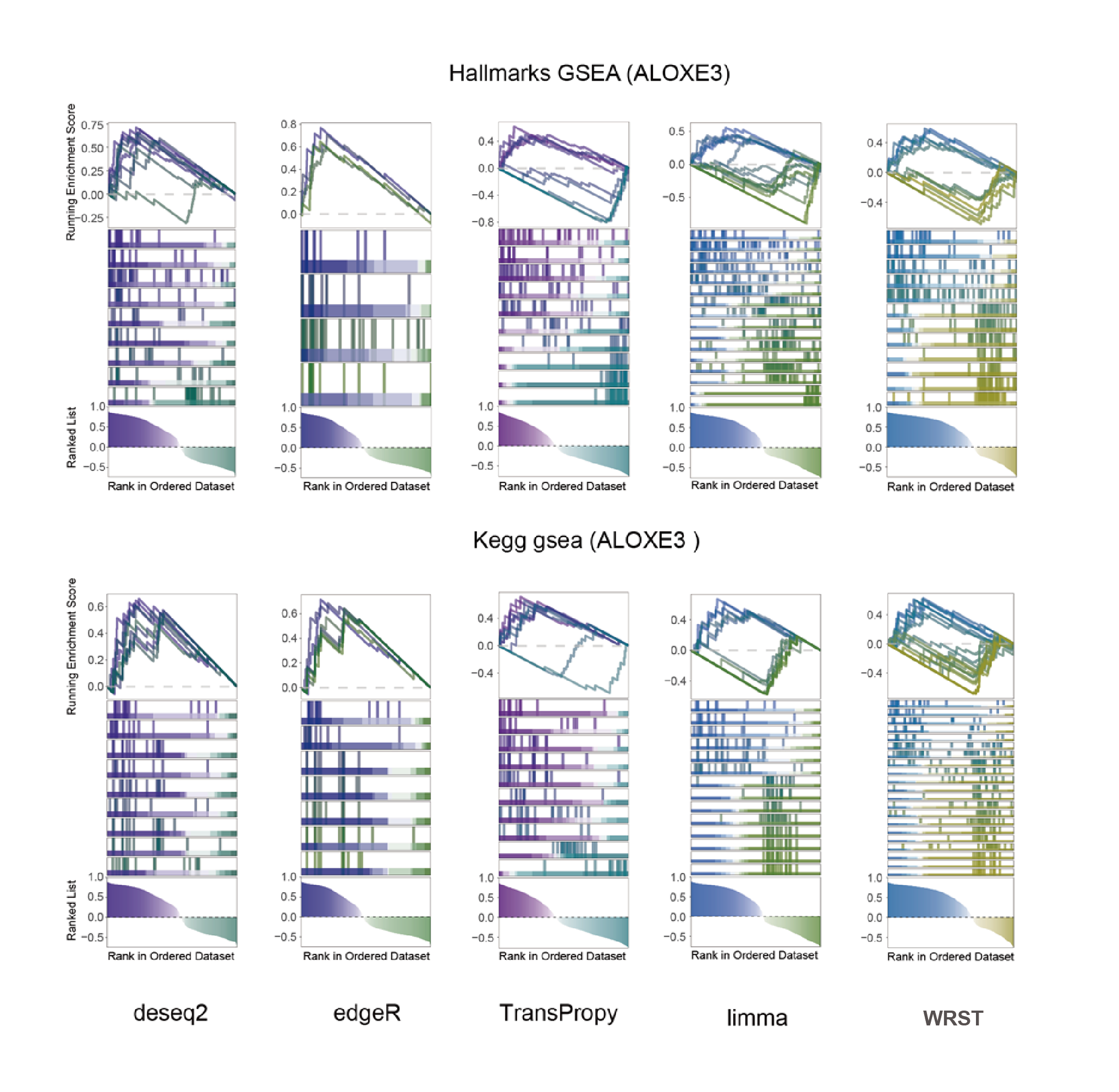


**Supplementary Figure 5. GSEA enrichment analysis results for the *ALOXE3* gene.** GSEA results are shown for five methods (DESeq2, edgeR, TransProPy, limma, WRST) using Hallmark and KEGG gene sets. Each method is represented by three vertically stacked subplots: the top panel displays the Running Enrichment Score, showing the cumulative enrichment score across the ranked gene list; the middle panel shows the Gene Hit Distribution, indicating the positions of genes within enriched gene sets along the ranked list; and the bottom panel presents the Ranked List Metric, depicting the distribution of the metric used for gene ranking. The x-axis uniformly represents "Rank in Ordered Dataset." The horizontal dashed line (y = 0) indicates the no-enrichment baseline. Method names are labeled at the bottom, aligned with their corresponding columns.


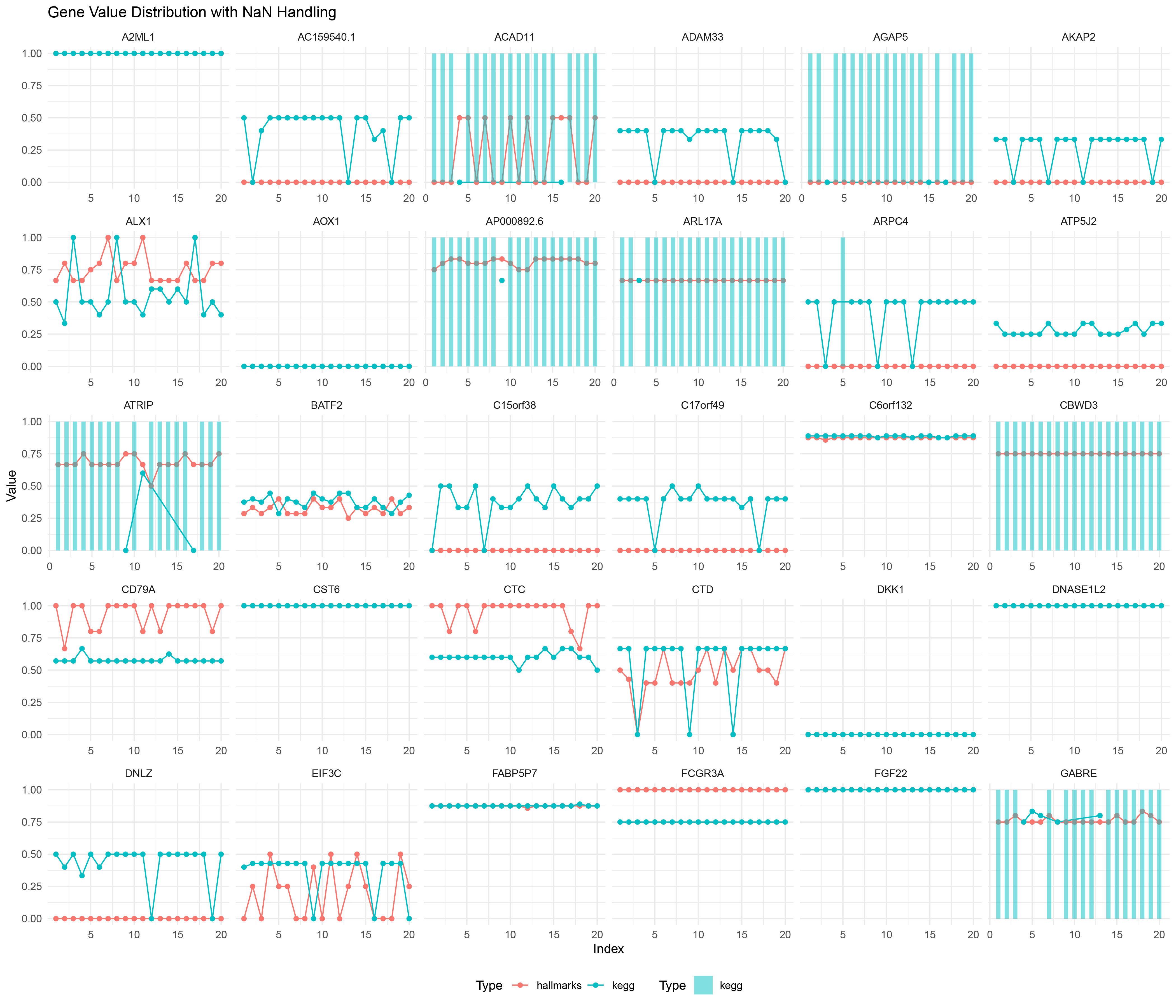


**Supplementary Figure 6. Pathway proportion distribution across 20 repeated GSEA analyses for 33 target genes using the DESeq2 method.** A total of 33 subplots are displayed, each corresponding to one target gene. The x-axis represents the repetition number (Index, 1–20), and the y-axis indicates the proportion of activated pathways among activated and suppressed pathways (Value, 0.00–1.00). Red lines (with circular markers) represent results from the Hallmark gene sets, while blue lines (with circular markers) represent results from the KEGG gene set. Blue and red vertical lines indicate cases where no pathways were enriched in KEGG and Hallmarks, respectively (NaN values). Each subplot is titled with the corresponding target gene name.


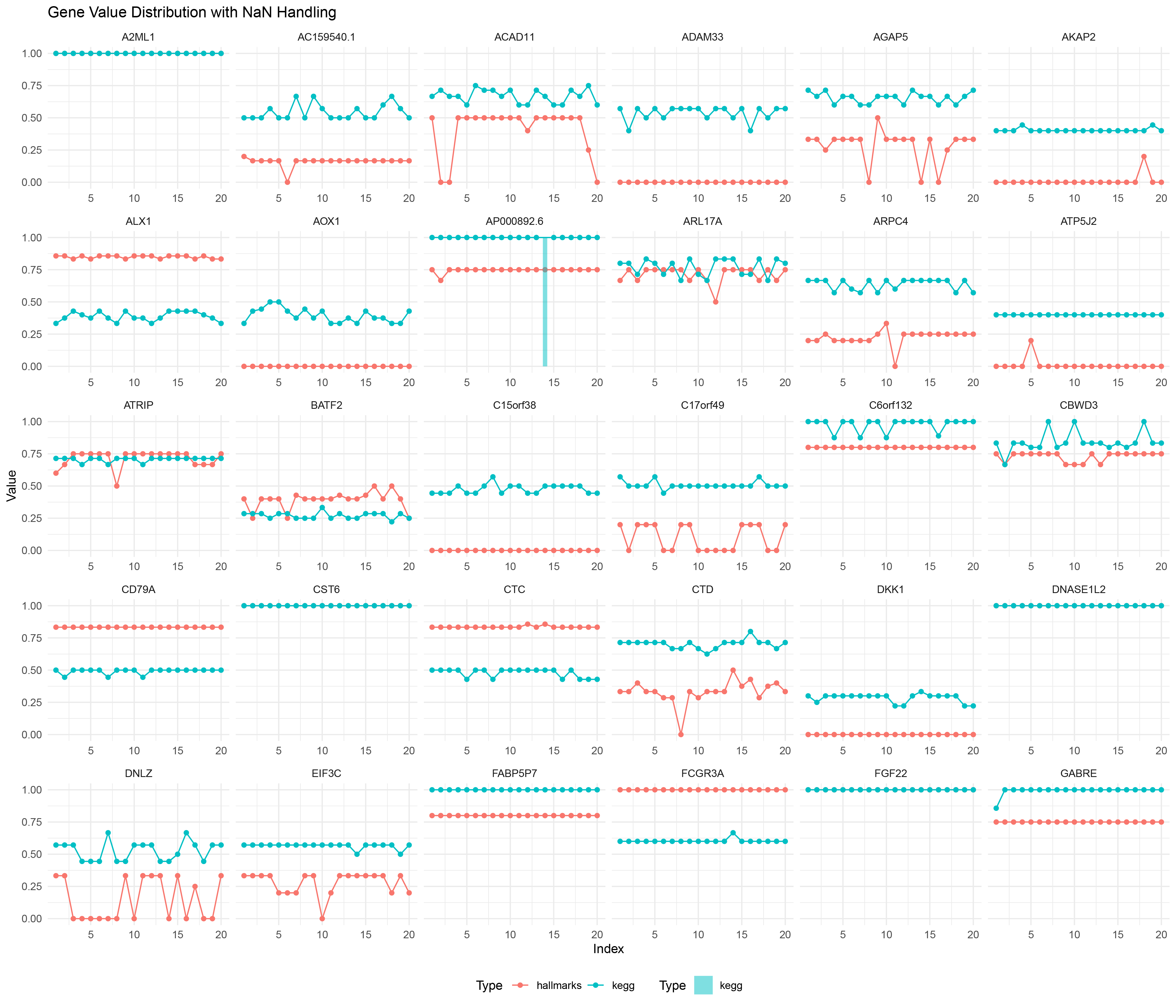


**Supplementary Figure 7. Pathway proportion distribution across 20 repeated GSEA analyses for 33 target genes using the edgeR method.** A total of 33 subplots are displayed, each corresponding to one target gene. The x-axis represents the repetition number (Index, 1–20), and the y-axis indicates the proportion of activated pathways among activated and suppressed pathways (Value, 0.00–1.00). Red lines (with circular markers) represent results from the Hallmark gene sets, while blue lines (with circular markers) represent results from the KEGG gene set. Blue and red vertical lines indicate cases where no pathways were enriched in KEGG and Hallmarks, respectively (NaN values). Each subplot is titled with the corresponding target gene name.


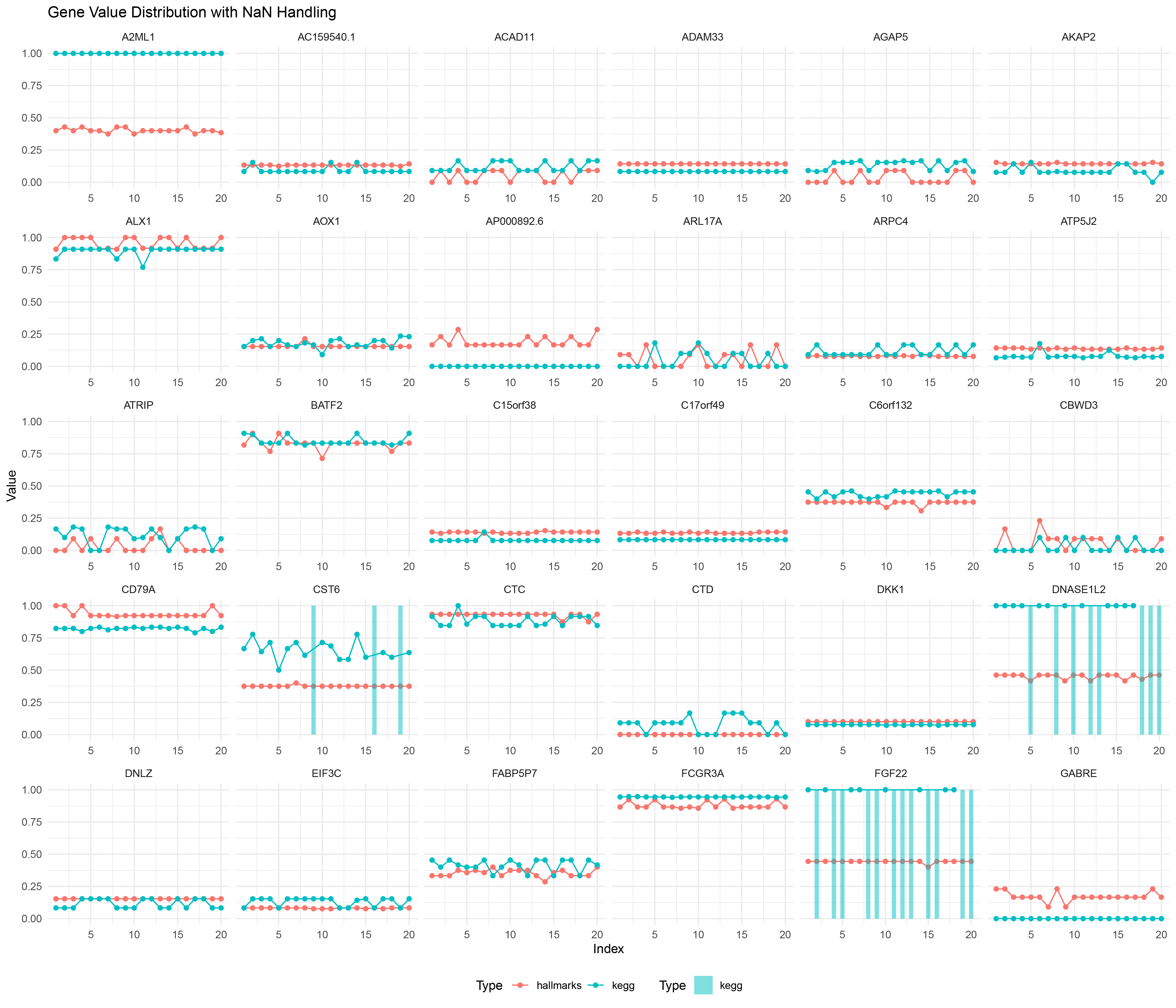


**Supplementary Figure 8. Pathway proportion distribution across 20 repeated GSEA analyses for 33 target genes using the limma method.** A total of 33 subplots are displayed, each corresponding to one target gene. The x-axis represents the repetition number (Index, 1–20), and the y-axis indicates the proportion of activated pathways among activated and suppressed pathways (Value, 0.00–1.00). Red lines (with circular markers) represent results from the Hallmark gene sets, while blue lines (with circular markers) represent results from the KEGG gene set. Blue and red vertical lines indicate cases where no pathways were enriched in KEGG and Hallmarks, respectively (NaN values). Each subplot is titled with the corresponding target gene name.


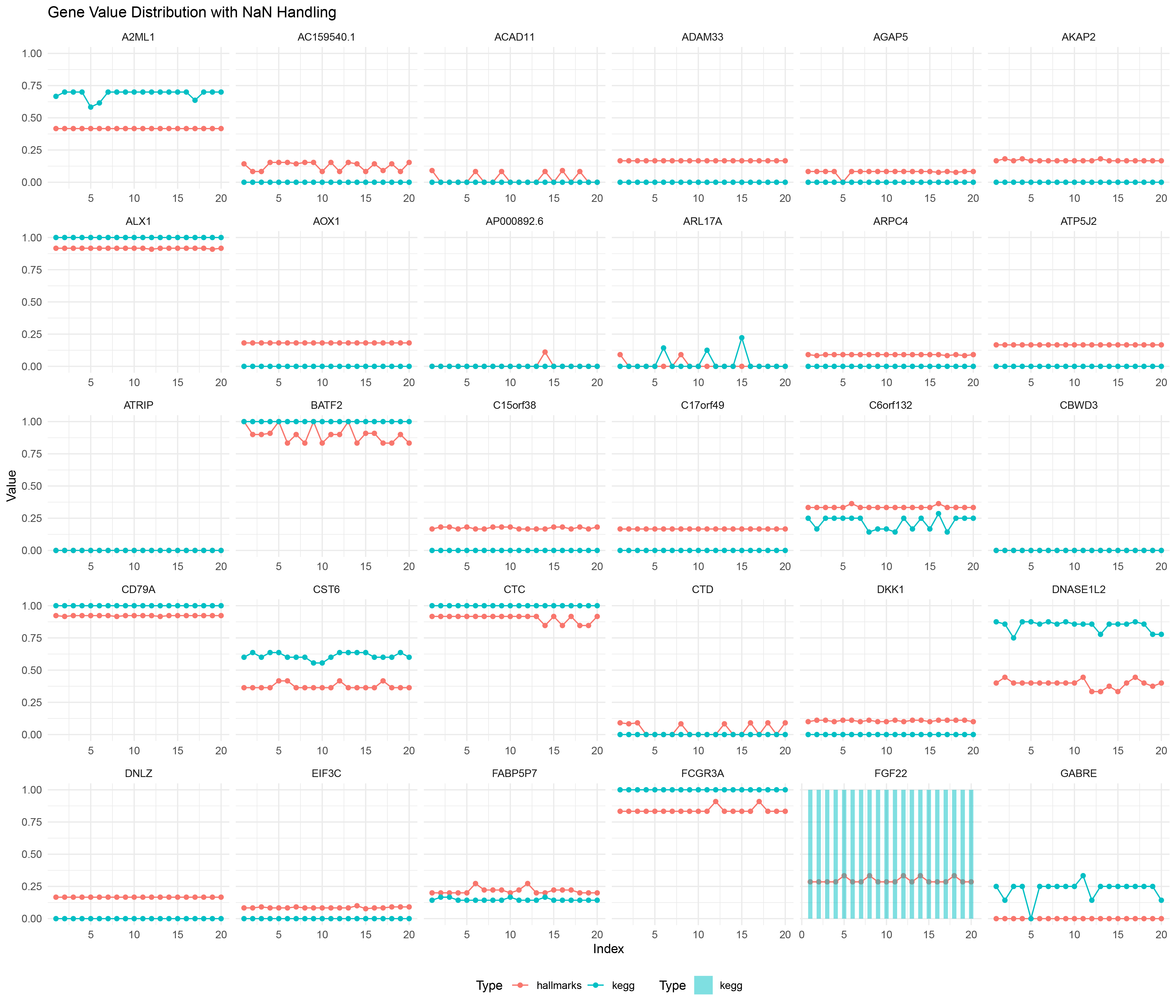


**Supplementary Figure 9. Pathway proportion distribution across 20 repeated GSEA analyses for 33 target genes using the WRST method.** A total of 33 subplots are displayed, each corresponding to one target gene. The x-axis represents the repetition number (Index, 1–20), and the y-axis indicates the proportion of activated pathways among activated and suppressed pathways (Value, 0.00–1.00). Red lines (with circular markers) represent results from the Hallmark gene sets, while blue lines (with circular markers) represent results from the KEGG gene set. Blue and red vertical lines indicate cases where no pathways were enriched in KEGG and Hallmarks, respectively (NaN values). Each subplot is titled with the corresponding target gene name.


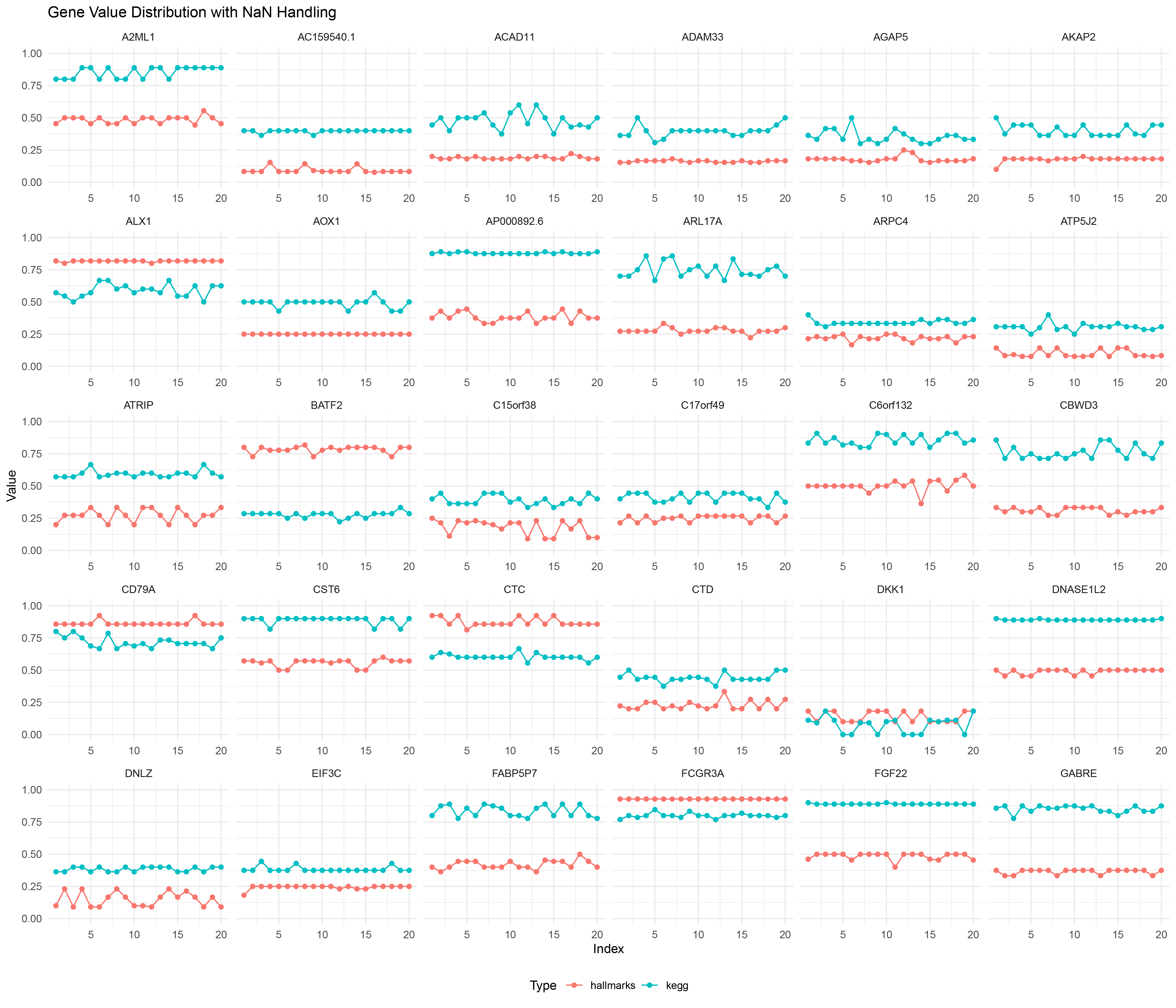


**Supplementary Figure 10. Pathway proportion distribution across 20 repeated GSEA analyses for 33 target genes using the TransproPy method.** A total of 33 subplots are displayed, each corresponding to one target gene. The x-axis represents the repetition number (Index, 1–20), and the y-axis indicates the proportion of activated pathways among activated and suppressed pathways (Value, 0.00–1.00). Red lines (with circular markers) represent results from the Hallmark gene sets, while blue lines (with circular markers) represent results from the KEGG gene set. Blue and red vertical lines indicate cases where no pathways were enriched in KEGG and Hallmarks, respectively (NaN values). Each subplot is titled with the corresponding target gene name.

**Supplementary Note 1**

**Formal definition of the Figure 5 scoring system**

To improve the transparency and reproducibility of the cross-dataset evaluation shown in Figure 4d, we provide here the formal definition of the Hallmark score, KEGG score, and Total score.

For each gene $g$, method $m$, pathway database $t$ (Hallmark or KEGG), and repeated enrichment run $r$, let $P_{g,m,t,r}$ denote the number of positively enriched pathways (NES $>0$) and $N_{g,m,t,r}$ denote the number of negatively enriched pathways (NES $\left< 0 \right.$). The positive-enrichment proportion is defined as

$$R_{g,m,t,r}=\frac{P_{g,m,t,r}}{P_{g,m,t,r}+N_{g,m,t,r}}.$$

If $P_{g,m,t,r}+N_{g,m,t,r}=0$, the corresponding ratio is treated as non-computable and recorded as NaN.

For each $(g, m, t)$, repeated-run values are aggregated over valid runs to obtain a method-specific gene-level value:

$$R_{g,m,t}=\mathrm{mean}\{R_{g,m,t,r}:R_{g,m,t,r}\text{ is computable}\}.$$

That is, the mean is calculated only over computable repeated-run values, whereas non-computable entries are excluded from the aggregation.

To evaluate pathway-level balance, we define the distance from the balanced reference value 0.5 as:

$$d_{g,m,t}=\mid R_{g,m,t}-0.5\mid.$$

For each gene within each pathway database, the method(s) with the minimum distance receive +1 point (ties allowed), reflecting the closest agreement with balanced activation–suppression behavior. Methods with non-computable aggregated values (NaN) receive a −1 penalty. Genes with insufficient computable methods, or genes for which all computable methods yield the same distance, are excluded from scoring in that database.

The database-specific scores are then calculated by summing gene-level contributions across all retained genes:

$$\mathrm{HallmarkScore}_{m}=\sum_{g\in G_{H}} s_{g,m,\mathrm{Hallmark}},$$

$$\mathrm{KEGGScore}_{m}=\sum_{g\in G_{K}} s_{g,m,\mathrm{KEGG}},$$

where $s_{g,m,t}$ denotes the gene-level contribution for method $m$ under database $t$, and $G_{H}$ and $G_{K}$ denote the sets of genes retained for scoring under Hallmark and KEGG, respectively.

If a method’s cumulative database-specific score is negative after penalization, it is truncated to zero. The composite score shown in Figure 4d is finally defined as

$$\mathrm{TotalScore}_{m}=\mathrm{HallmarkScore}_{m}+\mathrm{KEGGScore}_{m}.$$

Accordingly, the Figure 4d scoring system is not based simply on the number of enriched pathways. Rather, it quantifies each method’s ability to maintain balanced activation–suppression behavior at the pathway level while explicitly accounting for non-computable outcomes. This design enables a comparable summary of performance across methods, databases, and datasets.

**Supplementary Note 2**

**Statistical clarification for Figure 4c**

For Figure 4c, we analyzed the percentage ratio, defined as the ratio of positive to negative core-enriched gene percentages across methods.
The analysis used 3 genes (CFD, ANKRD35, ALOXE3) × 2 pathway databases (Hallmark, KEGG) × 5 methods, and was performed separately for unique and notunique data. Therefore, each panel contained $n=30$ observations, with $n=6$ per method.

Within each panel, we first applied a Kruskal-Wallis test across the five methods, followed by pairwise Wilcoxon rank-sum tests with Bonferroni correction (p.adjust.method = "bonferroni").

The number of pairwise comparisons per panel was $\left( \frac{5}{2} \right)=10$.
