# Supplementary material for: Comparative evaluation of gene selection approaches in transcriptomics: bias correction and visualization with TransPro
Source: Gigascience. 2026 May 18;15:giag057. doi: 10.1093/gigascience/giag057 (PMC13215095; doi:10.1093/gigascience/giag057)
Supplement: giag057_GIGA-D-26-00052_original_submission [file giag057_giga-d-26-00052_original_submission.pdf]

GigaScience

Comparative Evaluation of Differential Gene Selection Methods in Transcriptomics: Bias Correction and Visualization with TransPro

--Manuscript Draft--

**Manuscript Number:**	GIGA-D-26-00052
**Full Title:**	Comparative Evaluation of Differential Gene Selection Methods in Transcriptomics: Bias Correction and Visualization with TransPro
**Article Type:**	Technical Note
**Funding Information:**	National Natural Science Foundation of China (62473212) — Professor Shengquan Chen<br>National Natural Science Foundation of China (62203236) — Professor Shengquan Chen<br>National Natural Science Foundation of China (32130014) — Prof Wenjun Bu

Abstract:

Background

Differential gene selection is fundamental to transcriptomics; however, mainstream methods typically fit each gene independently via marginal analyses. Although multifactor designs can be accommodated, gene-gene interactions are not explicitly modeled, rendering results susceptible to systematic bias driven by coexpression patterns. Additionally, heterogeneous tools, inconsistent metrics, and fragmented workflows compromise the reproducibility and interpretability of downstream enrichment and visualization analyses.

Findings

We developed TransPro, an open-source integrated framework comprising two complementary packages, TransProPy and TransProR, for systematic benchmarking, bias correction, and reproducible visualization in differential gene selection. TransProPy (Python) combines multivariate AUC-based complementarity quantification with ensemble learning for interaction-aware gene selection, whereas TransProR (R) provides standardized differential analysis, pathway enrichment, and seven visualization workflows (circular dendrograms, chord diagrams, spiral plots, and interaction networks). Cross-dataset generalizability was assessed using 12 independent datasets spanning multiple cancer types and normal tissues with broad heterogeneity in data origin, platform, batch structure, and sample size. Under stringent thresholds, positively and negatively correlated genes maintained a near-equal proportion at the gene level, and activated and suppressed pathway proportions exhibited good concordance with gene-level correlation patterns; critically, TransProPy produced meaningful enrichment results under conditions where conventional methods failed. Quantitative comparisons of core enriched gene proportions further supported these differences (Kruskal–Wallis and pairwise Wilcoxon tests, $p < 0.001$).

Conclusions

TransPro establishes a unified, reproducible framework that corrects method-specific biases while bridging computational discovery and biological interpretation. All code, workflows, documentation, and example data are openly accessible to support reproducibility and community reuse.

**Corresponding Author:**	Shengquan Chen<br>Nankai University School of Mathematical Science<br>Tianjin, Tianjin CHINA
**Corresponding Author Secondary Information:**	
**Corresponding Author's Institution:**	Nankai University School of Mathematical Science

Powered by Editorial Manager® and ProduXion Manager® from Aries Systems Corporation

Corresponding Author's Secondary Institution:	
First Author:	Dongyue Yu
First Author Secondary Information:	
Order of Authors:	Dongyue Yu
	Chen Li
	Shuo Yan
	Lujiale Guo
	Jingyu Liang
	Shengquan Chen
	Wenjun Bu
Order of Authors Secondary Information:	
Additional Information:	
**Question**	**Response**
Are you submitting this manuscript to a special series or article collection?	No
**Experimental design and statistics**<br><br>Full details of the experimental design and statistical methods used should be given in the Methods section, as detailed in our Minimum Standards Reporting Checklist. Information essential to interpreting the data presented should be made available in the figure legends.<br><br>Have you included all the information requested in your manuscript?	Yes
**Resources**<br><br>A description of all resources used, including antibodies, cell lines, animals and software tools, with enough information to allow them to be uniquely identified, should be included in the Methods section. Authors are strongly encouraged to cite Research Resource Identifiers (RRIDs) for antibodies, model organisms and tools, where possible.<br><br>Have you included the information requested as detailed in our Minimum	Yes

Powered by Editorial Manager® and ProduXion Manager® from Aries Systems Corporation

Standards Reporting Checklist?	
**Availability of data and materials**<br><br>All datasets and code on which the conclusions of the paper rely must be either included in your submission or deposited in publicly available repositories (where available and ethically appropriate), referencing such data using a unique identifier in the references and in the "Availability of Data and Materials" section of your manuscript.<br><br>Have you have met the above requirement as detailed in our Minimum Standards Reporting Checklist?	Yes
GigaScience has policies and guidelines in place for the use of generative AI-writing tools such as ChatGPT. If you have used such writing tools to assist with writing the manuscript this must be declared and cited in the text. Authors should not list AI-writing tools and other AI-assisted technologies as an author or co-author and should acknowledge that they are fully responsible for text generated or refined by AI-writing tools.<p><br><br>A summary of use (particularly in the introduction or among methods) needs to be included at the end of the paper, and the outputs should also be included as a supplementary file hosted in GigaDB or other open repositories. Please read our guidelines for more information. <p><br><br>By submitting to GigaScience, you are aware of the journal's AI-writing tools policy, and if you have declared use of such tools below, you have acknowledged this where appropriate in your manuscript and have made a summary of use and outputs available. <p><br><b>AI-assisted writing tools have been used in the preparation of this manuscript?	No

Manuscript
Click here to access/download;Manuscript;manuscript.docx

Comparative Evaluation of Differential Gene Selection Methods in Transcriptomics: Bias Correction and Visualization with TransPro

Dongyue Yu[1], Chen Li[2], Shuo Yan[3], Lujiale Guo[4], Jingyu Liang[1], Shengquan Chen[5,*] and Wenjun Bu[1,*]

[1]Institute of Entomology, College of Life Sciences, Nankai University, Tianjin 300071, China

[2] Tianjin Medical University Cancer Institute and Hospital, Tianjin 300071, China

[3]AI Thrust, The Hong Kong University of Science and Technology (Guangzhou), Guangzhou 510000, China

[4] Zhongshan Hospital of Fudan University, Shanghai 200032, China

[5]School of Mathematical Sciences and LPMC, Nankai University, Tianjin 300071, China

*Corresponding authors. Shengquan Chen, School of Mathematical Sciences and LPMC, Nankai University, Tianjin 300071, China. Email: chenshengquan@nankai.edu.cn; Wenjun Bu, Institute of Entomology, College of Life Sciences, Nankai University, Tianjin 300071, China. Email: wenjunbu@nankai.edu.cn

Abstract

Background: Differential gene selection is fundamental to transcriptomics; however, mainstream methods typically fit each gene independently via marginal analyses. Although multifactor designs can be accommodated, gene-gene interactions are not explicitly modeled, rendering results susceptible to systematic bias driven by coexpression patterns. Additionally,

heterogeneous tools, inconsistent metrics, and fragmented workflows compromise the reproducibility and interpretability of downstream enrichment and visualization analyses.

Findings: We developed TransPro, an open-source integrated framework comprising two complementary packages, TransProPy and TransProR, for systematic benchmarking, bias correction, and reproducible visualization in differential gene selection. TransProPy (Python) combines multivariate AUC-based complementarity quantification with ensemble learning for interaction-aware gene selection, whereas TransProR (R) provides standardized differential analysis, pathway enrichment, and seven visualization workflows (circular dendrograms, chord diagrams, spiral plots, and interaction networks). Cross-dataset generalizability was assessed using 12 independent datasets spanning multiple cancer types and normal tissues with broad heterogeneity in data origin, platform, batch structure, and sample size. Under stringent thresholds, positively and negatively correlated genes maintained a near-equal proportion at the gene level, and activated and suppressed pathway proportions exhibited good concordance with gene-level correlation patterns; critically, TransProPy produced meaningful enrichment results under conditions where conventional methods failed. Quantitative comparisons of core enriched gene proportions further supported these differences (Kruskal–Wallis and pairwise Wilcoxon tests, $p < 0.001$).

Conclusions: TransPro establishes a unified, reproducible framework that corrects method-specific biases while bridging computational discovery and biological interpretation. All code, workflows, documentation, and example data are openly accessible to support reproducibility and community reuse.

Graphical Abstract

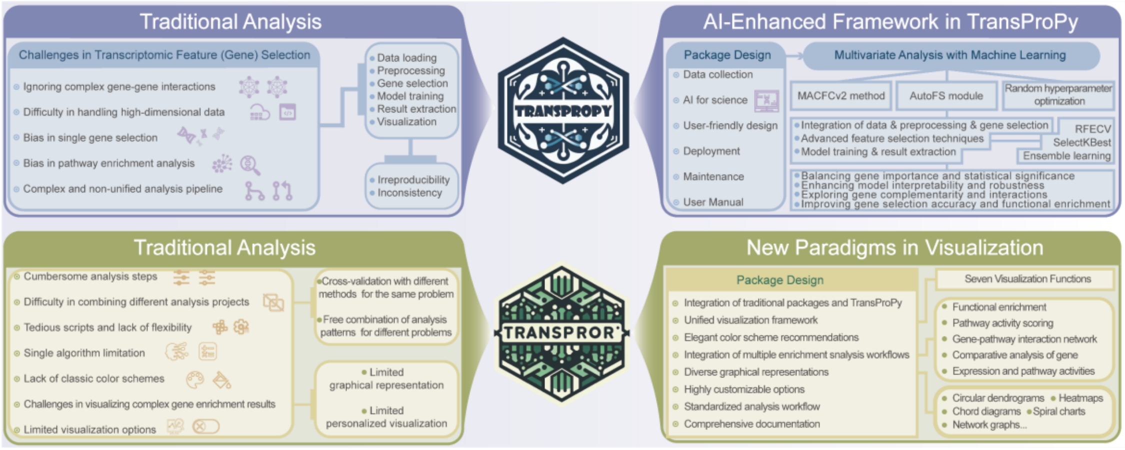

Keywords Differential gene selection · Transcriptomics · Correlation bias · Multivariate feature selection · Standardized workflow · Multidimensional visualization · Gene set enrichment analysis

1 Introduction

The identification of functional genes associated with specific biological processes or diseases is a cornerstone of modern genomic research, providing the foundation for understanding complex biological mechanisms and developing targeted therapies. Feature selection plays a particularly crucial role in this process, referring to the extraction of the most informative genes (features) from large-scale, high-dimensional biological datasets, such as bulk RNA-Seq [1], single-cell RNA-Seq [2-4], and microarray data [5]. These features typically represent gene expression levels and are selected based on their relevance and statistical significance in distinguishing between different phenotypes, such as healthy and diseased states. Traditionally, feature selection methods have focused on identifying genes that are independently associated with particular phenotypes. While this approach can be effective in certain contexts, it often falls short when it comes to capturing the intricate interactions among genes that contribute to the full expression of biological traits. Genes do not operate in isolation; they interact within complex networks, where the collective effect of multiple genes provides deeper insights into biological processes than any single gene alone.

In practical transcriptomic workflows, differential expression (DE) analysis is also frequently used as an initial screening step for feature selection. However, DE methods are primarily designed to answer “which genes change significantly on their own,” whereas phenotype discrimination often depends on multigene combinations whose joint effect may be weak or invisible in marginal, gene-by-gene tests. Therefore, while DE-based rankings remain indispensable baselines, advancing transcriptomic feature selection requires methods that explicitly consider multivariate complementarity and interaction effects.

In the field of gene selection and differential expression analysis, DESeq2 [6], edgeR [7], limma [8, 9], and the Wilcoxon rank-sum test (WRST) [10] are widely used classical methods. These approaches are based on statistical models that assess changes in the expression levels of individual genes. Although these methods are effective in standard differential expression settings, they exhibit limitations when applied to complex biological systems and high-dimensional datasets. DESeq2 and edgeR are built on the negative binomial distribution, making them especially suitable for modeling count-based RNA-Seq data. By incorporating overdispersion parameters, they account for biological and technical variability, enhancing model flexibility. However, in cases where data noise is high or sample distributions deviate from the negative binomial assumption, these methods may not fully capture the data's complexity. Although DESeq2 incorporates Cook's distance–based outlier detection, extreme distributional violations can still compromise model fitting. Additionally, they tend to prioritize highly expressed genes, while lowly expressed or rare genes often remain undetected, contributing to an increased false negative rate. By comparison, limma, which is based on a linear model, is adept at handling various types of high-throughput data such as RNA-Seq and

microarrays. Although it excels in estimating gene expression changes, the model's simplifications can introduce bias when dealing with complex gene interactions, as each gene is tested marginally without explicit modeling of inter-gene dependencies. The Wilcoxon rank-sum test (WRST), a non-parametric method, is appropriate for data that do not meet the normality assumption, but as a univariate test it cannot capture synergistic effects between genes, and its discovery rate is further reduced by large-scale multiple-testing correction.

To address these limitations, advanced methods have made important progress along different directions. The MACFC algorithm [11] introduced the multi-variable Area Under the Curve (mvAUC) metric to identify complementary gene features, demonstrating superior performance over methods including FAST [12], ARCO [13], AVC [14], ReliefF [15], mRMR [16], MRI [17], and PAM [18] in benchmark evaluations across UCI, TCGA [19], and Microarray platforms, revealing its capacity to capture synergistic effects among features. In parallel, Automated Machine Learning (AutoML) frameworks have substantially improved analytical efficiency through end-to-end model selection and hyperparameter optimization. Building on this foundation, AutoGluon-Tabular [20] advanced further by employing layered ensemble strategies that combine boosted trees with neural networks, achieving superior performance through multi-layer stacking with skip connections and repeated k-fold bagging, surpassing platforms such as Auto-WEKA [21, 22], auto-sklearn [23], TPOT [24], H2O AutoML, and GCP-Tables in both speed and accuracy. Yet transcriptomics research faces systemic challenges rather than isolated technical bottlenecks. Existing tools typically excel in specific aspects—feature selection, model construction, or result presentation—but struggle to simultaneously address balanced coverage of diverse correlation patterns in gene selection, comprehensiveness and impartiality in pathway

enrichment analysis, accurate localization of key regulatory genes within complex networks, and integrated presentation of cross-method analytical results with multi-layered biological information. What transcriptomics research demands is a comprehensive solution that integrates the strengths of complementarity analysis and automated modeling, incorporates deep customization for domain-specific characteristics, and bridges the entire workflow from feature selection through multi-dimensional visualization.

Building on these insights, the TransPro project has been developed to extend these advanced methodologies into the realm of transcriptomics data analysis. While not an AutoML system, TransProPy—the Python-based toolkit within the TransPro project—adopts a similar philosophy by integrating advanced machine learning techniques and multivariate interaction analyses. TransProPy is specifically designed to evaluate the complementarity and interactions among gene features, thereby addressing the limitations of traditional univariate approaches. This focus on complex gene interactions within transcriptomics data ensures that TransProPy meets the specific demands of transcriptomics analysis. Further advancing these capabilities, TransProR—the R-based counterpart—offers a well-integrated suite of visualization tools that seamlessly combine custom workflows and scripts with established R packages. This framework is designed to accommodate diverse data types and scalable analytical requirements, effectively addressing the intrinsic limitations of traditional approaches while enabling researchers to intuitively explore and reconstruct the intricate relationships within gene enrichment data. By combining TransProPy's robust analytical power with TransProR's advanced visualization capabilities, TransPro establishes a synergistic and nuanced approach to transcriptomic data analysis. This toolset bridges the gap between machine learning and genomics, promising to play a pivotal role

in the future of biological and medical research while offering profound insights into gene function and its impact on health and disease (Figure 1).

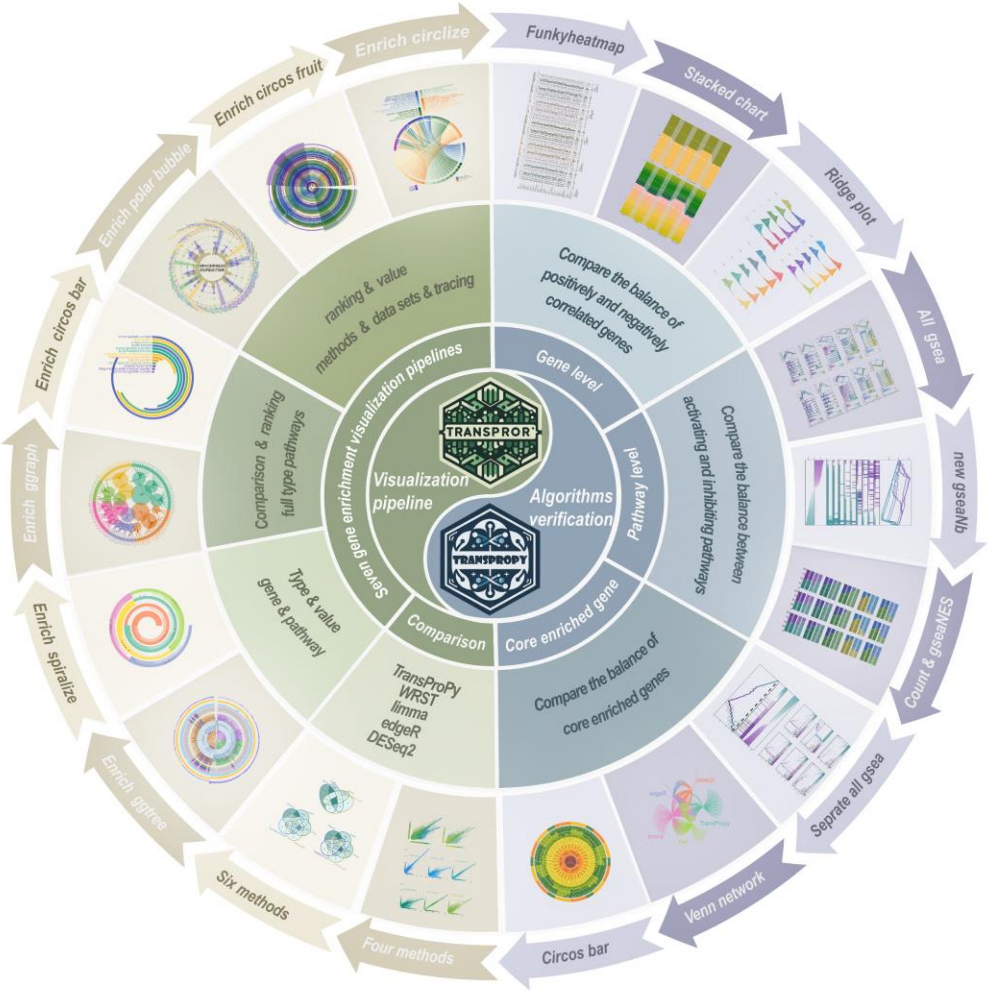

Figure 1. Schematic layout of the TransPro project. The circular diagram illustrates the complete workflow of the TransProR and TransProPy pipelines in gene enrichment and pathway analysis. The central portion of the diagram represents the two core modules, each designed as a systematic analysis pipeline, enabling multilevel visualization and robust algorithm validation. The middle ring divides the workflow into three primary domains: the gene-level, which focuses on visualization and ranking methods for tracking dataset performance; the core-enriched gene level, which compares how different methods handle the balance of core-enriched genes; and the pathway level, which highlights the comparison of activation and inhibition pathway balance, showcasing key pathway analysis and method validation. The outer ring details the algorithm validation section, which compares multiple methods, including TransProPy, DESeq2, edgeR, limma, and the Wilcoxon Rank-Sum Test,

evaluating their performance across different levels. Another section is dedicated to comprehensive gene and pathway visualization and comparison, utilizing seven visualization pipelines: Enrich ggtree, Enrich spiralize, Enrich ggraph, Enrich circos bar, Enrich polar bubble, Enrich circos fruit, and Enrich circlize.

2 Findings

2.1 TransProPy achieves balanced positive-negative distribution in gene correlation analysis

All four classical methods consistently exhibited an elevated proportion of positively correlated genes in gene correlation analysis, revealing a pronounced imbalance in correlation distribution (Figures 2a, 5g). In contrast, TransProPy yielded the most balanced proportion between positively and negatively correlated genes, highlighting its superior capacity for mitigating correlation bias (Figures 2a, 5g). To further validate this advantage under extreme conditions, we selected the top three genes exhibiting the largest inter-method discrepancies—CFD, ANKRD35, and ALOXE3—for in-depth analysis, among which CFD showed the greatest disparity in the number of negatively correlated genes compared to other methods, with the largest margin relative to the second-ranked method (Figure 2b, 2c). Correlation distribution analysis of these three genes revealed that all five methods displayed bimodal characteristics, with weakly correlated genes being sparse while strongly positive and negative correlations increased sharply; however, conventional methods demonstrated a markedly higher positive correlation peak relative to the negative correlation peak, whereas TransProPy exhibited a correlation distribution symmetrically centered around zero, with the ratio of the absolute positive-correlation median to the absolute negative-correlation median closest to 1 (Figure 2d; Supplementary Figures 1-3). Even under these maximum contrast intensities, TransProPy maintained a balanced positive-

negative distribution while identifying strongly correlated genes, further substantiating its practical applicability.

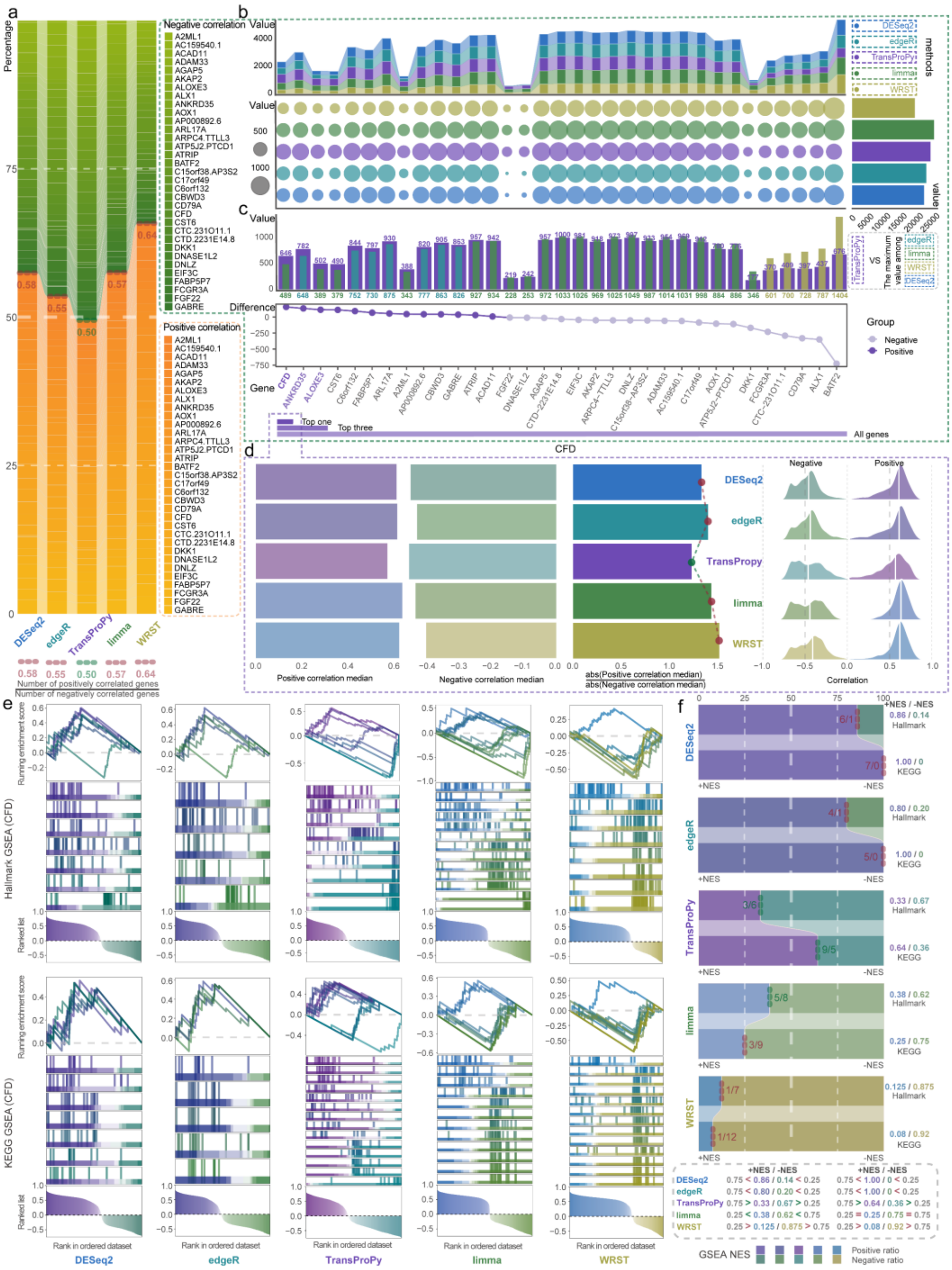

Figure 2. Comprehensive visualization of gene correlation trends and pathway enrichment across multiple gene selection methods. a Stacked bar plot illustrating the overall distribution of selected genes from 33 candidates across the five methods: DESeq2, edgeR, limma, WRST, and TransProPy. Each bar represents the total number of genes identified, with distinct color sections indicating the proportion of positively and negatively

correlated genes. **b** The stacked area chart (top) and bubble chart (bottom) display the number of genes strongly correlated (using negatively correlated genes as an example) for each method (DESeq2, edgeR, limma, WRST, and TransProPy) across 33 genes. The area chart visualizes the quantity of correlated genes for each gene by method, assessing whether there is a consistent trend in gene selection quantity across all methods. The bubble chart emphasizes the relative values of gene quantities, with larger bubbles indicating methods that have selected more genes. The bar chart on the right summarizes the total number of genes selected by each method. **c** For negatively correlated genes, the bar chart (top) compares the number of negatively correlated genes in the TransProPy method with the maximum negatively correlated gene counts from the other four methods: edgeR, limma, WRST, and DESeq2, with the differences sorted in descending order (bottom). **d** The ridge density plot illustrates the correlation distribution of the CFD gene across different methods, with the white semi-transparent lines indicating the median position (right). The bar chart shows the magnitude of the median for positive and negative correlations and their ratio (left). **e** Gene Set Enrichment Analysis (GSEA) is performed on the CFD gene using hallmark and KEGG gene sets, with visualizations that delineate the activation and suppression of pathways. **f** Comparison of the ratio of activated and suppressed pathways across different methods, highlighting the balanced feature selection achieved by TransProPy at the gene pathway level. The +/- NES signifies if the normalized enrichment score is positive (indicated by +NES) or negative (indicated by -NES).

2.2 TransProPy demonstrates unbiased bidirectional detection across gene and pathway levels

In DESeq2 and edgeR, although the overall proportion of positively and negatively correlated genes approached 0.5, the proportion of positively correlated genes significantly exceeded that of negatively correlated genes when $|\rho| > 0.5$, indicating that selection bias intensified with increasing correlation strength (Figure 3a). This trend became more pronounced in GSEA, where the number of activated pathways far exceeded suppressed pathways, and in some cases, no suppressed pathways were detected at all—a pattern sharply contrasting with the proportional trends observed at the gene correlation level. Conversely, although in limma and WRST the proportion of positively correlated genes consistently exceeded that of negatively correlated genes, with the selection bias further amplified when $|\rho| > 0.5$ (Figure 3a), the GSEA trend was reversed: activated pathways were fewer than suppressed pathways, and WRST exhibited a

marked reduction in activated pathways, clearly contradicting the gene-level trends. Further analysis revealed that many suppressed pathways enriched by limma and WRST were nearly identical in gene ranking and composition, suggesting these pathways were synonymous or highly similar (i.e., different designations or sub-pathways of the same pathway category) rather than entirely independent pathways (Figure 2e; Supplementary Figures 4-5). Therefore, the apparent advantage of limma and WRST in enriching more suppressed pathways (Figure 2f) was of limited significance, as excessive redundancy increased noise in the results. In contrast, TransProPy exhibited the most balanced performance among the five methods: even for genes with $|\rho| > 0.5$, the proportion of positively and negatively correlated genes remained close to 0.5 with minimal fluctuation, and the ratio of activated to suppressed pathways showed the strongest consistency with gene correlation proportions, both approaching 0.5 (Figures 2e, 2f, 3a). These findings demonstrate that TransProPy effectively balances feature identification at both gene and pathway levels—encompassing positive and negative gene correlations as well as pathway activation and suppression—thereby preventing result bias arising from data imbalance or algorithmic assumptions while comprehensively accounting for gene behavior and their interactions.

2.3 TransProPy consistently enriches pathways across all test conditions where other methods fail

Comprehensive GSEA was performed on all 33 genes, with each gene analyzed 20 times per method to eliminate random effects. Results revealed that in both KEGG and Hallmark enrichment, the proportions of DESeq2 and edgeR were diametrically opposite to those of limma

and WRST, whereas TransProPy exhibited the most balanced performance, occupying an intermediate position (Figure 3b). In KEGG enrichment, the other four methods failed to enrich pathways in certain cases—with DESeq2 being the most severely affected—while TransProPy was the only method that successfully enriched pathways under all conditions (Figure 3b, Supplementary Figures 6–10). The ability of TransProPy to achieve balance in both gene correlation and pathway enrichment analyses highlights its comprehensive and precise methodological advantages, effectively avoiding the bias and redundancy present in other methods, and consolidating its value in revealing the deeper complexities of gene interactions.

2.4 TransProPy identifies both shared core genes and unique signatures to enhance enrichment analysis

Detailed examination of gene rankings and enrichment scores within each individual pathway confirmed that no aberrant genes exerted undue influence on pathway-level results. Venn network visualization of core enriched genes across all significant pathways revealed that, despite algorithmic differences, the five methods collectively identified a set of the most central enriched genes. Furthermore, TransProPy, limma, and WRST each possessed unique signature genes that effectively supplemented the analytical results, whereas DESeq2 and edgeR contributed relatively few or no unique genes (Figure 3c). The lack of additional signature genes to balance the results may be an important reason for the pathway enrichment bias observed in previous analyses—where pathways were predominantly activated with few or no suppressed pathways (Figure 3a). Therefore, compared with other algorithms, TransProPy not only excels in accurately identifying core enriched genes but also contributes additional signature genes that

effectively address the limitations of other methods. Its balance and comprehensiveness establish it as a critical tool for capturing the complexity of biological regulatory mechanisms, significantly enhancing the robustness and precision of enrichment analysis.

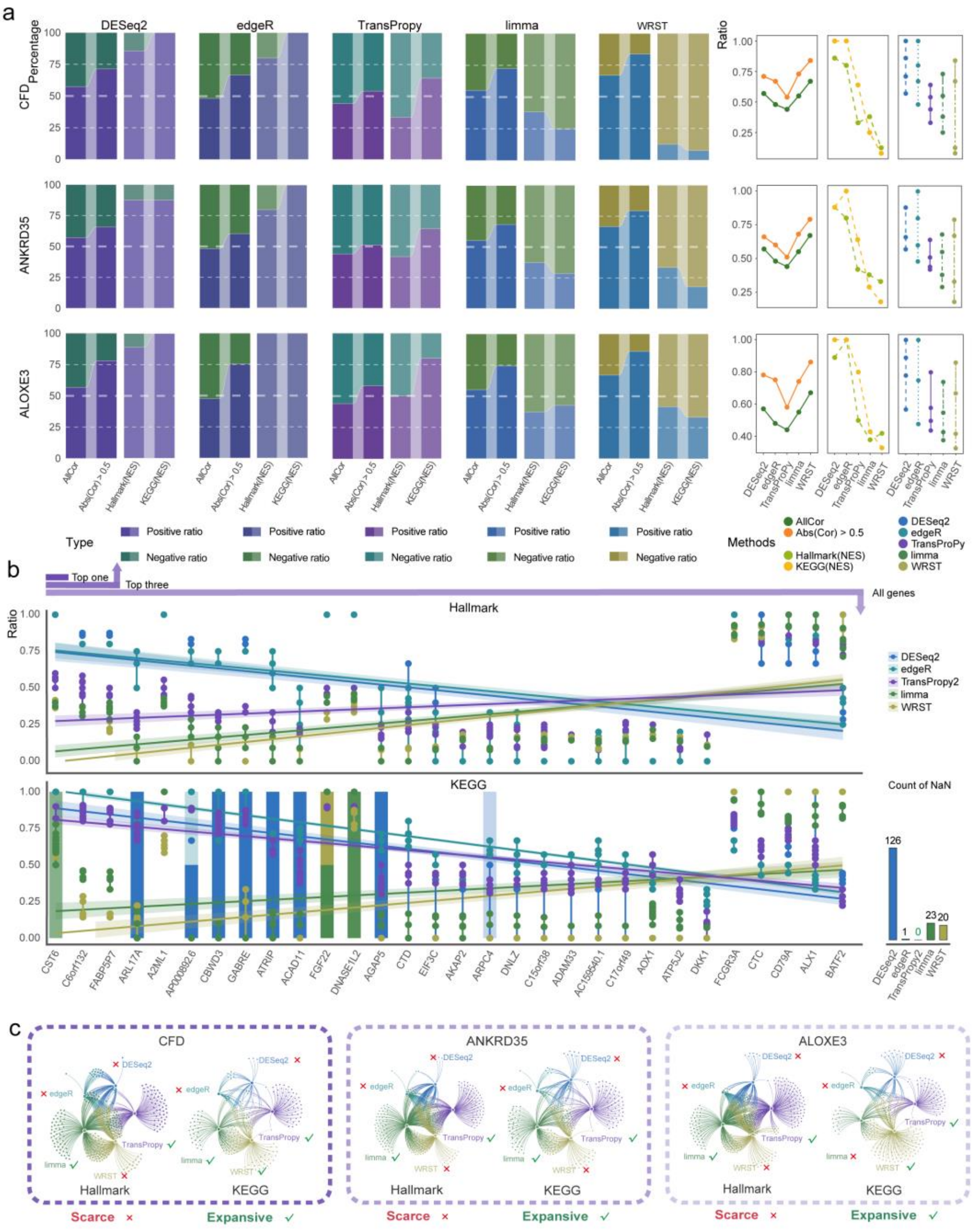

Figure 3. Comprehensive Analysis of Pathway Enrichment, Pathway Type Distribution, and Core Enriched Gene Analysis Across Different Methods. a The stacked bar graphs display the top three genes from Figure 2c, categorized by pathway type (Hallmark and KEGG), illustrating the variations in pathway activation and suppression across different methods

(DESeq2, edgeR, TransProPy, limma, WRST). Adjacent line charts highlight the dynamic changes in the ratios of positively and negatively correlated genes or pathways, emphasizing how these ratios evolve with increasing gene correlation strength and varying pathway types. **b** This integrated visualization combines scatter and line plot elements to depict the enrichment analysis of 33 genes across KEGG and Hallmark pathways, iterated 20 times for each method. It captures the ratio of activated to suppressed pathways, illustrating the impact of each method's selection criteria on the observed outcomes. **c** Network diagrams demonstrate the connectivity and overlap of core enriched genes across methods within specific pathways (*CFD, ANKRD35, ALOXE3*), distinguishing unique and overlapping genes in Hallmark and KEGG pathways.

2.5 Quantification of core enriched genes reveals amplification effects and TransProPy's balanced detection

Core enriched genes in activated and suppressed pathways were quantified using both deduplicated and non-deduplicated versions. Results showed that the imbalanced ratio of negatively to positively regulated genes in the deduplicated group (30 groups) was further amplified in the non-deduplicated group (30 groups), attributable to "super core enriched genes"—genes that recurrently appear across multiple pathways—exerting regulatory roles across a broader range of pathways, thereby appearing repeatedly in the statistics of core enriched genes across all pathways (Figure 4a). If the deduplicated group consisted entirely of ordinary core genes or super core genes with identical repetition rates, the gene proportions would remain unchanged in the non-deduplicated group.

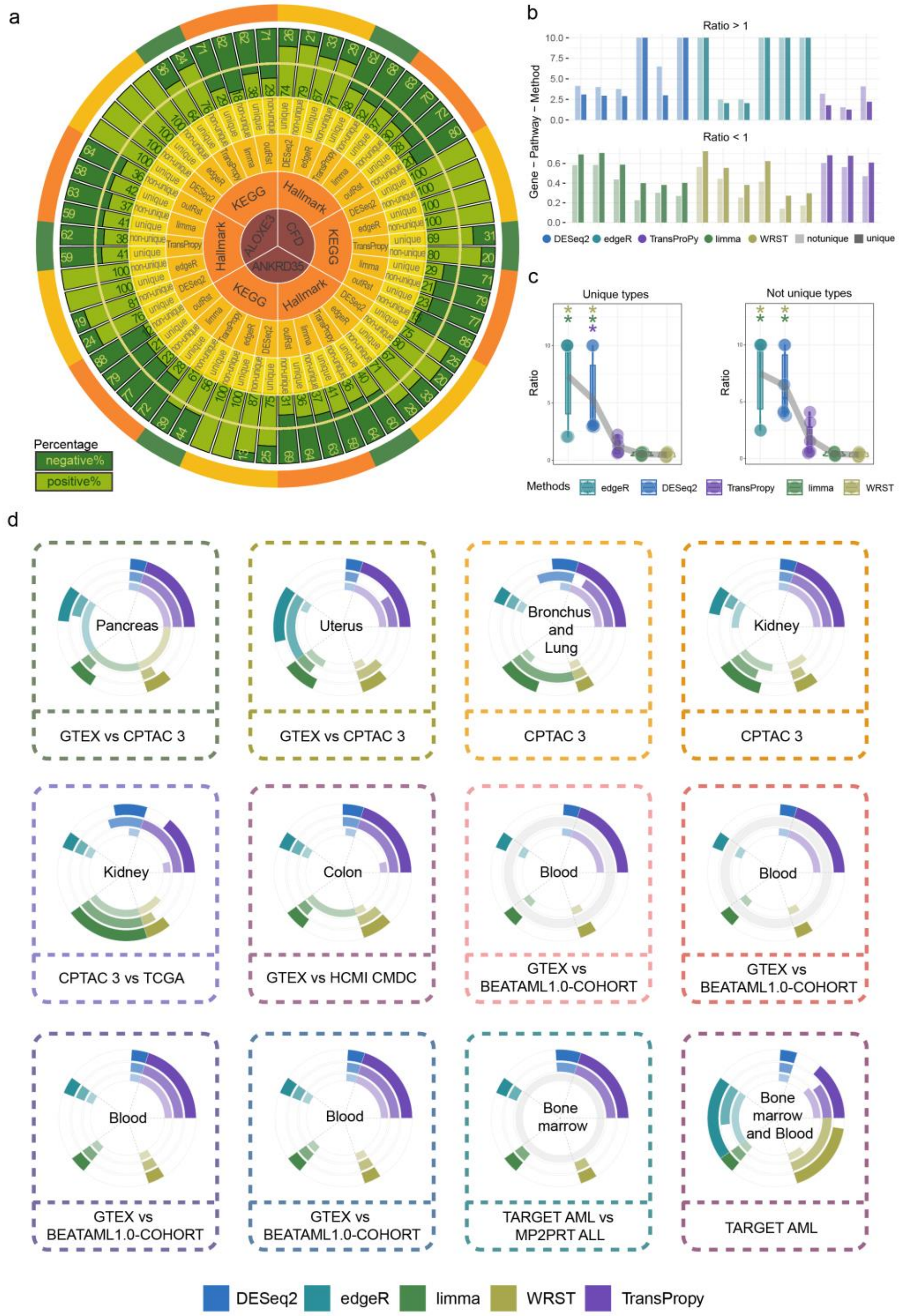

Figure 4. Comparative assessment of gene selection methods in pathway enrichment balance and cross-tissue performance. a Each segment of the circos plot depicts the distribution of core enriched genes by pathway type, comparing deduplicated and non-deduplicated groups. This visualization highlights super core enriched genes appearing in multiple pathways across various genomes and methods. **b** Histograms show the distribution of pathway enrichment ratios (positive/negative) for each method, classified by cases where the

ratio is greater than or less than 1. **c** Scatter plots comparing the proportions of unique versus non-unique core enriched genes (Kruskal–Wallis test, $p < 0.001$ for both panels; pairwise comparisons by Wilcoxon test), revealing each method's gene selection strategy and its influence on the balance between pathway activation and suppression. **d** Performance evaluation of gene selection methods across tissues and pathway databases. The panel comprises 12 independent circular plots, each representing a specific tissue-database combination. Each plot contains three concentric rings: inner ring (Hallmarks) showing method scores on Hallmark gene sets; middle ring (KEGG) displaying scores on KEGG pathway sets; outer ring (Total Score) presenting combined scores across both databases. Segments within each ring represent individual analytical methods (blue: DESeq2; light blue: edgeR; green: limma; light green: WRST; purple: TransProPy), with arc lengths proportional to scores and progressively expanding outward to emphasize total performance. Rings with zero scores are rendered in light gray.

However, if the number or repetition rate of super core genes in one category (activated or suppressed) of pathways exceeded that of the other category, the gene proportions would further increase in the non-deduplicated group. An ideal algorithm should identify more super core genes or those with higher repetition rates, indicating greater importance. This effect was evident across all five algorithms. Notably, TransProPy was the only method in which both ratios >1 and <1 were observed, each accounting for half of the cases, in contrast to the exclusively >1 ratios in DESeq2 and edgeR and the exclusively <1 ratios in limma and WRST (Figure 4b). In both deduplicated and non-deduplicated groups, DESeq2 and edgeR showed proportions of positively correlated core enriched genes exceeding negatively correlated ones (with some ratios approaching 1), indicating potential bias; limma and WRST showed positively correlated proportions lower than negatively correlated ones (with some positive-to-negative ratios approaching 0); TransProPy's positive-to-negative ratios fell between those of DESeq2 and edgeR and limma and WRST, demonstrating a smooth transitional trend and yielding the most balanced results (Figures 4a, 4c; Kruskal–Wallis $p < 0.001$, Wilcoxon pairwise comparisons).

2.6 Cross-tissue and cross-database validation demonstrates the universal superiority of

TransProPy

To validate the generalizability of our conclusions across broader and more complex biological contexts, we performed comprehensive analyses on multiple datasets from different sources, batches, sample sizes, and biological backgrounds, encompassing pancreatic cancer, uterine cancer, lung cancer, kidney cancer, colon cancer, hematological malignancies, and their corresponding normal tissues. Data were obtained from authoritative projects such as GTEx, CPTAC, TCGA, and TARGET, among others, ensuring the comprehensiveness and generalizability of the evaluation. The circular plots in Figure 4d comprehensively illustrate the performance of the five methods (DESeq2, edgeR, limma, WRST, and TransProPy) across 12 different datasets and tissue types. Each plot adopts a hierarchical ring structure, with concentric rings representing scores for Hallmark, KEGG, and the combined total score from the innermost to the outermost ring, respectively. Arc length is proportional to the score, colors represent different methods, and transparency increases from inner to outer rings to emphasize total score performance; rings with all-zero values are displayed as complete light gray circles. The results demonstrated that in the vast majority of analytical scenarios, the purple segments representing TransProPy occupied the largest arc length in the outer ring (total score), indicating that the overall performance of TransProPy surpassed that of the other four conventional methods across various biological contexts. This extensive validation not only confirmed the superiority of TransProPy on specific datasets but, more importantly, established its reliability as a general-purpose tool across different biological contexts, providing a solid evidence base for its broad application in complex biomedical research.

3 Methods

3.1 The MACFC algorithm for feature selection based on mvAUC

TransProPy adopts an mvAUC-based MACFC framework for gene feature selection [11]. Its core principle is global complementarity quantification with redundancy suppression: mvAUC measures the net gain in classification performance when a feature is considered jointly with others, thereby prioritizing synergistic feature combinations rather than relying solely on single-gene effects. To accommodate different levels of gene–trait association, two complementary implementations are provided: MACFCmain, for weakly associated data, emphasizes broad capture of trait-relevant features; MACFCv2, for strongly associated data, first applies an AUC threshold to preselect high-performing candidates and then performs combinatorial optimization to enhance interpretability and robustness while reducing redundancy without compromising performance. Using AUC and mvAUC as unified evaluation criteria, the method iteratively constructs optimal feature subsets and outputs ordered features with corresponding weights for downstream modeling and visualization.

3.2 Feature selection strategy based on ensemble models and optimization search

We propose AutoFS, an ensemble- and optimization-based feature selection strategy that builds an end-to-end pipeline unifying data loading and preprocessing, feature selection, model training, and result export. In the selection stage, a FeatureUnion combines recursive feature elimination with cross-validation (RFECV) and SelectKBest: RFECV uses an ensemble estimator of SVM, decision tree, and gradient boosting to iteratively remove redundancy based on cross-validation until the optimal subset is obtained, while SelectKBest complements this

with univariate statistical significance to balance model- and statistic-driven signals. RandomizedSearchCV then performs randomized hyperparameter search over a predefined space, and a stacking ensemble (StackingClassifier) fuses predictions from SVM, decision tree, and gradient boosting at the first level, with logistic regression serving as the meta-learner for second-level integration. AutoFS outputs a ranked core feature subset with importances and weights and provides standardized results ready for downstream modeling and visualization, ensuring robustness, interpretability, and generalization across varying data scales and distributions.

3.3 Data acquisition and gene set definition

To systematically evaluate the bias of traditional feature selection tools and their impact on downstream analyses, this study utilized TCGA-SKCM [19] (tumor n=470) and GTEx [25] skin tissue (normal n=811) data as examples. Four classical differential analysis methods—DESeq2 [6], edgeR [7], limma [8, 9], and WRST [10]—were evaluated based on pairwise correlations of logFC values (Figure 5a) and consistency of overall expression trends among differentially expressed genes (Figure 5b), and compared with TransProPy, which integrates AutoFS and MACFCv2. Through the intersection of all six methods, 33 commonly selected genes were identified as the target gene set (Figure 5c–5f), establishing a unified evaluation framework (Figure 5e).

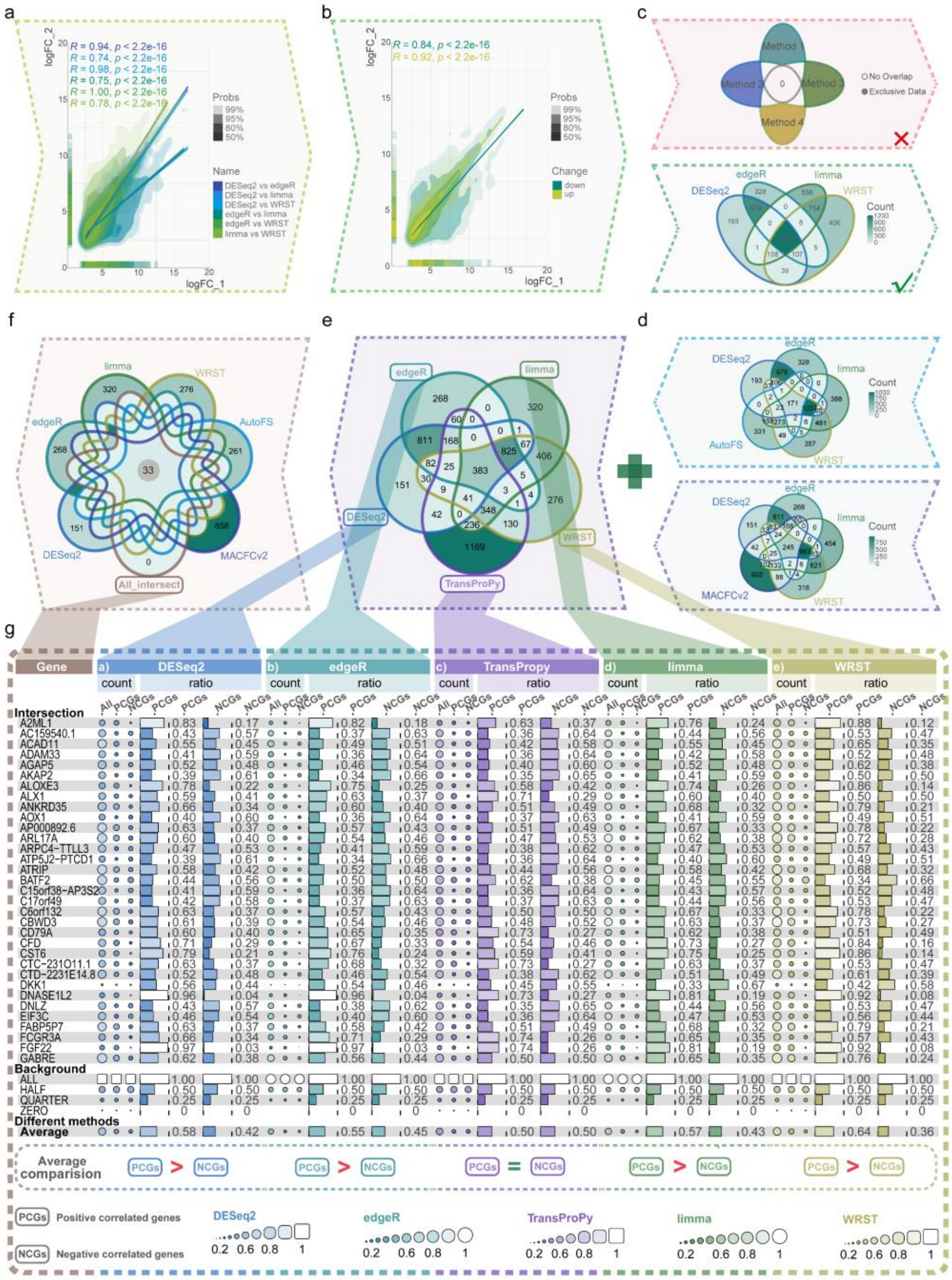

Figure 5. Comparative evaluation of five gene selection methods. **a** Density plot showing pairwise comparisons of log fold-change (logFC) values among the four methods (DESeq2, edgeR, limma, and WRST). The color gradient represents the density of overlapping values, with darker regions corresponding to higher density. **b** Density plot comparing logFC values, grouping DESeq2, edgeR, limma, and WRST into two categories based on upregulated and downregulated genes. The color gradient represents the density of overlapping values, with darker areas indicating higher gene density in both the upregulated (yellow-green) and downregulated (green) categories. **c** Venn diagrams showing two cases: one where no overlap

occurs between methods, and another where a substantial overlap exists between DESeq2, edgeR, limma, and WRST in identifying differentially expressed genes (DEGs). **d** Venn diagram showing the overlap between DESeq2, edgeR, limma, WRST, and the two TransProPy methods (AutoFS and MACFCv2), highlighting both the shared and unique aspects of gene selection. **e** Venn diagram showing the intersections between DESeq2, edgeR, limma, WRST, and the TransProPy method. The diagram highlights the shared DEGs across these five approaches, emphasizing both the commonalities in gene detection and the unique and overlapping genes among the methods. **f** Venn diagram highlighting the 33 genes consistently selected by all six methods. These genes form a core set of target genes for further feature correlation analysis. **g** Heatmap showing the response of each method to feature correlation. For each gene, the numbers of positively correlated genes (PCGs) and negatively correlated genes (NCGs) were calculated using Spearman's correlation coefficient ($|\rho| > 0.5$).

3.4 Assessment of gene correlation balance and validation under extreme conditions

For each of the 33 target genes, Spearman correlation coefficients were calculated against all other selected genes, with genes classified as positively correlated ($\rho > 0.5$) or negatively correlated ($\rho < -0.5$). The "correlation balance index" was defined as the average proportion of positively and negatively correlated genes relative to the total strongly correlated genes ($|\rho| > 0.5$) across all 33 targets. Distribution symmetry was evaluated by the ratio of median absolute values between positive and negative correlations, while selection scale consistency across the five methods was assessed using the number of negatively (or positively) correlated genes, including quantity, stacking trends, and overall magnitude. To rigorously test robustness, we selected the top three genes exhibiting the largest inter-method discrepancies for in-depth analysis; if TransProPy maintains both overall distribution consistency and its balanced correlation profile under these extreme conditions, it would provide strong evidence for superior generalizability and practical applicability.

3.5 Directional consistency validation and redundancy control in pathway enrichment

analysis

Gene Set Enrichment Analysis (GSEA) [26, 27] was performed using KEGG [28-31] and Hallmark [32, 33] gene sets to examine whether the ratio of activated to suppressed pathways at the pathway level maintains directional consistency with the ratio of positively to negatively correlated genes at the gene level. For each target gene under each method, the analysis was repeated 20 times to eliminate stochastic effects, and the proportions of positively and negatively enriched pathways were quantified. Pathways with highly similar gene rankings and overlapping gene compositions (including synonymous, homologous, and sub-pathways) were identified, and both deduplicated and non-deduplicated versions of core enriched gene statistics were constructed to quantify the impact of pathway redundancy on the robustness of conclusions.

3.6 Quantification of leading-edge genes, identification of super core genes, and intersection-union analysis

Three genes exhibiting the most pronounced correlation differences—CFD, ANKRD35, and ALOXE3—were selected for further analysis. For each gene, the number of leading-edge genes was quantified across both activated and suppressed pathways, with comparisons made between deduplicated and non-deduplicated results. This approach enabled the identification of "super core enriched genes" that recurrently appeared across multiple pathways, along with their amplifying effects on the observed imbalance. Overall comparisons were conducted using the Kruskal–Wallis test ($p < 0.001$), followed by pairwise Wilcoxon tests. Additionally, a Venn network was constructed to visualize the intersection and union of leading-edge genes identified by each method across all significantly enriched pathways, thereby elucidating the sources and

contributions of shared core genes versus method-specific complementary findings.

3.7 Cross-dataset generalization evaluation and statistical methods

To validate cross-dataset generalizability, we constructed 12 independent tissue–database combinations spanning diverse cancer types, platforms, and batch sources from multiple public repositories (e.g., GTEx, CPTAC, TCGA, and TARGET). Method scores were computed separately for Hallmark and KEGG gene sets and subsequently aggregated into a composite total score. Results were visualized using three-layer concentric rings (inner ring: Hallmark; middle ring: KEGG; outer ring: total score), with arc length proportional to the score; zero-score rings were displayed in light gray. The same statistical framework was applied to ratio-based metrics (activation/suppression and positive/negative correlation ratios). For each target gene, 20 iterations per method were performed to assess robustness.

3.8 R package architecture and visualization workflow

TransProR is developed upon R's core data science and statistical ecosystem, providing a highly modular and extensible analytical framework that consolidates complex multi-step workflows into intuitive, unified function calls. The architecture comprises four principal components: (i) a data preprocessing module that automates raw data retrieval, gene identifier conversion, expression matrix normalization, sample classification, and batch-effect correction for reliable multi-source integration; (ii) a differential expression module offering a unified interface that adaptively selects optimal preprocessing and analytical strategies based on input data characteristics; (iii) a network and pathway analysis module enabling streamlined enrichment analyses through seamless integration with established annotation tools; and (iv) a visualization

and utility module supporting adaptive graphical customization, legend rendering, and publication-ready figure generation. The modular design permits both independent and combined use of functional components, with extensive parameter configurations enabling end-to-end customization from data processing to final visual output.

4 Discussion

TransPro establishes an integrated analytical ecosystem for transcriptomic research through two complementary packages: TransProPy for robust feature selection and TransProR for comprehensive visualization. Classical differential expression methods—DESeq2, edgeR, limma, and WRST—are prone to directional selection bias during gene screening, skewing the balance between positively and negatively correlated genes; this imbalance can propagate downstream along the analytical pipeline, manifesting at the pathway level as systematic distortion in the proportions of activated and suppressed pathways driven by method-specific bias. By integrating mvAUC-based complementarity quantification with ensemble-optimized feature selection, TransProPy maintains near-equal proportions of positively and negatively correlated genes even under stringent correlation thresholds ($|\rho| > 0.5$), and the pathway-level activation–suppression enrichment profile exhibits good concordance with the gene-level correlation distribution; notably, under certain test conditions where other methods failed to yield valid pathway enrichment results, TransProPy consistently completed the analysis. Furthermore, TransProPy identifies both shared core enriched genes across methods and method-specific signature genes that effectively supplement the analytical results; quantification of super core enriched genes further reveals that TransProPy alone

exhibits bidirectional amplification effects (ratios both >1 and <1), whereas conventional methods tend toward unidirectional bias. Cross-tissue and cross-database validation across 12 independent datasets from GTEx, CPTAC, TCGA, TARGET, and other repositories—encompassing multiple cancer types and normal tissues—demonstrates that TransProPy consistently achieves the highest composite scores in both Hallmark and KEGG enrichment analyses, confirming its broad applicability and robustness.

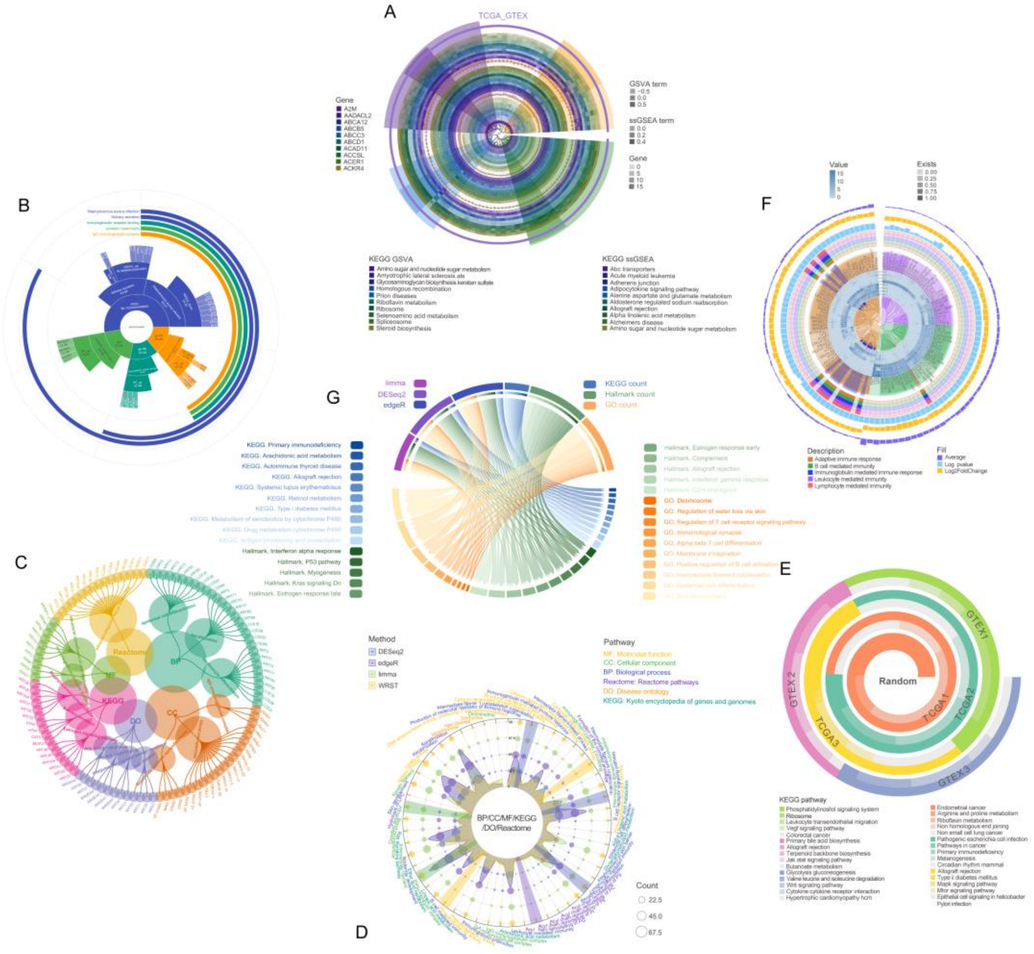

Figure 6. TransProR visualization outputs integrating gene expression patterns, pathway enrichment results, and cross-method comparisons within a single coordinated view. a Multi-layer circular plot integrating differential gene expression with pathway enrichment analysis. Concentric rings display gene expression levels (TCGA vs GTEX) and pathway activities (ssGSEA/GSVA), with color gradients indicating expression magnitude and pathway enrichment scores. **b** Circular enrichment plot displaying pathway categories (KEGG, Disease

Ontology, GO terms) with radial bar lengths proportional to gene counts. Inner rings facilitate systematic comparison across pathway categories. **c** Gene-pathway network diagram showing relationships between differentially expressed genes and biological pathways. Radial organization displays pathway categories (center) extending to associated genes (periphery). **d** Polar bubble plot comparing enrichment results across four statistical methods (DESeq2, edgeR, limma, WRST) and six pathway databases. Bubble size represents gene count; colored sectors distinguish pathway categories. **e** Spiral heatmap visualizing pathway activities across sample groups (TCGA, GTEX). Color intensity indicates KEGG pathway activity levels, enabling cross-sample comparison of pathway dynamics. **f** Hierarchical clustering tree with concentric heatmaps displaying gene relationships and multi-dimensional statistical data. Color-coded layers represent fold changes, p-values, and expression averages across datasets. **g** Circos plot linking differential expression methods (limma, DESeq2, edgeR) to pathway databases (KEGG, Hallmark, GO). Arrow thickness indicates gene contribution; colors distinguish pathway categories.

TransProR complements these analytical capabilities by transforming heterogeneous transcriptomic data into standardized expression matrices through automated preprocessing, batch-effect correction, and multi-method differential expression analysis. Seven multidimensional visualizations constitute its core outputs: (i) multi-layered radial plots displaying gene expression levels alongside pathway activity scores across datasets (Figure 6a) [34-36]; (ii) circular bar charts consolidating GO [37] and KEGG [29, 38] enrichment categories within a single compact display (Figure 6b); (iii) hierarchical network diagrams revealing gene–pathway associations and inter-pathway connectivity (Figure 6c); (iv) polar bubble plots comparing enrichment consistency across statistical methods, with bubble size proportional to gene counts (Figure 6d); (v) spiral heatmaps tracking pathway activity dynamics across experimental conditions (Figure 6e) [39-41]; (vi) integrated circular displays combining clustering dendrograms, expression heatmaps, and enrichment bar charts (Figure 6f); and (vii) chord diagrams quantifying method-specific contributions to shared pathway enrichment (Figure 6g) [42]. Together, TransProPy and TransProR bridge machine learning-driven feature selection with publication-ready biological interpretation, establishing a unified framework that enhances

the robustness, precision, and accessibility of transcriptomic analysis.

Availability of Source Code and Requirements

Project name: TransProR and TransProPy

Project home page: TransProR: https://github.com/SSSYDYSSS/TransProR; TransProPy: https://github.com/SSSYDYSSS/TransProPy

Operating system(s): tested on macOS, Windows

Programming language: R and Python

Other requirements: R (≥ 4.3.0), Python (≥ 3.9)

License: TransProR: MIT; TransProPy: BSD 3-Clause

Data Availability

The source code for TransProR (R) and TransProPy (Python) is publicly available. Detailed user documentation is provided in TransProRBook [43] for TransProR and TransProPyBook [44] for TransProPy. The raw data, analysis pipeline, intermediate results, and documentation source files (Quarto) have been deposited in Zenodo [45]. These resources support reproducibility and facilitate the application of the methodologies presented in this study.

Ethics Statement

The study relies on publicly available datasets that were collected in compliance with ethical standards.

Acknowledgements

The logos for TransProR and TransProPy were generated using ChatGPT [46].

Funding

This work was funded by the National Natural Science Foundation of China [62473212, 62203236,

32130014].

Competing Interests

All authors declare that they have no competing interests.

References

1. Wang Z, Gerstein M and Snyder M. RNA-Seq: a revolutionary tool for transcriptomics. Nat Rev Genet. 2009;10 1:57-63. doi:10.1038/nrg2484.
2. Argelaguet R, Cuomo ASE, Stegle O and Marioni JC. Computational principles and challenges in single-cell data integration. Nat Biotechnol. 2021;39 10:1202-15. doi:10.1038/s41587-021-00895-7.
3. Cao J, O'Day DR, Pliner HA, Kingsley PD, Deng M, Daza RM, et al. A human cell atlas of fetal gene expression. Science. 2020;370 6518:eaba7721. doi:10.1126/science.aba7721.
4. Argelaguet R, Clark SJ, Mohammed H, Stapel LC, Krueger C, Kapourani C-A, et al. Multi-omics profiling of mouse gastrulation at single-cell resolution. Nature. 2019;576 7787:487-91. doi:10.1038/s41586-019-1825-8.
5. Allison DB, Cui X, Page GP and Sabripour M. Microarray data analysis: from disarray to consolidation and consensus. Nat Rev Genet. 2006;7 1:55-65. doi:10.1038/nrg1749.
6. Love MI, Huber W and Anders S. Moderated estimation of fold change and dispersion for RNA-seq data with DESeq2. Genome Biol. 2014;15 12:550. doi:10.1186/s13059-014-0550-8.
7. Robinson MD, McCarthy DJ and Smyth GK. edgeR: a Bioconductor package for differential expression analysis of digital gene expression data. Bioinformatics. 2010;26 1:139-40. doi:10.1093/bioinformatics/btp616.
8. Ritchie ME, Phipson B, Wu D, Hu Y, Law CW, Shi W, et al. limma powers differential expression analyses for RNA-sequencing and microarray studies. Nucleic Acids Res. 2015;43 7:e47-e. doi:10.1093/nar/gkv007.
9. Smyth GK. limma: Linear Models for Microarray Data. Springer-Verlag; 2005. p. 397-420.
10. Li Y, Ge X, Peng F, Li W and Li JJ. Exaggerated false positives by popular differential expression methods when analyzing human population samples. Genome Biol. 2022;23 1:79. doi:10.1186/s13059-022-02648-4.
11. Su Y, Du K, Wang J, Wei J-M and Liu J. Multi-variable AUC for sifting complementary features and its biomedical application. Brief Bioinform. 2022;23 2:bbac029. doi:10.1093/bib/bbac029.
12. Chen X-W and Wasikowski M. FAST: a roc-based feature selection metric for small samples and imbalanced data classification problems. In: 2008, pp.124 - 32. ACM.
13. Wang R and Tang K. Feature Selection for Maximizing the Area Under the ROC Curve. In: 2009 2009, IEEE.
14. Sun L, Wang J and Wei J. AVC: Selecting discriminative features on basis of AUC by maximizing variable complementarity. BMC Bioinformatics. 2017;18 S3:50. doi:10.1186/s12859-017-1468-4.
15. Robnik-Šikonja M and Kononenko I. Theoretical and Empirical Analysis of ReliefF and RReliefF. Machine Learning. 2003;53 1/2:23-69. doi:10.1023/a:1025667309714.
16. Hanchuan P, Fuhui L and Ding C. Feature selection based on mutual information criteria of max-

dependency, max-relevance, and min-redundancy. IEEE Transactions on Pattern Analysis and Machine Intelligence. 2005;27 8:1226-38. doi:10.1109/tpami.2005.159.

17. Wang J, Wei J-M, Yang Z and Wang S-Q. Feature Selection by Maximizing Independent Classification Information. IEEE Transactions on Knowledge and Data Engineering. 2017;29 4:828-41. doi:10.1109/tkde.2017.2650906.

18. Tibshirani R, Hastie T, Narasimhan B and Chu G. Diagnosis of multiple cancer types by shrunken centroids of gene expression. Proceedings of the National Academy of Sciences. 2002;99 10:6567-72. doi:10.1073/pnas.082099299.

19. Weinstein JN, Collisson EA, Mills GB, Shaw KRM, Ozenberger BA, Ellrott K, et al. The Cancer Genome Atlas Pan-Cancer analysis project. Nat Genet. 2013;45 10:1113-20. doi:10.1038/ng.2764.

20. Erickson N, Mueller J, Shirkov A, Zhang H, Larroy P, Li M, et al. AutoGluon-Tabular: Robust and Accurate AutoML for Structured Data. 2020; doi:10.48550/arxiv.2003.06505.

21. Kotthoff L, Thornton C, Hoos HH, Hutter F and Leyton-Brown K. Auto-WEKA: Automatic Model Selection and Hyperparameter Optimization in WEKA. Springer International Publishing; 2019. p. 81-95.

22. Thornton C, Hutter F, Hoos HH and Leyton-Brown K. Auto-WEKA: combined selection and hyperparameter optimization of classification algorithms. ACM, 2013.

23. Feurer M, Springenberg JT and Hutter F. Using meta-learning to initialize bayesian optimization of hyperparameters. In: *MLAS'14* 2014, pp.3-10.

24. Olson RS and Moore JH. TPOT: A Tree-Based Pipeline Optimization Tool for Automating Machine Learning. Springer International Publishing; 2019. p. 151-60.

25. Lonsdale J, Thomas J, Salvatore M, Phillips R, Lo E, Shad S, et al. The Genotype-Tissue Expression (GTEx) project. Nat Genet. 2013;45 6:580-5. doi:10.1038/ng.2653.

26. Subramanian A, Tamayo P, Mootha VK, Mukherjee S, Ebert BL, Gillette MA, et al. Gene set enrichment analysis: A knowledge-based approach for interpreting genome-wide expression profiles. Proceedings of the National Academy of Sciences. 2005;102 43:15545-50. doi:10.1073/pnas.0506580102.

27. Reimand J, Isserlin R, Voisin V, Kucera M, Tannus-Lopes C, Rostamianfar A, et al. Pathway enrichment analysis and visualization of omics data using g:Profiler, GSEA, Cytoscape and EnrichmentMap. Nat Protoc. 2019;14 2:482-517. doi:10.1038/s41596-018-0103-9.

28. Kanehisa M. Toward understanding the origin and evolution of cellular organisms. Protein Sci. 2019;28 11:1947-51. doi:10.1002/pro.3715.

29. Kanehisa M, Furumichi M, Tanabe M, Sato Y and Morishima K. KEGG: new perspectives on genomes, pathways, diseases and drugs. Nucleic Acids Res. 2017;45 D1:D353-D61. doi:10.1093/nar/gkw1092.

30. Kanehisa M, Goto S, Sato Y, Furumichi M and Tanabe M. KEGG for integration and interpretation of large-scale molecular data sets. Nucleic Acids Res. 2012;40 D1:D109-D14. doi:10.1093/nar/gkr988.

31. Kanehisa M. KEGG: Kyoto Encyclopedia of Genes and Genomes. Nucleic Acids Res. 2000;28 1:27-30. doi:10.1093/nar/28.1.27.

32. Liberzon A, Subramanian A, Pinchback R, Thorvaldsdóttir H, Tamayo P and Mesirov JP. Molecular signatures database (MSigDB) 3.0. Bioinformatics. 2011;27 12:1739-40. doi:10.1093/bioinformatics/btr260.

33. Liberzon A, Birger C, Thorvaldsdóttir H, Ghandi M, Mesirov JP and Tamayo P. The Molecular Signatures Database Hallmark Gene Set Collection. Cell Systems. 2015;1 6:417-25. doi:10.1016/j.cels.2015.12.004.
34. Yu G, Lam TT-Y, Zhu H and Guan Y. Two Methods for Mapping and Visualizing Associated Data on Phylogeny Using Ggtree. Mol Biol Evol. 2018;35 12:3041-3. doi:10.1093/molbev/msy194.
35. Xu S, Dai Z, Guo P, Fu X, Liu S, Zhou L, et al. ggtreeExtra: Compact Visualization of Richly Annotated Phylogenetic Data. Mol Biol Evol. 2021;38 9:4039-42. doi:10.1093/molbev/msab166.
36. Xu S, Li L, Luo X, Chen M, Tang W, Zhan L, et al. Ggtree: A serialized data object for visualization of a phylogenetic tree and annotation data. iMeta. 2022;1 4:e56. doi:10.1002/imt2.56.
37. Consortium TGO. Expansion of the Gene Ontology knowledgebase and resources. Nucleic Acids Res. 2017;45 D1:D331-D8. doi:10.1093/nar/gkw1108.
38. Kanehisa M, Furumichi M, Sato Y, Kawashima M and Ishiguro-Watanabe M. KEGG for taxonomy-based analysis of pathways and genomes. Nucleic Acids Res. 2023;51 D1:D587-D92. doi:10.1093/nar/gkac963.
39. Barbie DA, Tamayo P, Boehm JS, Kim SY, Moody SE, Dunn IF, et al. Systematic RNA interference reveals that oncogenic KRAS-driven cancers require TBK1. Nature. 2009;462 7269:108-12. doi:10.1038/nature08460.
40. Hänzelmann S, Castelo R and Guinney J. GSVA: gene set variation analysis for microarray and RNA-Seq data. BMC Bioinformatics. 2013;14 1:7. doi:10.1186/1471-2105-14-7.
41. Gu Z and Hübschmann D. spiralize: an R package for visualizing data on spirals. Bioinformatics. 2022;38 5:1434-6. doi:10.1093/bioinformatics/btab778.
42. Gu Z, Gu L, Eils R, Schlesner M and Brors B. circlize implements and enhances circular visualization in R. Bioinformatics. 2014;30 19:2811-2. doi:10.1093/bioinformatics/btu393.
43. TransProRBook. https://sssydysss.github.io/TransProRBook/. Accessed 30 Jan 2026.
44. TransProPyBook. https://sssydysss.github.io/TransProPyBook/. Accessed 30 Jan 2026.
45. Complete Initial Data for TransProPyBook and TransProRBook: Full Reproduction Using Scripts from Both Manuals. https://zenodo.org/records/14561230. Accessed 30 Jan 2026.
46. OpenAI. https://chat.openai.com. Accessed 25 Jan 2026.

Click here to access/download
Supplementary Material
supplementary materials.pdf

Cover Letter Click here to access/download;Personal Cover;cover letter.pdf

Dear Editors of *GigaScience*,

We would like to submit our manuscript entitled "Comparative Evaluation of Differential Gene Selection Methods in Transcriptomics: Bias Correction and Visualization with TransPro" for your consideration to be published in *GigaScience*.

Background and Motivation: Differential gene selection constitutes the foundation of transcriptomic research; however, different analytical methods frequently yield inconsistent or even contradictory results, raising fundamental concerns regarding reproducibility and biological validity. Although individual methods have been extensively characterized, systematic cross-method comparisons under unified evaluation criteria remain notably absent. This gap impedes researchers from understanding how methodological choices shape downstream biological conclusions.

Our Contribution: This study presents a comprehensive comparative evaluation of widely-adopted differential gene selection methods (including DESeq2, edgeR, limma, and Wilcoxon rank-sum test) within a standardized analytical framework, revealing that different methods exhibit distinct directional biases in gene-pathway associations, with these imbalances cascading through downstream analyses. To address these findings, we developed TransProPy, which evaluates gene complementarity under multivariate AUC and incorporates ensemble machine learning methods, achieving more balanced correlation distributions and pathway enrichment while identifying "super core enriched genes" recurrently appearing across multiple pathways. Comprehensive validation across 12 independent datasets (encompassing multiple cancer types and normal tissues from GTEx, CPTAC, TCGA, and TARGET with diverse origins, batch sources, and sample sizes) demonstrated that TransProPy consistently achieved superior composite scores in both Hallmark and KEGG pathway enrichment analyses, confirming its reliability and generalizability across heterogeneous biological contexts.

Building upon this foundation, TransProR translates these balanced outputs into interpretable biological narratives through seven specialized workflows and multidimensional visualizations (circular dendrograms, chord diagrams, spiral plots, and interaction networks), completing the analytical pipeline from bias correction to biological interpretation.

Significance: The principal contribution of this work lies in establishing a unified comparative framework that enables researchers to systematically assess method-specific biases and make informed analytical decisions. By integrating bias-correcting computation with intuitive visualization, TransPro bridges the divide between computational discovery and biological interpretation. We believe this systematic comparative perspective, coupled with practical correction tools, offers substantive value to the transcriptomics community and aligns with the scope of this journal.

Availability: TransPro (comprising TransProR and TransProPy) is freely available through CRAN and GitHub; reflecting its utility and community adoption, the project has already **received over 450 stars on GitHub** and is supported by comprehensive documentation. This manuscript represents original work approved by all authors.

We deeply appreciate your consideration. We provide a list of potential reviewers for your consideration. Should you have any questions, please do not hesitate to contact us.

Sincerely,

Wenjun Bu, Professor
College of Life Sciences, Nankai University

Potential reviewers:

1) Dr. Juntao Liu (juntao@sdu.edu.cn, juntaosdu@126.com). Dr. Liu is a Professor and Doctoral Supervisor at the School of Mathematics and Statistics, Shandong University, China. His research focuses on bioinformatics algorithms for biomedical data, applying deep learning, graph theory, and combinatorial optimization to transcriptome splicing, chromatin interaction prediction, and cancer driver gene identification. Representative publications include *Genome Biol* 2025 (doi.org/10.1186/s13059-025-03820-2) and *Gigascience* 2024 (doi.org/10.1093/gigascience/giae080). His expertise in transcriptomic modeling and ML-based genomic analysis makes him well-suited to evaluate the methodological rigor and practical utility of our comparative transcriptomics framework.

2) Dr. Hao Wu (wuhao@suat-sz.edu.cn). Dr. Wu is a Professor at the Shenzhen Institute of Advanced Technology, Chinese Academy of Sciences, China. His research focuses on differential methylation analysis and machine learning, with applications in single-cell and spatial transcriptomics. Representative publications include *Biostatistics* 2013 (doi.org/10.1093/biostatistics/kxs033), *Genome Biol* 2024 (doi.org/10.1186/s13059-024-03410-8), and *Nat Commun* 2024 (doi.org/10.1038/s41467-024-52445-9). Given his expertise in differential expression analysis, which aligns closely with our work, and his experience in transcriptomic analysis and machine learning, he is well-suited to assess the analytical validity and biological interpretability of our comparative transcriptomics framework.

3) Dr. Jie Wang (Jiewangcau@cau.edu.cn). Dr. Wang is an Associate Professor at China Agricultural University, China. His research focuses on transcriptomic and metabolomic analysis across diverse sample types and conditions. Representative publications include *ImetaOmics 2024* (doi.org/10.1002/imo2.48), *Environ Sci Technol* 2025a&b (doi.org/10.1021/acs.est.5c07544, doi.org/10.1021/acs.est.5c12104) and *Adv Sci* 2025 (doi.org/10.1002/advs.202409585). His experience in multi-omic data processing and interpretation makes him well-suited to evaluate the practical utility and biological interpretability of our comparative transcriptomics framework from an applied researcher's perspective.

4) Dr. Xubin Zheng (xbzheng@gbu.edu.cn). Dr. Zheng is an Assistant Professor in the School of Computing and Information Technology at Great Bay University, China. His research focuses on multi-omics and multiscale integration with deep learning and machine learning. Representative publications include *PLoS Comput Biol* 2024 (doi.org/10.1371/journal.pcbi.1012083), *AAAI* 2025 (doi.org/10.1609/aaai.v39i1.32010), and *Brief Bioinform* 2025 (doi.org/10.1093/bib/bbaf010). His expertise in multi-omic analysis and ML-based genomic modeling makes him well-suited to evaluate the methodological design and computational robustness of our comparative transcriptomics framework.

5) Dr. Xiaofan Lu (lux@igbmc.fr). Dr. Lu is a Researcher in the Department of Cancer and Functional Genomics, Institute of Genetics and Molecular and Cellular Biology (IGBMC), CNRS/INSERM/UNISTRA, Illkirch, France. His research focuses on multi-omics integration to dissect tumor heterogeneity and cell plasticity, and he has experience in developing multi-omics integration tools. Representative publications include *NPJ Precis Oncol* 2025 (doi.org/10.1038/s41698-025-00912-x), *J Immunother Cancer* 2025 (doi.org/10.1136/jitc-2024-010386), *Clin Transl Med* 2024 (doi.org/10.1002/ctm2.1606), *Cell Rep Med* 2023 (doi.org/10.1016/j.xcrm.2023.101287), and *Nat Commun* 2023 (doi.org/10.1038/s41467-023-43290-3). His expertise in multi-omic integration, tumour biology, and computational tool development makes him well-suited to evaluate the methodological design, visualisation strategy, and biological interpretability of our comparative transcriptomics framework.
