# Supplementary material for: Comparative evaluation of gene selection approaches in transcriptomics: bias correction and visualization with TransPro
Source: Gigascience. 2026 May 18;15:giag057. doi: 10.1093/gigascience/giag057 (PMC13215095; doi:10.1093/gigascience/giag057)
Supplement: giag057_Reviewer_1_Report_original_submission — Reviewer 1 -- 3/26/2026 [file giag057_reviewer_1_report_original_submission.pdf]

Reviewer Report

Title: Comparative Evaluation of Gene Selection Approaches in Transcriptomics: Bias Correction and Visualization with TransPro

Version: Original Submission **Date:** 3/26/2026

Reviewer name: Reviewer 1

Reviewer Comments to Author:

This manuscript attempts to address potential directional bias in differential gene selection from a method-comparison perspective, and further extends this issue to pathway-level interpretive consistency and result presentation. The authors present a relatively complete analytical workflow centered on TransProPy and TransProR, and the overall rationale is generally clear, with substantial visual documentation throughout the manuscript. After reading the paper in full, I believe the study has methodological value. However, several aspects could still be improved, particularly regarding the definition of key concepts, the explanation of figures, and the transparency of certain methodological details. I therefore offer the following comments for the authors' consideration.

1. The concept of "bias correction" would benefit from a brief clarification.
The title and abstract both emphasize "bias correction," which appears to be one of the main selling points of the manuscript. It would be helpful for the authors to clarify that the "bias" discussed here primarily refers to directional selection bias driven by correlation structure, rather than the traditional statistical notion of unbiased estimation. A brief definition in the abstract or discussion would help avoid conceptual ambiguity.

2. The logical transition between Figures 2 and 3 could be strengthened.
The progression from gene-level correlation balance in Figure 2 to pathway-level consistency in Figure 3 is in fact quite clear. However, the narrative would be smoother if the authors added a bridging sentence in the Results to make this transition explicit, for example by stating that "gene-level balance is further examined at the pathway level."

3. The visual elements in Figure 3B should be explained more clearly.
In Figure 3B, some genes are represented not only by scatter points but also by bar-like elements. In addition, the lengths of these bars, the number of colors they contain, and their transparency levels appear to vary, while some genes do not show such elements at all. The authors should explain in the main text or figure legend what these visual encodings represent and what information they are intended to convey, so that readers can interpret the figure without confusion.

4. The symbols used in Figure 3C should be defined explicitly in the legend.
The meaning of the check marks and cross marks in Figure 3C is not immediately obvious. It would be helpful to state clearly in the figure legend what each symbol represents.

5. The discussion of pathway redundancy could be expanded slightly.
The authors note that some suppressed pathways are highly similar in gene composition and ranking, which is an important observation. It would strengthen the Discussion to add a sentence noting that an apparent numerical advantage in enriched pathways does not necessarily correspond to richer biological

insight when substantial pathway redundancy is present.
6. Terminology, capitalization, and singular/plural usage should be checked for consistency.
The authors may wish to review the use of terms such as Hallmark/Hallmarks and KEGG pathway/gene set throughout both the main text and the figure legends, in order to ensure consistency in capitalization and singular/plural form.
7. The construction of the scoring system requires clearer methodological description.
Figure 4d presents Hallmark score, KEGG score, and Total score across 12 independent datasets, but the manuscript does not sufficiently explain how these scores are calculated. It is currently unclear whether the scoring is based on deviation from pathway activation/suppression balance, the number of successfully enriched pathways, or some weighted composite metric. Because this scoring system serves as a central measure in the cross-dataset validation, the Methods section should provide a clear mathematical definition of how these scores are derived.

Methods

Are the methods appropriate to the aims of the study, are they well described, and are necessary controls included? Choose an item.

Conclusions

Are the conclusions adequately supported by the data shown? Choose an item.

Reporting Standards

Does the manuscript adhere to the journal's guidelines on [minimum standards of reporting?]() Choose an item.

Choose an item.

Statistics

Are you able to assess all statistics in the manuscript, including the appropriateness of statistical tests used? Choose an item.

Quality of Written English

Please indicate the quality of language in the manuscript: Choose an item.

Declaration of Competing Interests

Please complete a declaration of competing interests, considering the following questions:

- Have you in the past five years received reimbursements, fees, funding, or salary from an organisation that may in any way gain or lose financially from the publication of this manuscript, either now or in the future?
- Do you hold any stocks or shares in an organisation that may in any way gain or lose financially from the publication of this manuscript, either now or in the future?
- Do you hold or are you currently applying for any patents relating to the content of the manuscript?
- Have you received reimbursements, fees, funding, or salary from an organization that holds or has applied for patents relating to the content of the manuscript?
- Do you have any other financial competing interests?
- Do you have any non-financial competing interests in relation to this paper?

If you can answer no to all of the above, write 'I declare that I have no competing interests' below. If your reply is yes to any, please give details below.

No

I agree to the open peer review policy of the journal. I understand that my name will be included on my report to the authors and, if the manuscript is accepted for publication, my named report including any attachments I upload will be posted on the website along with the authors' responses. I agree for my report to be made available under an Open Access Creative Commons CC-BY license (http://creativecommons.org/licenses/by/4.0/). I understand that any comments which I do not wish to be included in my named report can be included as confidential comments to the editors, which will not be published.

I agree to the open peer review policy of the journal

To further support our reviewers, we have joined with Publons, where you can gain additional credit to further highlight your hard work (see: https://publons.com/journal/530/gigascience). On publication of this paper, your review will be automatically added to Publons, you can then choose whether or not to claim your Publons credit. I understand this statement.

Yes Choose an item.
