# Supplementary material for: Comparative evaluation of gene selection approaches in transcriptomics: bias correction and visualization with TransPro
Source: Gigascience. 2026 May 18;15:giag057. doi: 10.1093/gigascience/giag057 (PMC13215095; doi:10.1093/gigascience/giag057)
Supplement: giag057_Reviewer_2_Report_original_submission — Reviewer 2 -- 3/27/2026 [file giag057_reviewer_2_report_original_submission.pdf]

Reviewer Report

Title: Comparative Evaluation of Gene Selection Approaches in Transcriptomics: Bias Correction and Visualization with TransPro

Version: Original Submission **Date:** 3/27/2026

Reviewer name: Reviewer 2

Reviewer Comments to Author:

This manuscript presents TransPro, a dual-package framework (TransProPy in Python and TransProR in R) for differential gene selection in transcriptomics. The authors argue that classical methods (DESeq2, edgeR, limma, WRST) suffer from directional biasâ€”overrepresenting positively correlated genesâ€”and that this bias propagates into pathway enrichment analysis. TransProPy addresses this through mvAUC-based complementarity quantification and ensemble learning, while TransProR provides standardized visualization workflows. The framework is validated across 12 independent datasets spanning multiple cancer types and normal tissues.
The topic is relevant and the bias problem the authors identify is worth investigating. However, several conceptual, methodological, and presentational issues require substantial revision before the manuscript is suitable for publication.
The manuscript conflates two fundamentally different analytical goals throughout. DESeq2, edgeR, limma, and WRST are designed for differential expression analysisâ€”identifying genes whose expression levels change significantly between conditions. TransProPy performs supervised feature selectionâ€—identifying gene subsets that jointly discriminate between phenotypes. These answer different biological questions. Comparing them as if they compete on the same task is problematic. The authors should clearly delineate the distinction in the Introduction and Discussion, and frame the comparison as complementary approaches rather than direct competitors on the same objective. The title itself ("Differential Gene Selection Methods") blurs this distinction.
The evaluation is built around 33 genes selected as the intersection of all six methods. This intersection-based gene set is inherently biased toward genes on which all methods agree, which may not be representative of the broader gene selection landscape. Furthermore, the evaluation metric (correlation balance) is essentially the property that TransProPy is designed to optimize, making the benchmarking somewhat circular. External validation is needed, such as: predictive performance on held-out data (e.g., classification accuracy, AUC on independent test sets), overlap with known driver genes or curated gene sets, survival analysis or other clinical outcome associations, and comparison against established multivariate methods beyond those tested (e.g., LASSO, elastic net, random forests for feature selection).
For a method framed around feature selection and machine learning, there is no evaluation of predictive performance. The manuscript does not report classification accuracy, cross-validation AUC, or any measure of how well TransProPy-selected genes discriminate between conditions on held-out data. This is a critical omission for a tool that integrates ensemble learning and RFECV. At minimum, the authors should show that TransProPy's gene sets perform comparably or better than those from

conventional methods in a classification or prediction task.

The pathway-level analysis relies heavily on visual inspection of ratios and proportions. While Kruskal-Wallis and Wilcoxon tests are mentioned ($p < 0.001$), it is unclear exactly what is being tested, what the sample sizes are for each comparison, and whether multiple testing correction was applied across the many comparisons shown. The 20 repetitions per gene per method (Section 2.3) suggest stochasticity in GSEA, but the source of this stochasticity is not explainedâ€"GSEA with a fixed gene list and fixed gene set should produce deterministic results unless permutation-based p-values are used, in which case the permutation settings should be specified.

The 12-dataset validation (Figure 4d) uses a composite "score" that is not formally defined. The Methods section states scores were computed for Hallmark and KEGG gene sets and aggregated, but the scoring formula is absent. Without knowing how the score is calculated, the reader cannot evaluate whether TransProPy's advantage is meaningful or an artifact of the scoring scheme. Full details of score computation must be provided.

Minor Issues

The manuscript is dense and difficult to follow. Several improvements would help:

- The Introduction (Section 1) is overly long for a Technical Note. The review of MACFC, AutoGluon, Auto-WEKA, and others reads more like a literature review for a full research article. Consider condensing to focus on the specific gap TransPro addresses.
- Figures are visually complex but lack clarity. Figure 1 (schematic) is too crowded to be informative at publication scale. Figures 2-5 pack many panels into single figures, making individual panels very small. Consider splitting key results into separate, larger figures.
- Figure legends are excessively long, in some cases nearly half a page. They should be concise and direct readers to the Methods for full details.
- The Zenodo deposit (ref 45) should include not just raw data and scripts but also a containerized environment (e.g., Docker) or at minimum a complete environment specification (conda/pip requirements with pinned versions) to ensure reproducibility.
- Version numbers for TransProPy and TransProR are not stated. Are these version 1.0 releases?
- The relationship between AutoFS (described in Methods 3.2) and MACFCv2 (Methods 3.1) within TransProPy needs clearer explanation. When should a user choose one over the other? The manuscript mentions both are used but the decision logic is unclear.

The comparison is limited to four classical DE methods. Several relevant multivariate or interaction-aware methods are not discussed or compared, including WGCNA (weighted gene co-expression network analysis), various penalized regression approaches (LASSO, elastic net), and modern deep learning-based feature selection methods. Justification for the chosen comparators is needed.

The concept of "super core enriched genes" (Section 2.5) is introduced without formal definition. A gene appearing in the leading edge of multiple pathways is expected given pathway overlap (e.g., KEGG pathways share many genes). The amplification effect described may simply reflect known pathway redundancy rather than a novel biological insight. The authors should clarify what this concept adds beyond established notions of pathway crosstalk.

Section 3.1 states that MACFCv2 applies an AUC threshold for preselection, but the threshold value is not specified.

The Spearman correlation threshold of |Ï�| > 0.5 is somewhat arbitrary. Sensitivity analysis across

different thresholds would strengthen the findings.
The manuscript mentions testing on macOS and Windows but not Linux, which is the dominant platform for bioinformatics.

Methods

Are the methods appropriate to the aims of the study, are they well described, and are necessary controls included? Choose an item.

Conclusions

Are the conclusions adequately supported by the data shown? Choose an item.

Reporting Standards

Does the manuscript adhere to the journal's guidelines on [minimum standards of reporting?]() Choose an item.

Choose an item.

Statistics

Are you able to assess all statistics in the manuscript, including the appropriateness of statistical tests used? Choose an item.

Quality of Written English

Please indicate the quality of language in the manuscript: Choose an item.

Declaration of Competing Interests

Please complete a declaration of competing interests, considering the following questions:

- Have you in the past five years received reimbursements, fees, funding, or salary from an organisation that may in any way gain or lose financially from the publication of this manuscript, either now or in the future?
- Do you hold any stocks or shares in an organisation that may in any way gain or lose financially from the publication of this manuscript, either now or in the future?
- Do you hold or are you currently applying for any patents relating to the content of the manuscript?
- Have you received reimbursements, fees, funding, or salary from an organization that holds or has applied for patents relating to the content of the manuscript?
- Do you have any other financial competing interests?
- Do you have any non-financial competing interests in relation to this paper?

If you can answer no to all of the above, write 'I declare that I have no competing interests' below. If your reply is yes to any, please give details below.

I declare that I have no competing interests

I agree to the open peer review policy of the journal. I understand that my name will be included on my report to the authors and, if the manuscript is accepted for publication, my named report including any attachments I upload will be posted on the website along with the authors' responses. I agree for my report to be made available under an Open Access Creative Commons CC-BY license (http://creativecommons.org/licenses/by/4.0/). I understand that any comments which I do not wish to be included in my named report can be included as confidential comments to the editors, which will not be published.

I agree to the open peer review policy of the journal

To further support our reviewers, we have joined with Publons, where you can gain additional credit to further highlight your hard work (see: https://publons.com/journal/530/gigascience). On publication of this paper, your review will be automatically added to Publons, you can then choose whether or not to claim your Publons credit. I understand this statement.

Yes Choose an item.
