# Supplementary material for: Comparative evaluation of gene selection approaches in transcriptomics: bias correction and visualization with TransPro
Source: Gigascience. 2026 May 18;15:giag057. doi: 10.1093/gigascience/giag057 (PMC13215095; doi:10.1093/gigascience/giag057)
Supplement: giag057_Reviewer_3_Report_original_submission — Reviewer 3 -- 3/28/2026 [file giag057_reviewer_3_report_original_submission.pdf]

Reviewer Report

Title: Comparative Evaluation of Gene Selection Approaches in Transcriptomics: Bias Correction and Visualization with TransPro

Version: Original Submission **Date:** 3/28/2026

Reviewer name: Reviewer 3

Reviewer Comments to Author:

General Assessment
This manuscript addresses the important issue of method-specific bias in differential gene screening within transcriptomics, and proposes the TransPro framework. By integrating TransProPy and TransProR, the authors present a coherent workflow spanning gene screening, bias correction, enrichment analysis, and multi-dimensional visualization. The topic is of practical relevance, the methodological integration is well-structured, and the results are richly presented. In particular, the cross-dataset validation and multi-level visualization components enhance the practical value and reproducibility of this work. Overall, I believe this study has clear novelty and practical potential.
Specific Comments
1. Regarding the "33 commonly screened genes," I recommend adding a brief explanation of the rationale behind this intersection-based design. For example, the authors could clarify that its primary purpose is to ensure fairness and interpretability in subsequent comparisons, which would help avoid potential reader confusion.
2. In Figure 2B, the results across all five methods appear largely similar. Could the authors clarify the intended purpose of this panel? Is the consistency in trends a prerequisite for the subsequent comparative analyses, or does it serve to demonstrate that TransProPy retains the strengths already possessed by existing methods?
3. In Figure 2C, the line trend plot at the bottom is not described in the main text. I suggest the authors provide a brief explanation of how this plot was generated and what it is intended to convey.
4. The two-package design (TransProPy and TransProR) is one of the strengths of this work. To further improve readability, the authors may consider more explicitly distinguishing the primary role of each package at the beginning of the Methods section.
5. Certain expressions such as "universal superiority" or "the only method" could be toned down for academic rigor. For example, "superior performance across the evaluated datasets" or "the only method among those evaluated here" would be more precise.
Minor language suggestions:
6. "The +/âˆ’ NES signifies if the normalized enrichment score is positive..." â€” signifies if is slightly unnatural; indicates whether is more standard.
7. "both ratios >1 and <1 were observed" â€” this phrasing is somewhat stiff. Consider: "ratios both greater than 1 and less than 1 were observed across cases."
8. This work also includes a substantial visualization component. I recommend the authors briefly summarize from a practical standpoint which analytical scenarios are best suited for the proposed

combinatorial visual displays, and highlight the specific advantages of the visualization design.

Methods

Are the methods appropriate to the aims of the study, are they well described, and are necessary controls included? Choose an item.

Conclusions

Are the conclusions adequately supported by the data shown? Choose an item.

Reporting Standards

Does the manuscript adhere to the journal's guidelines on minimum standards of reporting? Choose an item.

Choose an item.

Statistics

Are you able to assess all statistics in the manuscript, including the appropriateness of statistical tests used? Choose an item.

Quality of Written English

Please indicate the quality of language in the manuscript: Choose an item.

Declaration of Competing Interests

Please complete a declaration of competing interests, considering the following questions:

- Have you in the past five years received reimbursements, fees, funding, or salary from an organisation that may in any way gain or lose financially from the publication of this manuscript, either now or in the future?
- Do you hold any stocks or shares in an organisation that may in any way gain or lose financially from the publication of this manuscript, either now or in the future?
- Do you hold or are you currently applying for any patents relating to the content of the manuscript?
- Have you received reimbursements, fees, funding, or salary from an organization that holds or has applied for patents relating to the content of the manuscript?
- Do you have any other financial competing interests?
- Do you have any non-financial competing interests in relation to this paper?

If you can answer no to all of the above, write 'I declare that I have no competing interests' below. If your reply is yes to any, please give details below.

None

I agree to the open peer review policy of the journal. I understand that my name will be included on my report to the authors and, if the manuscript is accepted for publication, my named report including any attachments I upload will be posted on the website along with the authors' responses. I agree for my report to be made available under an Open Access Creative Commons CC-BY license (http://creativecommons.org/licenses/by/4.0/). I understand that any comments which I do not wish to be included in my named report can be included as confidential comments to the editors, which will not be published.

I agree to the open peer review policy of the journal

To further support our reviewers, we have joined with Publons, where you can gain additional credit to further highlight your hard work (see: https://publons.com/journal/530/gigascience). On publication of this paper, your review will be automatically added to Publons, you can then choose whether or not to claim your Publons credit. I understand this statement.

Yes Choose an item.
